# Development and structure–activity relationships of tanshinones as selective 11*β*-hydroxysteroid dehydrogenase 1 inhibitors

**DOI:** 10.1007/s13659-022-00358-9

**Published:** 2022-09-22

**Authors:** Xu Deng, Su-Ling Huang, Jian Ren, Zheng-Hong Pan, Yu Shen, Hao-Feng Zhou, Zhi-Li Zuo, Ying Leng, Qin-Shi Zhao

**Affiliations:** 1 State Key Laboratory of Phytochemistry and Plant Resources in West China, Kunming Institute of Botany, Chinese Academy of Sciences, Kunming, 650204 China; 2 Xiangya School of Pharmaceutical Sciences, Central South University, Changsha, 410013 China; 3 State Key Laboratory of Drug Research, Shanghai Institute of Materia Medica, Chinese Academy of Sciences, Shanghai, 201203 China; 4 Guangxi Key Laboratory of Functional Phytochemicals Research and Utilization, Guangxi Institute of Botany, Chinese Academy of Sciences, Guilin, 541006 China

**Keywords:** Metabolic syndrome, Tanshinones, Selective 11*β*-HSD1 inhibitors, Structure–activity relationships

## Abstract

11*β*-Hydroxysteroid dehydrogenase 1 (11*β*-HSD1) represents a promising drug target for metabolic syndrome, including obesity and type 2 diabetes. Our initial screen of a collection of natural products from Danshen led to the identification of tanshinones as the potent and selective 11*β*-HSD1 inhibitors. To improve the druggability and explore the structure–activity relationships (SARs), more than 40 derivatives have been designed and synthesized using tanshinone IIA and cryptotanshinone as the starting materials. More than 10 derivatives exhibited potent in vitro 11*β*-HSD1 inhibitory activity and good selectivity over 11*β*-HSD2 across human and mouse species. Based on the biological results, SARs were further discussed, which was also partially rationalized by a molecular docking model of **1** bound to the 11*β*-HSD1. Remarkably, compounds **1**, **17** and **30** significantly inhibited 11*β*-HSD1 in 3T3-L1 adipocyte and in livers of *ob/ob* mice, which merits further investigations as anti-diabetic agents. This study not only provides a series of novel selective 11*β*-HSD1 inhibitors with promising therapeutic potentials in metabolic syndromes, but also expands the boundaries of the chemical and biological spaces of tanshinones.

**Graphic Abstract:**

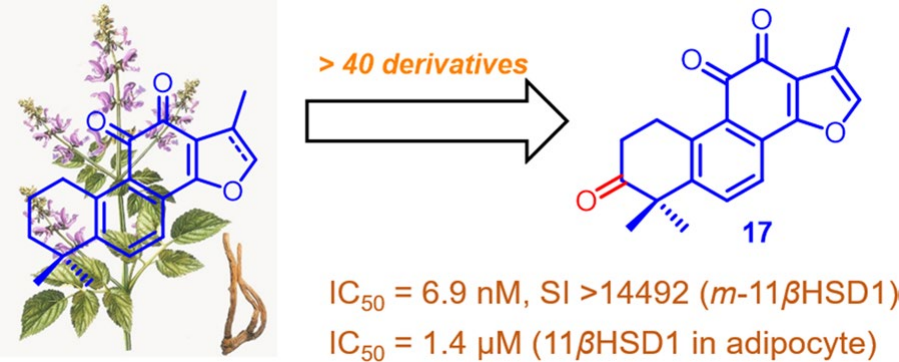

**Supplementary Information:**

The online version contains supplementary material available at 10.1007/s13659-022-00358-9.

## Introduction

Metabolic syndrome is a prediabetic state, which features central obesity, glucose intolerance, insulin resistance, dyslipidemia, hypertension, and inflammatory or prothrombotic state [1]. Metabolic syndrome can progress to type 2 diabetes, which results in serious diabetes complications, including cardiovascular disease, diabetic retinopathy, renal failure, nerve damage and ischemic stroke [2]. It is postulated that aberrant glucocorticoid signaling may contribute to the metabolic syndrome [3]. Increased local concentration of glucocorticoids rather than systematically elevated glucocorticoid levels impairs insulin and leptin sensitivity, which ultimately results in metabolic disease, including Cushing’s syndrome, obesity and type 2 diabetes [4]. These findings suggest that control of intracellular glucocorticoid level (specifically intracellular concentration in the metabolically relevant tissues) is a promising therapy for metabolic syndrome.

**Fig. 1:**
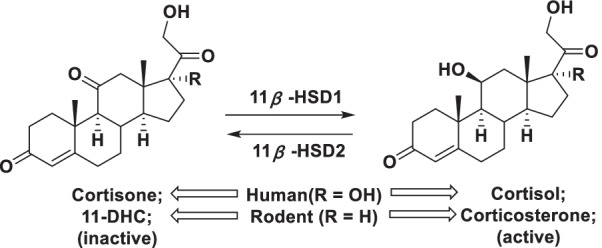
Interconversion of cortisone and cortisol by 11*β*-HSD1 and 11*β*-HSD2 in humans and 11-dihydrocorticosterone (11-DHC) and corticosterone in rodents

The 11*β*-hydroxysteroid dehydrogenase (11*β-*HSD) enzymes, including 11*β-*HSD1 and 11*β-*HSD2, catalyze the inter-conversion between the inactive cortisone (11-dehydrocorticosterone in rodents) and the active cortisol in human (corticosterone in rodents) in a tissue specific manner (Fig. 1) [5]. 11*β-*HSD1 is highly expressed in key metabolic tissues, such as liver and adipose tissue. Transgenic over-expression of 11*β-*HSD1 in mice adipose tissue has caused visceral obesity, glucose intolerance, and insulin resistance [6, 7]. In obese patients, enhanced expression of 11*β-*HSD1 was also observed in adipose tissue and skeletal muscle [8, 9]. In contrast, global 11*β-*HSD1 knockout mice showed improved glucose tolerance, insulin sensitivity, and resistance to obesity when fed a high fat diet [6, 10–12]. On the other hand, 11*β*-HSD2 is predominately expressed in mineralocorticoid target tissues, such as the kidney and colon, which protects mineralocorticoid receptor from excessive activation by cortisol [13–15]. Evidence showed that inhibition of 11*β*-HSD2 would lead to sodium retention, hypokalaemia and hypertension [14, 16]. Accumulating evidence suggests that development of novel potent and selective 11*β*-HSD1 inhibitors is an attractive strategy for anti-diabetic therapy.

**Fig. 2:**
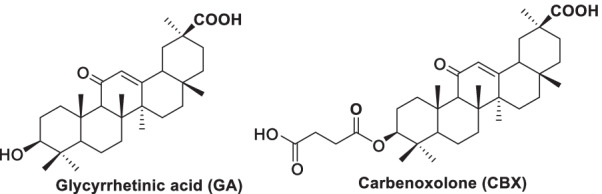
Representatives of nature-derived 11*β-*HSD1 inhibitors

As a result, extensive efforts from the academic and industrial communities have been directed toward the discovery and development of selective 11*β-*HSD1 inhibitors [17–19]. Incyte, Merck, and others have demonstrated that 11*β-*HSD1 inhibitors improved glycemic control, lipid profiles, and blood pressure with modest weight loss [20]. However, none of them have progressed beyond phase 2 trials. Natural products (NPs), especially those derived from traditional Chinese medicines (TCMs), remain untapped sources of molecular scaffolds that can serve as lead compounds and potential therapeutic agents for diverse range of diseases (Fig. 2) [21].

*Salvia miltiorrhiza* Bunge*,* also known as the traditional Chinese medicine “Danshen”, has well-defined effects on cardiovascular and cerebrovascular disease. Extracts of *S. miltiorrhiza* roots in formulation of “Compound Danshen Dripping pill” are undergoing clinical trials in the United States. Up to now, more than 60 tanshinones have been isolated and identified from Danshen and related species [22]. Among them, tanshinone IIA and cryptotanshinone were the most studied because of their high abundance in plants and wide range of biological activities [23–25]. For instance, tanshinone IIA and cryptotanshinone have been shown to increase the activity of insulin by affecting tyrosine phosphorylation of the insulin receptor and activation of the down-stream kinase Akt, ERK1/2, and GSK3β [26] and ameliorated the diabetic complications [27–29]. We recently disclosed the discovery and SARs studies of tanshinones as novel indoleamine 2,3-deoxygenase (IDO1) and tryptophan 2,3-deoxygenase (TDO) dual inhibitors [30]. However, reports on their 11*β*-HSD1 inhibitory activities, as well as their structure–activity relationships, remain scarce, albeit that Park recently disclosed tanshinone IIA as a 11*β*-HSD1 inhibitor [31] following our patents on similar results being published in 2012 (CN 102304166A and CN 102603861A). As our constant efforts on developing naturally occurring therapeutic agents against type 2 diabetes [32, 33], we herein presented the development and structure–activity relationship studies of tanshinones as novel potent and selective 11*β*-HSD1 inhibitors.

## Results

### Discovery of tanshinones as novel 11*β*-HSD1 inhibitors

Our preliminary efforts towards the development of nature-derived 11*β*-HSD1 inhibitors led to the identification of eight active tanshinones (**1**–**8**) among a collection of natural products derived from Danshen and related species (Table 1). As shown in Table 1, most of them exhibited low nanomolar inhibitory activities against both human and mouse 11*β*-HSD1 but no detectable inhibition against 11*β*-HSD2. Remarkably, tanshinone IIA (**1**) and cryptotanshinone (**2**) exerted comparable inhibitory activities against 11*β*-HSD1 to that of the positive control (the nonselective 11βHSD inhibitor glycyrrhizic acid, GA), but with excellent selectivity over 11*β*-HSD2. Moreover, they are readily available. To improve the druggability and further explore their structure–activity relationships, a focused library of tanshinones by using **1** and **2** as the starting materials were thereby designed and synthesized.

**Table 1:** Initial enzymatic assay of tanshinones from *Salvia trijuga*


Entry	Mouse (IC_50_)			Human (IC_50_)		
	11*β*-HSD1	11*β*-HSD2	SI^c^	11*β*-HSD1	11*β*-HSD2	SI^c^
**1**	32.2 ± 2.5 nM	> 1 mM	> 31,250	35.3 ± 15.4 nM	135.4 ± 72.5 µM	3838
**2**	41.8 ± 8.7 nM	> 1 mM	> 23,923	73.1 ± 31.3 nM	12.1 µM	621
**3**	0.4 ± 0.1 µM	–	–	1.0 ± 0.4 µM	–	–
**4**	113.4 ± 44.9 nM	–	–	168.5 ± 6.8 nM	–	–
**5**	392.3 ± 77.6 nM	–	–	206.5 ± 8.9 nM	–	–
**6**	152.2 ± 11.7 nM	–	–	0.5 ± 0.6 µM	–	–
**7**	1.7 ± 0.2 µM	–	–	2.2 ± 0.1 µM	–	–
**8**	4.0 ± 0.5 µM	–	–	12.2 ± 4.2 µM	–	–
**GA**^a^	8.8 ± 2.0 nM	–	–	15.5 ± 5.5 Nm	–	–

Values are averages of two determinations

^a^GA: glycyrrhetinic acid, which was used as a positive control. ^c^SI: selectivity index, SI = [IC_50_(HSD2)/IC_50_(HSD1)]

### Chemistry

#### Scaffold hopping design and synthesis

**Scheme 1:**
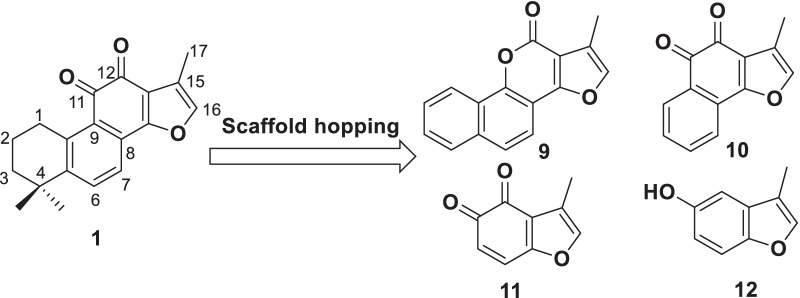
Scaffold hopping design. Analogues **9**–**12** were prepared from commercially available starting materials according to the previously reported procedures [34–36]

To elucidate the SARs of tanshinones, a scaffold hopping strategy was envisaged [34]. As shown in Scheme 1, several truncated tanshinone analogues **9**–**12** were designed. Basically, a “knockout strategy” was applied in designing analogues **9**–**12**. For instance, compound **10** was envisaged by deletion of A-ring in tanshinone IIA (**1**). Further removal of B-ring generates compound **11**. By comparisons of the biological profiles from **1** to **10** and **11** clearly illustrate the impacts of A- and B-ring on the the 11*β*-HSD1 inhibitory activities. In contrast, compounds **9** and **12** were designed to probe the role of *ortho*-quinone moiety. Analogues **9**–**12** were prepared from the commercially available starting materials according to the well-established protocols [35–37].

#### Modifications at A-ring

**Scheme 2:**
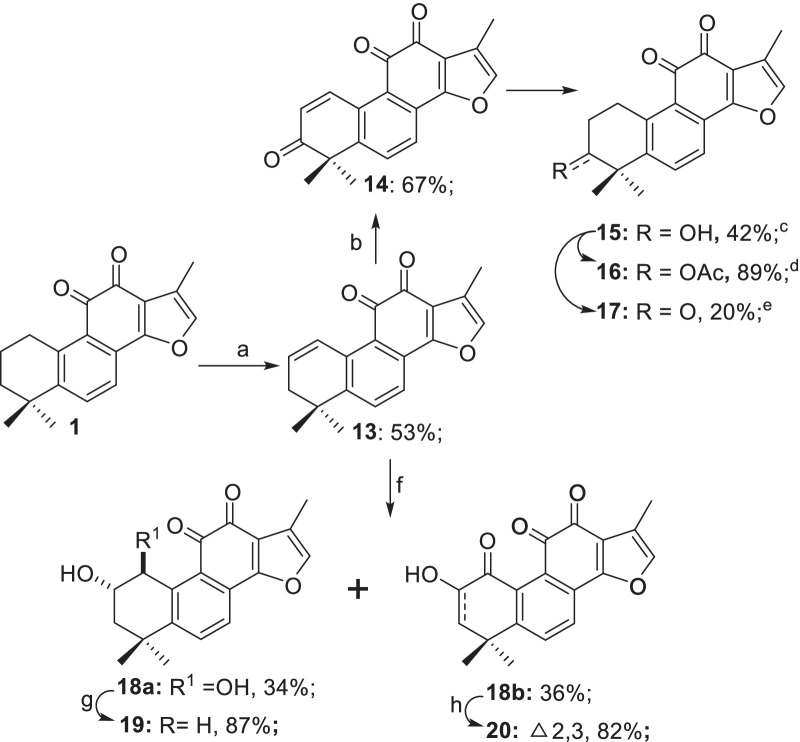
Modifications on A-ring. *Reagents and Conditions:* a) NBS, Benzoyl peroxide, CCl_4_, reflux; b) SeO_2_, 1,4-dioxane, reflux; c) H_2_, Pd/C, MeOH/HCOOH (8:1), rt; d) Ac_2_O, Pyridine, DMAP, DCM, rt; e) PDC, Celite, DCM, rt; f) KMnO_4_, HCOOH, THF/H_2_O (4:1), rt; g) BF_3_^**.**^OEt_2_, *n*-Bu_3_SnH, DCM, 0 °C-rt; h) (COCl)_2_, DMSO, TEA, DCM, − 78 °C-rt

**Scheme 3:**
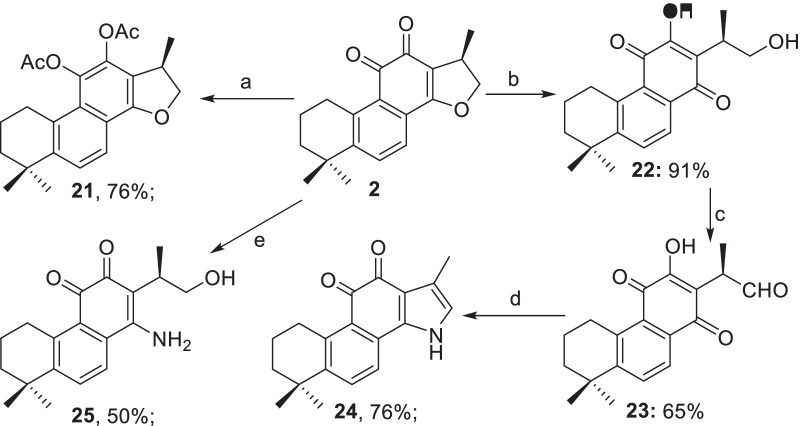
Modifications on C- and D-rings of **2**. *Reagents and Conditions:* a) H_2_, PtO_2_, Ac_2_O, Pyridine, THF, rt; b) NaOH (2.0 M), THF/H_2_O, ultrasound; c) IBX, DMSO, rt; c) NH_4_OAc, HOAc, 60 °C; d) NH_3_-H_2_O, EtOH, rt;

**Scheme 4:**
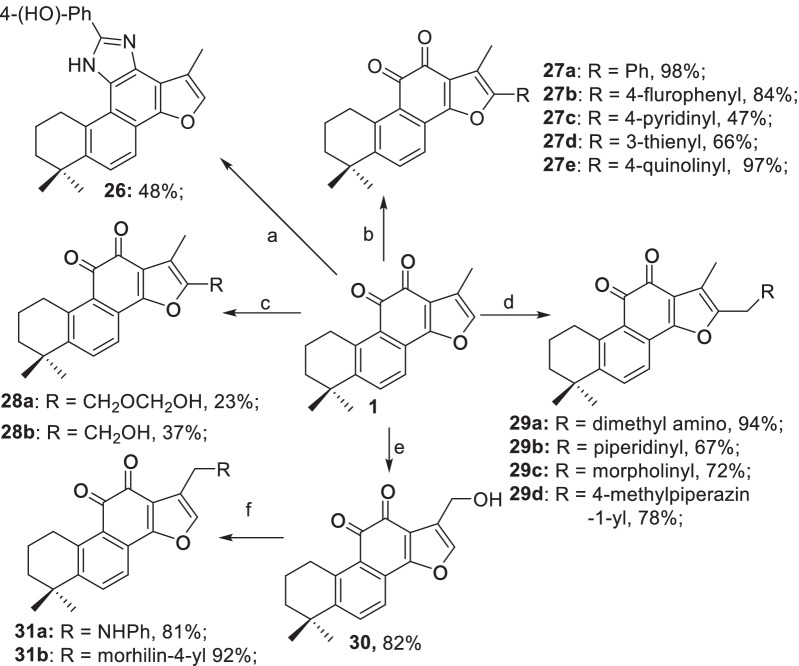
Modifications on C- and D-rings of **1**. *Reagents and Conditions*: a) *p*-Hydroxybenzaldehyde, ammonium formate, AcOH, 100 ^o^C; b) step 1: NIS, TFA, DCM, rt; step 2: ArB(OH)_2_, Pd(PPh_3_)_4_, PPh_3_, K_2_CO_3_, DMF / H_2_O (5:1), 80 °C; c) HCHO (aq.), HCl (con.), H_2_O, reflux; d) (HCHO)n, R_1_R_2_NH, AcOH, 110 °C; e) SeO_2_, AcOH, reflux; f) step 1: CBr_4_, PPh_3_, DCM, rt; step 2: PhNH_2 _or morpholine, Et_3_N, DCM, rt

**Scheme 5:**
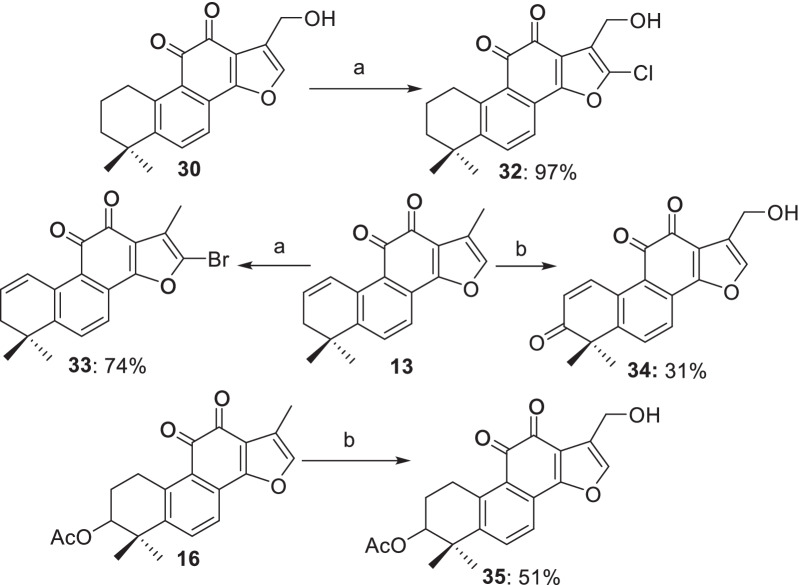
Modifications on multi-positions. *Reagents and Conditions*: a) NCS (or NBS), TFA, DCM, rt; b) SeO_2_, AcOH, Reflux

The chemical synthesis of tanshinone derivatives was depicted in Schemes 2, 3, 4, 5. As described previously [30], the modifications of A-ring commenced with the functionalization of C1 and C2 (Scheme 2). The installation of the alkene at C1, C2 moiety was achieved by reacting **1** with NBS in the presence of benzoyl peroxide, which delivered the alkene **13** in 53% yield, which was further converted to its derivatives via divergent pathways. For instance, SeO_2_-mediated oxidation of **13** in 1,4-dioxane delivered the corresponding α,β-unsaturated ketone **14** in 67% yield, which was further reduced to the C1, C2-saturated derivative **15** via catalytic hydrogenation. **15** was in turn transformed to the 3-acetoxy derivative **16** (89% yield) and 3-oxo derivative **17** (20% yield) by esterification and oxidation, respectively. In parallel, dihydroxylation of **13** with KMnO_4_ in the presence of HCOOH furnished **18a**, **18b** in 34%, 36% yields, respectively. The relative configuration of C1,C2-dihydroxy groups in **18a** was assigned as *trans* by the *J*_H1-H2_ value (6.0 Hz) of its acetonide derivative of **18a** [38]. Cationic reduction of **18a** using tributyltin hydride in the presence of BF_3_^**.**^OEt_2_ gave the C2-OH derivative **19** in 87% yield. Alternatively, Swern oxidation of **18b** delivered the enone **20** in 87% yield (Scheme 2).

#### Modifications at C- and D-rings

Scheme 3 depicts the chemical manipulations on the C- and D-rings of **2**. The C11,C12-diacetoxy derivative **21** was obtained in 81% yield by catalytic hydrogenation of **2** in Ac_2_O in the presence of pyridine. Treatment of **2** with aq. NaOH (2.0 mol/L) with the assistance of ultrasound delivered (+)-neocryptotanshinone **22** in 91% yield [39], which was further oxidized to the corresponding aldehyde **23** in 65% yield. Subsequently, the pyrrol **24** was prepared by treatment of the aldehyde **23** with ammonia following the Paal–Knorr’s pyrroles synthesis [40]. On the other hand, treatment of **2** with aqueous ammonia provided the amine **25** in 50% yield [41] (Scheme 3).

On the other hand, modifications on the C- and D-rings of **1** were illustrated in Scheme 4. A three-component reaction between **1**, *p*-hydroxybenzaldehyde and ammonium formate afforded the imidazole **26** in 48% yield [42]. To explore the effects of the C16-substituents on the 11*β*-HSD1 inhibitory activities and selectivity, a series of C16 functionalized derivatives were designed and synthesized. First, installation of an iodide at C16 by treatment of **1** with *N*-iodosuccinimide (NIS) in the presence of catalytic amount of trifluoroacetic acid delivered the desired product in 79% yield, which was followed by arylation under Suzuki’s protocol to afford the C16-aryl derivatives **27a**–**27e** in 47–98% yields [43]. On the other hand, treatment of **1** with formalin in AcOH furnished the alcohols **28a** and **28b**. Similarly, a series of amines **29a**–**29d** were also prepared by following the Pictet-Spengler’s protocol [35].

Derivatives with diverse functionalities at C17 were also designed and synthesized. Alder ene-type oxidation of the benzylic position with SeO_2_ afforded the alcohol **30** in 82% yield [44], which in turn enabled diverse further transformations. For instance, Corey-Fuch bromination delivered the bromide in 91% yield [45], which reacted with the amines in the presence of base to afford **31a** and **31b** in 81% and 92% yields, respectively.

#### Modifications at multi-positions

Based on the preliminary SARs studies, we envisaged to incorporate multiple privileged scaffolds into one molecular. As a result, a series of multiple position-modified derivatives were designed and synthesized using the privileged derivatives **13** and **30** as the starting materials (Scheme 5). On one hand, chlorination of **30** with *N*-chlorosuccinimide (NCS) furnished **32** in 97% yield. On the other hand, bromination of **13** with NBS delivered **33** in 74% yield. SeO_2_-mediated oxidation of **13** and **16** in AcOH delivered the alcohols **34** and **35** in 31% and 51% yields, respectively.

### Selective 11*β*-HSD1 inhibition in vitro

With diverse tanshinones in hand, inhibition of human and mouse 11*β*-HSD1 enzymatic potency was measured by scintillation proximity assay (SPA) using microsomes containing 11*β*-HSD1 [46, 47]. Results are reported as the average of two or three independent experiments at each concentration. The preliminary evaluations of the derivatives were performed at the concentration of 1.0 μM (Table2). Derivatives with inhibitory ratio more than 50% in preliminary tests were further evaluated to calculate the IC_50_ value, as well as the selectivity ratio, against 11*β*-HSD2 (Table 3).

**Table 2:** Enzymatic assay of the synthesized tanshinone derivatives

Entry	Inhibitory ratio^a^		Entry	Inhibitory ratio^a^	
	*m*-HSD1	*h*-HSD1		*m*-HSD1	*h*-HSD1
**1**	89.6 ± 0.6%	76.6 ± 1.3%	**25**	85.1 ± 0.6%	35.5 ± 2.8%
**2**	89.9 ± 3.7%	91.3 ± 0.1%	**27a**	25.9 ± 0.3%	33.9 ± 1.8%
**9**	8.4% ± 2.3	14.0 ± 1.3%	**27b**	5.6 ± 0.5%	3.8 ± 1.5%
**10**	89.9 ± 0.9%	87.6 ± 2.2%	**27c**	70.6 ± 0.5%	82.5 ± 0.9%
**11**	33.0 ± 2.3%	9.9 ± 1.1%	**27d**	37.4 ± 2.8%	14.5 ± 1.4%
**12**	12.4 ± 1.4%	10.0 ± 2.3%	**27e**	42.9 ± 3.7%	15.1 ± 0.8%
**13**	92.3 ± 2.7%	67.4 ± 1.6%	**28a**	79.2 ± 2.2%	74.3 ± 2.1%
**14**	85.9 ± 1.6%	57.2 ± 2.0%	**28b**	88.5 ± 0.1%	75.3 ± 0.8%
**15**	85.3 ± 0.2%	40.6 ± 1.5%	**29a**	96.6 ± 0.6%	92.1 ± 2.4%
**16**	89.3 ± 1.5%	50.3 ± 2.7%	**29b**	89.5 ± 0.0%	87.8 ± 0.8%
**17**	84.6 ± 1.4%	63.5 ± 3.2%	**29c**	6.7 ± 4.3%	45.2 ± 0.7%
**18a**	89.0 ± 3.5%	31.1 ± 0.2%	**29d**	55.6 ± 1.8%	67.3 ± 3.1%
**18b**	49.4 ± 1.6%	43.2 ± 1.7%	**30**	98.6 ± 0.7%	82.0 ± 2.7%
**19**	86.7 ± 2.1%	63.9 ± 2.7%	**31a**	88.9 ± 3.5%	32.6 ± 5.8%
**20**	81.8 ± 1.5%	38.5 ± 2.2%	**31b**	37.0 ± 3.2%	16.9 ± 3.7%
**26**	19.2 ± 5.3%	12.8 ± 0.8%	**32**	70.1 ± 1.1%	21.4 ± 1.8%
**22**	11.3 ± 0.3%	11.9 ± 1.4%	**33**	87.3 ± 1.3%	70.0 ± 0.4%
**21**	28.6 ± 0.3%	5.1 ± 1.8%	**34**	90.8 ± 1.7%	53.9 ± 1.6%
**22**	40.6 ± 4.4%	23.9 ± 4.2%	**35**	83.6 ± 1.9%	48.9 ± 1.9%
**24**	56.0 ± 2.2%	33.6 ± 0.8%	**GA**^b^	95.6 ± 1.0%	93.3 ± 2.2%

^a^The value was determined at the concentration of 1.0 μM. And the values are averages of three determinations

^b^Glycyrrhetinic acid (GA) was used as a positive control

**Table 3:** IC_50_ value and selectivity ratio

Entry	Mouse (IC_50_)			Human (IC_50_)		
	11β-HSD1 (nM)	11β-HSD2 (µM)	SI^b^	11β-HSD1 (nM)	11β-HSD2 (µM)	SI^b^
**1**	32.2 ± 2.5	> 1000	> 31,055.9	35.3 ± 15.4	135.4 ± 72.5	3838.0
**10**	66.5 ± 5.6	> 10	> 150.3	121.1 ± 0.0	> 10	> 82.5
**13**	59.3 ± 9.5	> 100	> 1689.2	270.0 ± 0.0	2.7 ± 1.0	9.9
**14**	53.9 ± 9.3	> 100	> 1855.3	300.0 ± 0.0	5.9 ± 3.4	19.6
**15**	72.9 ± 9.3	> 100	> 1371.7	–	–	–
**16**	10.1 ± 0.8	> 100	> 9900.9	890.0 ± 100.0	7.3 ± 2.2	8.2
**17**	6.9 ± 0.7	> 100	> 14,492.7	210.0 ± 0.0	5.9 ± 2.3	28.0
**18a**	138.1 ± 10.8	> 100	> 724.1	> 1	–	–
**19**	48.4 ± 6.6	> 10	> 206.6	320.0 ± 0.0	2.7 ± 0.5	8.3
**20**	96.6 ± 16.5	> 10	> 103.6	–	–	–
**25**	95.0 ± 17.8	> 10	> 105.3	> 1	–	–
**27c**	6.9 ± 1.2	> 10	> 1451.4	126.0 ± 0.5	6.1 ± 0.7	48.4
**28a**	79.3 ± 16.2	> 100	> 1261.0	149.3 ± 33.9	4.1 ± 0.7	27.5
**28b**	70.2 ± 13.4	> 100	> 1426.5	99.8 ± 10.5	6.3 ± 0.9	62.9
**29a**	17.1 ± 0.9	> 100	> 5813.9	38.0 ± 0.4	5.6 ± 0.9	147.9
**29b**	65.5 ± 3.7	> 100	> 1526.7	42.6 ± 4.4	6.5 ± 3.6	151.9
**29d**	272.3 ± 60.2	> 100	> 367.2	182.6 ± 32.5	2.5 ± 0.6	13.6
**30**	22.2 ± 0.6	> 100	> 4510.6	57.8 ± 13	–	–
**31a**	56.6 ± 0.7	> 10	> 176.4	> 1000	–	–
**32**	328.0 ± 20.2	> 10	> 30.5	> 1000	–	–
**33**	39.3 ± 7.6	> 100	> 2551.0	210.0 ± 0.0	1.2 ± 0.5	5.8
**34**	67.2 ± 6.4	> 100	> 1488.1	850.0 ± 200	1.9 ± 0.3	2.2
**35**	21.9 ± 7.9	> 100	> 4575.5	–	–	–
**GA**^a^	7.4 ± 0.7	–	–	8.6 ± 1.4	1.2 ± 0.1	0.14

Values are averages of two determinations

^a^Glycyrrhetinic acid was used as a positive control

^b^SI: selectivity index, SI = [IC_50_(HSD2)/IC_50_(HSD1)]. The average IC_50_ was used for the calculations

Based on the biological results shown in Tables 1, 2 and 3, the SARs of tanshinones were then discussed accordingly. For A ring, the cyclohexene moiety was superior to the aromatic one in term of the potency against both human and mouse 11*β*-HSD1 (i.e. **1** vs **6**, **2** vs **5**). Installation of alkene moiety at C1 and C2 exerted minor impacts on the activity (i.e. **13** vs **1**). In contrast, polar groups at C1 and C2 significantly diminished inhibitory activity against human 11*β*-HSD1 but not mouse 11*β*-HSD1 (i.e., **18a–18b**, **19**, **20** vs **1**). In addition, polar substituents at C3 and C4 (i.e., **3**, **4**, **14**, **15–16**) reduced the potency and selectivity against both human and mouse 11*β*-HSD1 relative to the lead compounds. Exceptionally, C3 ketone substituent exerted beneficial effects on the potency (i.e., **17** vs **1**). Taken together, the hydrophilic substituent and proper flexibility of A-ring seems to be optimal for the potency, which is particularly true for human 11*β*-HSD1. Notably, results from scaffold hopping design by removing A ring revealed that A-ring seems to be not necessary for both activities and selectivity (i.e., **10** vs **1**).

Interestingly, disrupting of *ortho*-quinone moiety led to a complete loss or a dramatic drop of activity against both human and mouse 11*β*-HSD1 (i.e., **7–8** vs **5**, **21**–**22** vs **2**, **26** vs **1**), suggesting its critical role for the protein binding. Scaffold hoping results also supported this point (i.e.,** 9** vs **6**, **12** vs **11**).

**Fig. 3:**
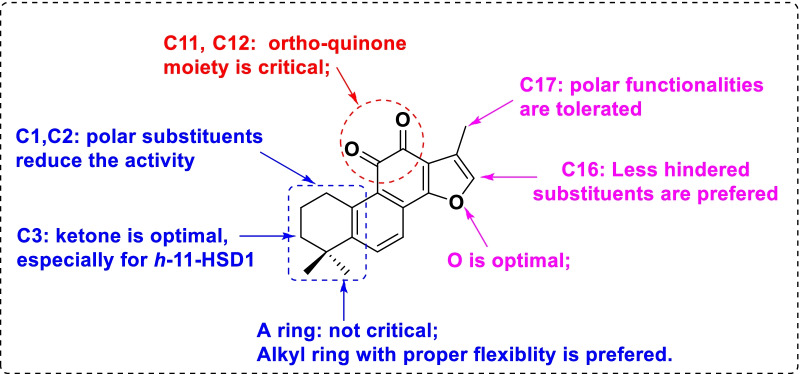
Summary of SARs for selective inhibition against 11*β*-HSD1

It’s obvious that the D-ring opening derivatives exhibited significantly decreased potency against human 11*β*-HSD1, whereas the activities against mouse 11*β*-HSD1 retains for those preserving *ortho*-quinone moiety (i.e., **25** vs **2**), but not for those with *para*-quinone moiety (i.e., **22**). Surprisingly, the bioisosteres (i.e., **24** vs **1**) exerted significantly reduced activities against both human and mouse 11*β*-HSD1. These results indicated that subtle changes in D-ring can dramatically affect the activity and selectivity against both human and mouse 11*β*-HSD1, which is particularly true for human 11*β*-HSD1. Variations at C16 also demonstrated interesting profiles. Derivatives containing C16 pyridinyl substituents (i.e., **27c**), but not phenyl or quinolinyl ones (i.e., **27a-27e**), produced comparable potency and selectivity to that of the parent compound **1**. Moreover, derivatives with C16 hydroxymethyl or aminomethyl moieties (i.e., **28a**, **28b** and **29a**) also demonstrated superior activities and excellent selectivity against both human and mouse 11*β*-HSD1. It’s noteworthy that C17 substituent also provided encouraging results. C17 hydroxyl derivative (i.e., **30**) showed excellent activities and selectivity against both human and mouse 11*β*-HSD1, while other C17 derivatives (i.e., **31a** and **31b**) were much inferior. However, those with additional functionalities at D-ring (i.e., **32**) resulted in a decrease in potency. Notably, derivatives **27d**, **28b**, **29a** and **30** with polar groups not only exhibited excellent activities and selectivity, but also were with good druggability, which merit further investigations. Of particular note is that for the majority of the compounds listed in Table 3, their inhibitions against mouse 11*β*-HSD1 are more potent than that of human 11*β*-HSD1, which is in good agreement with the computational studies. Besides, the selectivity over mouse 11*β*-HSD2 is also better than that over human 11*β*-HSD2. Taken together, the SARs of tanshinones was summarized and illustrated in Fig. 3.

### Molecular modeling analysis

**Fig. 4:**
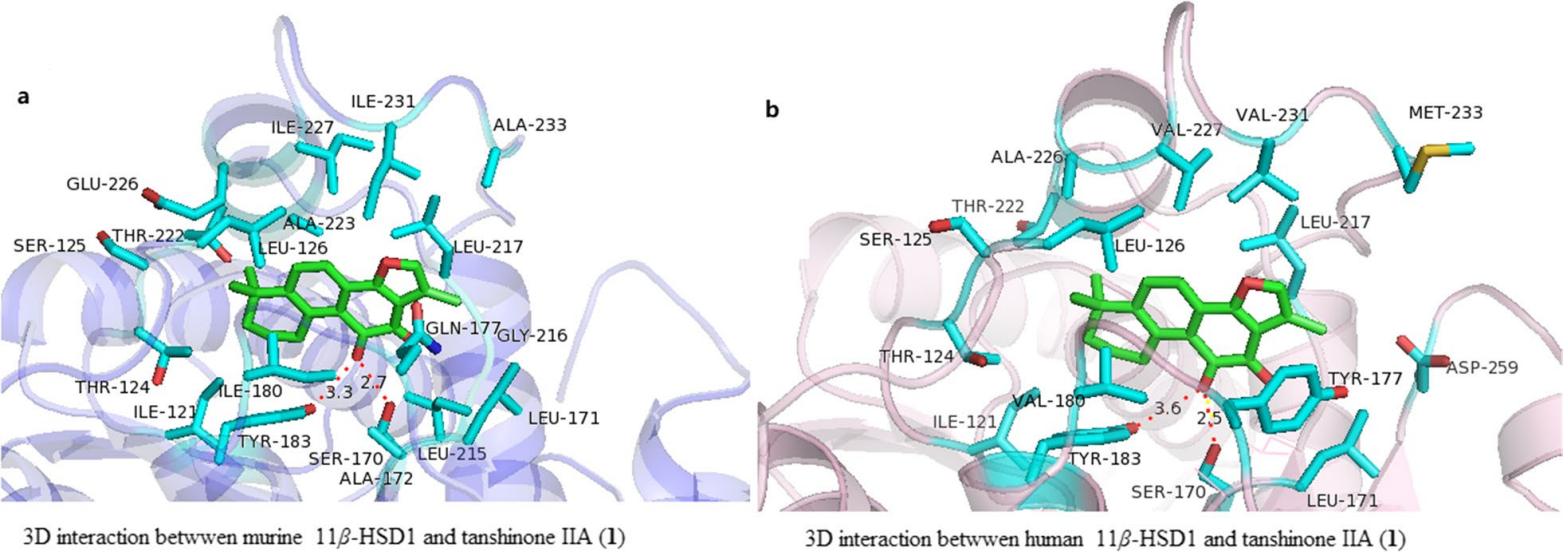
Three-dimensional (3D) interaction schemes between **1** and mouse/human 11*β*-HSD1. The figures were prepared using PyMOL (http://pymol.sourceforge.net/). The ligands and the residues near the pocket are shown as sticks (receptor carbon in cyan and ligand carbon in green). **a** Belongs to mouse 11*β*-HSD1 and **b** belongs to human 11*β*-HSD1. Hydrogen bonds are shown as dotted lines in 3D views

To understand the structural basis for the binding affinity of the inhibitor for 11*β*-HSD1, we scrutinized the binding conformation of **1** by means of` molecular docking using PyMOL. Sequence alignment of human 11*β*-HSD1 with mouse 11*β*-HSD1 is shown in Figure S2. Critical different residues of the binding pocket for human and mouse 11*β*-HSD1 are illustrated in Figure S3. As shown in Fig. 4, the predicted 3D docking conformation of **1** in the mouse and human 11*β*-HSD1 binding site. The top-ranking docking score for **1** with mouse 11*β*-HSD1 is − 8.20 kcal/mol, and − 7.92 kcal/mol with human 11*β*-HSD1. This result shows that the binding affinity of **1** with mouse 11*β*-HSD1 is a little bit higher to human 11*β*-HSD1, which was also in good consistent with the experimental results, i.e., the IC_50_ value (43.89 nM) against human 11*β*-HSD1 is a little bigger than the IC_50_ value (13.29 nM) against mouse 11*β*-HSD1. There are two hydrogen bonds in the both systems which are formed between the C11-ketone in **1** and Tyr183 and Ser170, indicating C11-ketone as the key pharmacophore of tanshinone-based 11*β*-HSD1 inhibitors. This notion was further validated by the experimental results that interrupting the C-ring *ortho*-quinone moiety led to a dramatic drop of activities.

### Cellular assay 11*β*-HSD1 inhibition in vitro

11*β*-HSD1 enzyme inhibition in adipose tissue is believed to be important for achieving efficacy in vivo [11, 12]. Derivatives with potent enzymatic potency and excellent selectivity profiles (**13**, **14**, **17**, **28a**, **28b** and **30**) were also evaluated their 11*β*-HSD1 inhibition in 3T3-L1 adipocyte. As shown in Table 4, all of them showed moderate to good cellular efficacy. Among them, **1** and **17** exhibited comparable inhibitory activity against 11*β*-HSD1 to that of MK544, with IC_50_ of 2.0 μM and 0.4 μM respectively.

**Table 4:** Cellular assay on 3T3-L1 adipocyte against 11*β*-HSD1 enzymes

Entry	IC_50_ (µM)	Max effect	Entry	IC_50_ (µM)	Max effect
1	2.0 ± 0.6	104.0 ± 1.0%	**28a**	3.9 ± 0.1	104.0 ± 0.9%
13	7.9 ± 0.0	102.0 ± 0.3%	**28b**	3.1 ± 0.6	100.0 ± 4.0%
14	> 4.0	104.0 ± 1.2%	**30**	4.4 ± 0.1	105.0 ± 0.0%
17	1.4 ± 0.1	90.0 ± 0.4%	MK544^a^	0.4 ± 0.1	98.0 ± 0.7%

Values are averages of three determinations

^a^MK544: a triazole type selective 11*β*-HSD1 inhibitor developed by Merck, which was used as the positive control

### In vivo 11*β*-HSD1 inhibitory activities on *ob*/*ob* mice

**Fig. 5:**
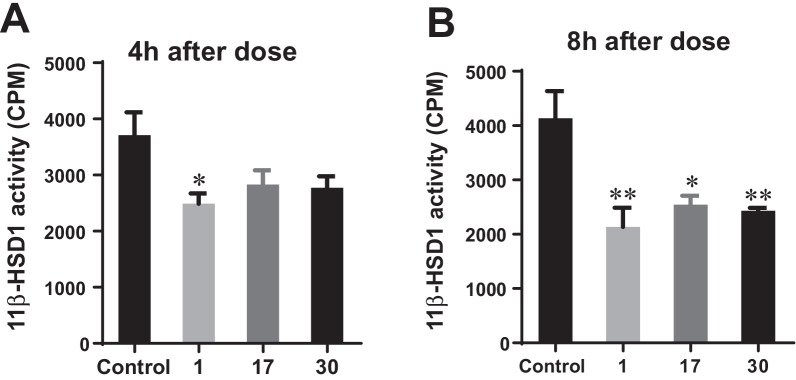
Inhibitory activities of **1**, **17** and **30** against 11*β*-HSD1 in livers of *ob*/*ob* mice. (n = 6, Mean ± SE, *P < 0.05, **P < 0.01 vs control)

After single oral administration of **1**, **17** or **30** at dose of 300 mg/kg to *ob*/*ob* mice, the enzymatic activity of 11*β*-HSD1 in liver was measured. As shown in Fig. 5A, a significant decrease in 11*β*-HSD1 activity, by 32.9%, was observed in the liver of *ob*/*ob* mice at 4 h after treatment with 300 mg/kg of **1**. As shown in Fig. 5B, the 11*β*-HSD1 activity were even more significantly decreased by 48.4%, 38.5% and 41.2% respectively upon **1**, **17** or **30-**administration after 8 h treatment, albeit that there is no significant difference between **1** and **17** or **30**. These results revealed that **1**, **17** and **30** exert potent and selective 11*β*-HSD1 inhibition both in vitro and in vivo.

## Discussions and conclusion

TCM-derived natural products with unique skeletons and bioactivities remain a precious reservior for drug leads and candidates, thereby constituting a hotspot in the field of drug discovery. Tanshinone-based scaffold diversity was intensively investigated by incorporations of a series of novel “privileged” scaffolds, which significantly extended the chemical spaces around tanshinones. Furthermore, the general trends involving SARs of tanshinones were fully discerned from this focused diversity-oriented library and biological results. This work also led to the identification of a series of highly potent and selective 11*β*-HSD1 inhibitors. Among them, compounds **1**, **17** and **30** were quite remarkable both in vitro and in vivo. Based on the biological results, systematic SARs were further investigated, which was also partially rationalized by a molecular docking model of **1** docked to the 11*β*-HSD1 based on the X-ray crystal structure of CBX bound to 11*β*-HSD1. This study not only provides a series of novel selective 11*β*-HSD1 inhibitors with promising therapeutic potentials in metabolic syndromes, but also expands the boundaries of the biological spaces of TCM-derived tanshinones. Further explorations of tanshinones in drug development are underway.

## Experimental section

### Chemistry

Procedures for the synthesis of the compounds mentioned in this article and their characterization data, as well as the spectra copies, are described in Supplementary Information.

### Biological assay

#### 11*β*-HSD1 and 11*β*-HSD2 inhibition assay

The SPA was used to screen for inhibitors of 11*β*-HSDs [48], with the microsome fractions prepared from the HEK-293 cells stably transfected with either human or mouse 11*β*-HSD1 or 11*β*-HSD2 as the enzyme source. Briefly, different concentrations of compounds were added to 96-well microtitre plates, followed by the addition of 80 μL of 50 mM HEPES buffer, *p*H 7.4 containing 25 nM [1,2-(n)^3^H]-cortisone and 1.25 mM NADPH (for 11*β*-HSD1 assay) or 12.5 nM [1,2,6,7-(n)^3^H]-cortisol and 0.625 mM NAD^+^(for 11*β*-HSD2 assay). Reactions were initiated by the addition of 11*β*-HSD1 or 11*β*-HSD2, enzyme. Procedure for the synthesis as microsome fractions from HEK293 cells in a final concentration of 320 μg/mL for human and mouse 11*β*-HSD1, 160 μg/mL for human 11*β*-HSD2 and 320 μg/mL for mouse 11*β*-HSD2, respectively. After a 60 min incubation at 37 °C, the reaction was stopped by the addition of 35 μL of 10 mg/mL protein A-coated yttrium silicate beads suspended in SuperBlock Blocking Buffer with 3 μg/mL of murine monoclonal cortisol antibody and 314 μM glycyrrhetinic acid. The plates were incubated under plastic film on an orbital shaker for 120 min at room temperature before counting. The amount of [^3^H]-cortisol generated in 11*β*-HSD1 enzyme reaction or remaining from the 11*β*-HSD2 enzyme reaction was captured by the beads and determined in a microplate liquid scintillation counter. The % inhibition was calculated relative to a non-inhibited control. Data were obtained from two or three independent experiments. IC_50_ values were calculated from concentration–response curves by a non-linear regression analysis using Prism Version 6.

#### 11*β*-HSD1 activity assay in 3T3-L1 adipocytes

The reductase activity of 11*β*-HSD1 in intact 3T3-L1 adipocytes was determined by measuring the rate of conversion of cortisone to cortisol. To explore the effect of compounds on 11*β*-HSD1 reductase activity, 3T3-L1 adipocytes were incubated for 1 h at 37 ºC in serum-free DMEM containing 6.25 nmol/L [1,2-(N)^3^H]-cortisone and different concentrations of compounds, according to experimental design. 0.1% DMSO was set as the vehicle control and MK544 was set as the positive control. At the end of the incubation, 80 μL of medium was pipetted into a transparent bottom 96-well plate, and 35 μL of SuperBlock Blocking Buffer containing 10 g/L of protein A-coated yttrium silicate beads and 3 mg/L of anti-cortisol antibodies was added. The mixtures were shaken in the dark for 2 h and then used for liquid scintillation readings. The inhibition effect was calculated by the following equation:

$${\text{Inhibition}}\%= \left[ {{\text{Data}}_{{({\text{compound}})}} - {\text{Data}}_{{({\text{DMSO Vehicle}})}} } \right]/\left[ {{\text{ Data}}_{{({\text{MK544 1}}0\mu {\text{M}})}} -{\text{ Data}}_{{({\text{DMSO Vehicle}})}} } \right] \times 100\% .$$

#### Measurement of 11*β*-HSD1 activity in liver of *ob/ob* mice

B6.V-Lepob/Lepob mice (Jackson Laboratory, Bar Harbor, ME, USA) were bred at the Shanghai Institute of Materia Medica, Chinese Academy of Sciences. All mice were housed in a specific, pathogen-free class laboratory and were maintained at a controlled room temperature (22–24 °C) under a 12 h light–dark cycle with free access to water and food. The animal experiments were conducted in accordance with the guides by the Institutional Animal Care and Utilization Committee (IACUC) of Shanghai Institute of Materia Medica (SIMM), Chinese Academy of Sciences (CAS). The protocol was approved by the IACUC, SIMM, CAS with accreditation number of 2017-08-LY-67. All animal experiment complied with the ARRIVE guidelines for the care and use of laboratory animals.

Ob/ob mice (10 weeks’ old) were given a single dose of **1**, **17**, **30** (300 mg/kg) or vehicle (0.5% carboxymethyl cellulose, CMC), p.o. After 4 h and 8 h, animals were euthanized with an i.p injection of sodium pentobarbital (60 mg/kg) and the livers were isolated and stored as described above. The liver tissue was homogenized (1 mg/mL) in cold homogenization buffer, and 30 mg of liver homogenates was used to analyze the 11β-HSD1 activity by SPA, as previously described.

### Computational methods

Tanshinone IIA (**1**) was prepared (generating stereoisomers and valid single 3D conformers) by means of the Ligand Procedure for the synthesis module in Maestro. The crystal structures of mouse and human 11*β*-HSD1 was retrieved from the Protein Data Bank (PDB entry: 1Y5R and 2IRW). All crystallographic water molecules were removed from the coordinate set. Cofactor NDP complexed in the crystal structure was reserved. Glide was used for docking. In the docking process, the standard docking score was used to rank the docking conformations. All the parameters were set as the default values. The grid-enclosing box was centered on the centroid of cocomplexed A in 11*β*-HSD1 and defined so as to enclose residues located within 14.0 Å around the A binding site and default van der Waal scaling was used (1.0 for the receptor and 0.8 for the ligand). Validation of the adopted docking procedure was conducted via re-docking of the co-crystallized ligands (corticosterone for 1Y5R and adamantane ether for 2IRW) and calculating RMSD between docked and re-docked ligands. The RMSD values between the top-scoring ligand orientation and the crystal ligand orientation 1Y5R and 2IRW were 0.33 and 0.65 Å, respectively, which were less than 2.0 Å (see Figure S1). The binding poses of tanshinone IIA (1) were modeled by Glide [49, 50] (Schrödinger 2010, Inc.) in SP mode with the same parameter settings.

## Supplementary Information

Below is the link to the electronic supplementary material.

Supplementary file1 (DOC 4526 KB)
