# Supplementary material for: Development and structure–activity relationships of tanshinones as selective 11β-hydroxysteroid dehydrogenase 1 inhibitors
Source: Nat Prod Bioprospect. 2022 Sep 22;12(1):36. doi: 10.1007/s13659-022-00358-9 (PMC9492458; doi:10.1007/s13659-022-00358-9)
Supplement: Supplementary file 1 — Supplementary file1 (DOC 4526 KB) [file 13659_2022_358_MOESM1_ESM.doc]

**Supporting Information**

**Development and structure-activity relationships of tanshinones as selective 11*β*-hydroxysteroid dehydrogenase 1 inhibitors**

Xu Deng,*^b#^* Su-Ling Huang,*^c#^* Jian Ren,*^a^* Zheng-Hong Pan,*^a,d^* Yu Shen,*^c^* Hao-Feng Zhou, *^a^* Zhi-Li Zuo,*^a^*[[1]](#footnote-2)* Ying Leng,*^c^*[[2]](#footnote-3)* and Qin-Shi Zhao*^a^*[[3]](#footnote-4)*

*^a^State Key Laboratory of Phytochemistry and Plant Resources in West China, Kunming Institute of Botany, Chinese Academy of Sciences, Kunming 650204, PR China*

*^b^ Xiangya School of Pharmaceutical Sciences, Central South University, Changsha, 410013, PR China*

*^c^Shanghai Institute of Materia Medica, Chinese Academy of Sciences, Shanghai 201203, PR China*

*^d^Guangxi Key Laboratory of Functional Phytochemicals Research and Utilization, Guangxi Institute of Botany, Chinese Academy of Sciences, Guilin 541006, China*

**List of Supporting Information**

**General information…………………………………………..…………………..S2**

**Isolation of tanshinones 1-8 from *Salvia trijuga*………..………….….……..S2-S5**

**Typical procedure for synthesis of 13-35 and their spectra data.....…….…S7-S26**

**Computational results…………………………………………….…….……S27-S28**

**NMR spectra copy for compounds 9-35……………………………….……S29-S64**

**References………………………....………………..…………………..…….S65-S66**

**General Information:**

Thin layer chromatographies were carried out on Merck silica plates (0.25 mm layer thickness). Flash chromatography was performed with 300–400 mesh silica gels. Reported yields were for isolated, spectroscopically pure compounds. ^1^H and ^13^C NMR experiments were performed on a Bruker AM-300, AM-400 and DRX-500 NMR spectrometer at ambient temperature. The residual solvent protons (^1^H) or the solvent carbons (^13^C) were used as internal standards. ^1^H-NMR data are presented as follows: chemical shift in ppm downfield from tetramethylsilane (multiplicity, coupling constant, integration). The following abbreviations are used in reporting NMR data: s, singlet; br s, broad singlet; d, doublet; t, triplet; q, quartet; qt, quartet of triplets; dd, doublet of doublets; dt, doublet of triplets; m, multiplet. EIMS and HREIMS were taken on a VG Auto Spec-3000 or on a Finnigan MAT 90 instrument.

**Procedure for the isolation and characterization data of 1-8.**

The air-dried and powdered whole plant of *Salvia trijuga* (19.5 kg) was extracted with acetone (3×50 L), each for 48 h, at room temperature, and concentrated in vacuum. The crude extract was partitioned between H_2_O and EtOAc. The EtOAc portion (900 g) was chromatographed on a silica gel column (100-200 mesh), eluting with a gradient of petroleum ether-acetone (1:0, 9:1, 8:2, 7:3, 3:2, and 0:1) to afford five fractions (A-E). Tanshinone IIA (**1**, 10 g) was precipitation from fraction B. The filtrate was subjected to silica gel column by eluting with petrol ether-EtOAc or petrol ether-CHCl_3_-EtOAc, which was subsequently purified with Sephadex LH-20 (CHCl_3_: MeOH = 1:1) to give **3** (5.0 mg), **5** (30.0 mg), **6** (5.0 mg), **7** and **8** (35.0 mg). And fraction C was recrystallized to give cryptotanshinone **2** (8.0 g). The mother solution was further chromatographed with RP-18 column (MeOH: H_2_O = 5:5, 6:4, 10:0) to provide three subfractions. Fraction 1 was purified with silica gel column and then chromatographed over Sephadex LH-20 eluting with CHCl_3_: MeOH (1:1), to afford **4** (0.32 g).

***6,7,8,9-tetrahydro-1,6,6-trimethyl-phenanthro[1,2-b]furan-10,11- dione*** *(****Tanshinone IIA, 1****).* The spectral data are identical to those of a previous report **.** red powder; ^1^H NMR (400 MHz, CDCl_3_): *δ*_H_ 1.82 (2H, m, H-1), 1.68 (2H, m, H-2), 3.21 (2H, m, H-3), 7.66 (1H, d, *J* = 6.4 Hz,H-6), 7.56 (1H, d, *J* = 6.4 Hz, H-7), 7.29 (1H, s, H-16), 2.24 (3H, s, Me-17), 1.29 (6H, s, Me-18 and Me-19); ^13^C NMR (100 MHz, CDCl_3_): *δ*_C_ 183.3 (C), 175.9 (C), 161.9 (C), 150.4 (C), 144.7 (C), 141.5 (CH), 133.6 (CH), 127.7 (C), 126.7 (C), 121.4 (C), 120.5 (CH), 120.5 (C), 37.7 (CH_2_), 34.9 (C), 32.1 (CH_3_), 29.9 (CH_2_), 19.4 (CH_2_), 9.0 (CH_3_).

***(3R)-1,2,6,7,8,9-hexahydro-1,6,6-trimethyl-phenanthro[1,2-b] furan -10,11-dione (Cryptotanshinone, 2)*:** The spectral data are identical to those of a previous report **.** red solid; ^1^H NMR (400 MHz, CDCl_3_): *δ*_H_ 3.18 (2H, m, H-1), 1.76 (2H, m, H-2), 1.62 (2H, m, H-3), 7.60 (1H, d, *J* = 8.0 Hz, H-6), 7.45 (1H, d, *J* = 8.0 Hz, H-7), 3.55 (1H, m, H-15), 4.86 (1H, t, *J* = 9.2 Hz, H-16a), 4.32 (1H, dd, *J* = 6.0, 9.2 Hz, H-16b), 1.18 (3H, s, Me-17), 1.33 (3H, s, Me-18), 1.31 (3H, s, Me-19); ^13^C NMR (100 MHz, CDCl_3_): *δ*_C_ 184.2 (C), 175.6 (C), 170.8 (C), 152.3 (C), 143.7 (C), 132.6 (CH), 128.3 (CH), 126.8 (C), 122.5 (CH), 118.4 (C), 81.4 (CH_2_), 37.7 (CH_2_), 34.8 (C), 34.5 (CH), 31.9 (CH_3_), 31.8 (CH_3_), 29.6 (CH_2_), 19.0 (t, CH_2_), 18.8 (CH_3_).

**(6*R*)-*6,7,8,9-Tetrahydro-1,6-dimethyl-6-hydroxy- phenanthro[1,2-b] furan-10,11-dione* (Tanshinol B, 3):** The spectral data are identical to those of a previous report. deep red solid; ^1^H NMR (400 MHz, C_5_D_5_N): *δ*_H_ 2.98-3.10 (2H, m, H-1), 1.63-1.88 (2H, m, H-2), 1.76 (2H, m, H-3), 7.92 (1H, d, *J* = 6.4 Hz, H-6), 7.55 (1H, d, *J* = 6.4 Hz, H-7), 7.63 (1H, *br.* s, H-16), 2.14 (3H, s, Me-17), 1.39 (6H, s, Me-18 and Me-19), 5.14 (1H, br.s, OH); ^13^C NMR (125 MHz, C_5_D_5_N): δ_C_ 29.5 (t, C-1), 20.5 (t, C-2), 38.8 (t, C-3), 69.8 (s, C-4), 149.3 (s, C-5), 133.9 (d, C-6), 120.6 (d, C-7), 128.6 (s, C-8), 126.9 (s, C-9), 143.2 (s, C-10), 183.6 (s, C-11), 176.1 (s, C-12), 121.1 (s, C-13), 161.4 (s, C-14), 120.6 (s, C-15), 142.2 (d, C-16), 8.8 (q, C-17), 31.9 (q, C-18).

**(6*S*)-*6,7,8,9-Tetrahydro-1,6-dimethyl-6-hydroxymethyl- phenanthro[1,2-b]furan-10,11-dione* (Tanshinone IIB, 4):** The spectral data are identical to those of a previous report. red solid; ^1^H-NMR (600 MHz, CDCl_3_) δ_H_ 7.62 (1H, d, J = 8.25 Hz), 7.51 (1H, d, J = 8.25 Hz), 7.19 (1H, d, J = 1.37 Hz), 3.75 (1H, d, J = 11.00 Hz), 3.58 (1H, d, J = 11.00 Hz), 3.17 (2H, m), 2.22 (3H, s), 1.95 (1H, m), 1.85 (1H, m), 1.74 (1H, m), 1.54 (1H, m), 1.27 (3H, s); ^13^C NMR (150 MHz, CDCl_3_): δ_C_ 183.7 (C), 175.7 (C), 161.4 (C), 146.4 (C), 146.1 (C), 141.7 (CH), 133.9 (CH), 128.7 (C), 126.7 (C), 121.0 (C), 120.4 (CH), 120.2 (C), 71.8 (CH2), 40.4 (C), 32.5 (CH_2_), 30.3 (CH_2_), 27.0 (CH_3_), 19.2 (CH_2_), 8.9 (CH_3_).

**(1*R*)-*1,2-Dihydro-1,6-dimethyl-phenanthro[1,2-b]-10,11-dione* (Dihydrotanshinone I, 5):** The spectral data are identical to those of a previous report **.** red powder; ^1^H NMR (400 MHz, CDCl_3_): *δ*_H_ 9.28 (1H, d, *J* = 8.8 Hz), 7.57 (1H, t, *J* = 8.8 Hz), 7.38 (1H, d, *J* = 8.8 Hz), 8.29 (1H, d, *J* = 8.8 Hz), 7.74 (1H, d, *J* = 8.8 Hz), 3.63 (1H, m), 4.95 (1H, t, *J* = 9.6 Hz), 4.42 (1H, dd, *J* = 6.4, 9.6 Hz), 1.39 (3H, d, *J* = 7.0 Hz), 2.69 (3H, s); ^13^C NMR (100 MHz, CDCl_3_): *δ*_C_ 184.4 (C), 175.8 (C), 170.5 (C), 135.0 (C), 134.8 (C), 132.2 (C), 131.9 (CH), 130.4 (CH), 128.9 (CH), 128.3 (C), 126.2 (C), 125.1 (CH), 120.3 (CH), 118.4 (C), 81.6 (CH_2_), 34.8 (CH), 19.8 (CH_3_), 18.8 (CH_3_).

***1,6-dimethyl-phenanthro[1,2-b]furan-10,11-dione* (Tanshinone I, 6):** The spectral data are identical to those of a previous report **.** red powder; ^1^H NMR (400 MHz, CDCl_3_): *δ*_H_ 9.25 (1H, d, *J* = 8.8 Hz), 7.55 (1H, dd, *J* = 8.8 Hz), 7.35 (1H, d, *J* = 8.8 Hz), 8.30 (1H, d, *J* = 8.8 Hz), 7.82 (1H, d, *J* = 8.8 Hz), 7.30 (1H, s), 2.29 (3H, s), 2.69 (3H, s); ^13^C NMR (100 MHz, CDCl_3_): *δ*_C_ 183.6 (C), 175.7 (C), 161.2 (C), 142.1 (CH), 135.2 (C), 133.7 (C), 132.9 (CH), 132.8 (C), 130.7 (CH), 129.7 (C), 128.4 (CH), 124.6 (CH), 123.3 (C), 121.8 (C), 120.5 (C), 118.8 (CH), 19.8 (CH_3_), 8.8 (CH_3_).

**(*1R,10R*)-*1,10-Dihydro-10-hydroxy-1,6-dimethyl-10-(2-oxopropyl)- phenanthro[1,2-b]furan-11(2H)-one* (Danshinol C, 7):** The spectral data are identical to those of a previous report. yellow film; ^1^H NMR (400 MHz, CDCl_3_): *δ*_H_ 1.37 (3H, d, *J* = 6.8 Hz), 1.97 (3H, s), 2.71 (3H, s), 3.05 (1H, d, *J* = 13.2 Hz), 3.28 (1H, d, *J* = 13.2 Hz), 3.61 (1H, m), 4.41 (1H, dd, *J* = 9.3, 4.4 Hz), 4.92 (1H, dd, *J* = 9.3, 4.4 Hz), 7.42 (1H, d, *J* = 7.4 Hz), 7.50 (1H, dd, *J* = 8.8, 7.4 Hz), 7.77 (1H, d, *J* = 8.8 Hz), 8.08 (1H, d, *J* = 8.8 Hz), 9.00 (1H, d, *J* = 8.8 Hz) ; ^13^C NMR (100 MHz, CDCl_3_): δ_C_ 205.1 (C), 196.1 (C), 171.5 (C), 141.3 (C), 135.2 (C), 134.8 (C), 131.1 (C), 128.5 (CH), 126.7 (CH), 125.7 (CH), 125.5 (CH), 120.0 (CH), 120.4 (C), 113.3 (C), 81.6 (CH_2_), 79.4 (C), 57.2 (CH2), 34.8 (CH), 31.9 (CH_3_), 20.1 (CH_3_), 19.4 (CH_3_).

**(*R*)-*3-Hydroxy-2-(2-hydroxy-1-methylethyl)-8-methyl-1,4- phenanthrenedione* (Danshexinkun A, 8):** The spectral data are identical to those of a previous report. yellow solid; ^1^H NMR (500 MHz, CDCl_3_-CD_3_OD, 1:1): *δ*_H_ 1.32 (3H, d, *J* = 7.0 Hz), 2.73 (3H, s), 3.51 (1H, m), 3.92 (1H, dq, *J* = 10.6, 7.3 and 6.2 Hz), 3.96 (1H, dq, *J* = 10.6, 7.3 and 6.2 Hz), 7.45 (1H, d, *J* = 6.6 Hz), 7.58 (1H, t, *J* = 8.4, 6.6 Hz), 8.21 (1H, d, *J* = 8.7 Hz), 8.40 (1H, d, *J* = 8.7 Hz), 9.40 (1H, d, *J* = 8.4 Hz) ; ^13^C NMR (125 MHz, CDCl_3_-CD_3_OD, 1:1): δ_C_ 184.3 (C), 186.4 (C), 156.1 (C), 135.7 (C), 135.7 (C), 133.8 (C), 132.3 (CH), 130.9 (C), 130.5 (CH), 129.5 (CH), 126.1 (CH), 125.7 (C), 122.7 (CH), 122.4 (C), 65.6 (CH_2_), 33.4 (CH), 19.9 (CH_3_), 15.0 (CH_3_).

***1-Methyl-11H-furo[2,3-d]naphtha[1,2-b]pyran-11-one (9)***: Procedure for the synthesis of **9** follows that reported in the literature . red solid; ^1^H NMR (600 MHz, CDCl_3_) δ_H_ = 8.60 (d, *J* = 8.1 Hz, 1H), 7.88 (d, *J* = 7.7 Hz, 1H), 7.85 (d, *J* = 8.6 Hz, 1H), 7.75 (d, *J* = 8.6 Hz, 1H), 7.69 – 7.59 (m, 2H), 7.44 (d, *J* = 1.2 Hz, 1H), 2.41 (d, *J* = 1.2 Hz, 3H); ^13^C NMR (151 MHz, CDCl_3_) δ_C_ = 158.7 (C), 158.7 (C), 149.3 (C), 141.1 (CH), 134.1 (C), 128.0 (CH), 128.0 (CH), 127.3 (CH), 124.6 (CH), 123.4 (C), 122.5 (CH), 120.4 (C), 117.0 (CH), 110.3 (C), 108.4 (C), 8.6 (CH_3_); HR-EI-MS (*m/z*): calcd. for C_17_H_10_O_3_ [M+H]^+^, 251.0703, found 251.0693.

***3-Methyl-naphtha[1,2-b]furan-4,5-dione (10)***: Procedure for the synthesis of **10** follows that reported in the literature . red solid; ^1^H NMR (400 MHz, CDCl_3_) δ ^1^H NMR (600 MHz, CDCl_3_) δ_H_ = 8.06 (dd, *J* = 7.7, 0.6 Hz, 1H), 7.71 – 7.67 (m, 1H), 7.64 (td, *J* = 7.6, 1.1 Hz, 1H), 7.44 (td, *J* = 7.6, 1.1 Hz, 1H), 7.27 (d, *J* = 1.2 Hz, 1H), 2.29 (d, *J* = 1.2 Hz, 3H); ^13^C NMR (151 MHz, CDCl_3_) δ_C_ = 180.7 (C), 175.5 (C), 160.5 (C), 141.6 (CH), 135.3 (CH), 130.4 (CH), 129.9 (CH), 128.7 (C), 128.6 (C), 122.1 (CH), 121.6 (C), 121.1 (C), 8.8 (CH_3_); HR-EI-MS (*m/z*): calcd. for C_13_H_8_O_3_Na [M+Na]^+^, 235.0366, found 235.0363.

***3-Methyl-4,5-benzofurandione (11)*:** Procedure for the synthesis of **11** follows that reported in the literature . red solid; ^1^H NMR (400 MHz, DMSO) δ_H_ = 7.74 (s, 1H), 7.55 (d, *J* = 10.3 Hz, 1H), 6.16 (d, *J* = 10.3 Hz, 1H), 2.15 (s, 3H); ^13^C NMR (101 MHz, DMSO) δ_C_ = 181.9 (C), 174.7 (C), 159.8 (C), 144.1 (CH), 131.4 (CH), 126.7 (CH), 122.7 (C), 121.7 (C), 9.0 (CH_3_); HR-EI-MS (*m/z*): calcd. for C_9_H_6_O_3_Na [M+Na]^+^, 185.0209, found 185.0209.

***3-Methyl-5-benzofuranol*** *(****12)*:** Procedure for the synthesis of **12** follows that reported in the literature . red solid; ^1^H NMR (600 MHz, CDCl_3_) δ_H_ = 7.37 (d, *J* = 0.9 Hz, 1H), 7.30 (d, *J* = 8.7 Hz, 1H), 6.93 (d, *J* = 2.5 Hz, 1H), 6.79 (dd, *J* = 8.7, 2.5 Hz, 1H), 4.65 (s, 1H), 2.19 (d, *J* = 1.2 Hz, 3H); ^13^C NMR (151 MHz, CDCl_3_) δ_C_ = 151.1 (C), 150.2 (C), 142.4 (CH), 129.9 (C), 115.4 (C), 112.5 (CH), 111.6 (CH), 104.5 (CH), 7.8 (CH_3_); HR-EI-MS (*m/z*): calcd. for C_9_H_8_O_2_ [M]^+^, 148.0524, found 148.0522.

**Typical procedure for the synthesis of 13-35 and their characterization data.**

**Procedure for the synthesis of 13:**

To a solution of tanshinone IIA (**1**, 29.0 mg, 0.1 mmol) in CCl_4_ (5.0 mL) under argon were added NBS (27.0 mg, 0.15 mmol) and benzoyl peroxide (36.0 mg, 0.15 mmol) succesively. The resulting mixture was heated to reflux for 3 hours until no starting material was detected according to TLC. Then a saturated aqueous Na_2_SO_3_ solution was added to quench the reaction. The aqueous phase was extracted with DCM for three times. The combined organic layers were washed with water and saturated aqueous NaCl solution, dried over anhydrous Na_2_SO_4_ and concentrated in vacuum. The crude residue was subjected to flash column chromatography to give **13** (15.0 mg, 53%) as a red solid.

***1,6,6-Trimethyl-6,7-dihydro-phenanthro[1,2-b]-furan-10,11-dione (13)***: The spectral data are identical to those of a previous report. red solid, ^1^H -NMR (400 MHz, CDCl_3_) δ_H_ = 7.86 (d, *J* = 12 Hz, 1H), 7.57 (s, 2H), 7.23 (s, 1H), 6.33 (dt, *J* = 8 Hz, 4 Hz, 1H), 2.28 (m, 2H), 2.27 (s, 3H), 1.30 (s, 6H); ^13^C-NMR (100 MHz, CDCl_3_) δ_C_ = 181.0 (C), 177.2 (C), 148.4 (C), 145.0 (C), 141.2 (CH), 134.0 (CH), 130.2 (CH), 127.9 (C), 124.4 (CH), 124.2 (C), 124.0 (C), 121.5 (CH), 121.0 (C), 37.7 (CH_2_), 34.1 (C), 28.3 (CH_3_), 28.1 (CH_3_),8.7 (CH_3_); HR-EI-MS (*m/z*): calcd. for C_19_H_16_O_3_ [M]^+^, 292.1099, found 292.1098.

**Procedure for the synthesis of 14**

To a solution of **13** (14.6 mg, 0.05 mmol) in 1,4-dioxane (1.0 mL) was added SeO_2_ (22.0 mg, 0.2 mmol). The resulting mixture was heated to reflux for 1.5 hours until no starting material was detected according to TLC. The solvent removed in vacuum. The crude residue was subjected to flash column chromatography to give **14** (10.0 mg, 67%) as light yellow solid.

***1,6,6-Trimethyl-phenanthro[1,2-b]-furan-7,10,11(6H)-trione (14)***: red solid, ^1^H-NMR (500 MHz, CDCl_3_) δ_H_ = 8.92 (d, *J* = 10 Hz, 1H), 7.72 (d, *J* = 10 Hz, 1H), 7.65 (d, *J* = 5 Hz, 1H), 7.23 (s, 1H), 6.34 (d, *J* = 10 Hz, 1H), 2.22 (s, 3H),1.44 (s, 6H); ^13^C-NMR (100 MHz, CDCl_3_) δ_C_ = 201.8 (C), 160.4 (C), 151.2 (C), 150.6 (C), 141.9 (CH), 139.2 (C), 132.3 (CH), 132.2 (CH), 128.5 (CH), 128.2 (C), 125.2 (C), 123.6 (CH), 121.4 (C), 120.5 (C), 47.8 (C), 27.5 (CH_3_), 8.7 (CH_3_); HR-EI-MS (*m/z*): calcd. for C_19_H_14_O_4_ [M]^+^, 306.0892, found 306.0882.

**Procedure for the synthesis of 15:** To a solution of **14** (20 mg, 0.05 mmol) in MeOH/HCOOH (0.8 mL/0.2 mL) was added Pd/C (10% wt, 2.0 mg) in one portion. The mixture was stirred at room temperature under H_2_ atomosphere for 3 hours until no starting material was detected according to TLC. The suspension was filtered and the solvent removed in vacuum. The crude residue was subjected to flash column chromatography to give **15** (8.5 mg, 42%) as red solid.

***1,6,6-Trimethyl-6,7,8,9-tetrahydro-7-hydroxy-phenanthro [1,2-b]-furan-10,11-dione (15)***: The spectral data are identical to those of a previous report . red solid; ^1^H-NMR (400 MHz, CDCl_3_) δ_H_ = 7.66 (d, *J* = 7.5 Hz, 1H), 7.59 (d, *J* = 7.4 Hz, 1H), 7.23 (s, 1H), 3.78 (d, *J* = 6.3 Hz, 1H), 3.42 (d, *J* = 19.5 Hz, 1H), 3.34-3.19 (m, 1H), 2.27 (s, 3H), 2.04 (m, 1H), 1.95 (m, 1H), 1.35 (s, 3H), 1.34 (s, 3H); ^13^C-NMR (100 MHz, CDCl_3_) δ_C_ = 182.4 (C), 174.4 (C), 160.4 (C), 147.7 (C), 141.7 (CH), 140.4 (C), 132.7 (CH), 126.8 (C), 124.9 (C), 120.1 (CH), 119.7 (C), 119.0 (C), 73.0 (CH), 38.7 (C), 28.2 (CH_3_), 25.3 (CH_2_), 25.1 (CH_2_), 24.1 (CH_3_), 7.7 (CH_3_); HR-ESI-MS (*m/z*): calcd. for C_19_H_18_O_4_Na [M+Na]^+^, 333.1097, found 333.1099.

**Procedure for the synthesis of 16**

To a solution of **15** (15.5 mg, 0.05 mmol) and DMAP (2.5 mg, 0.02 mmol) in DCM (1.5 mL) were added Et_3_N (15 mg, 0.15 mmol) and AcCl (5.8 mg, 0. 075 mmol). The mixture was stirred at room temperature for 4 hours until no starting material was detected according to TLC. Then water was added to dilute the mixture. The aqueous phase was extracted with EtOAc for three times. The combined organic layers were washed with water and saturated aqueous NaCl solution, dried over anhydrous Na_2_SO_4_ and concentrated in vacuum. The crude residue was subjected to flash column chromatography to give **16** (15.6 mg, 89%) as a red solid.

***1,6,6-Trimethyl-6,7,8,9-tetrahydro-7-acetoxy-phenanthro [1,2-b]-furan-10,11-dione (16)***: red solid; ^1^H NMR (400 MHz, CDCl_3_) δ_H_ = 7.63 (d, *J* = 8.2 Hz, 1H), 7.59 (d, *J* = 8.2 Hz, 1H), 7.23 (s, 1H), 5.06-4.93 (m, 1H), 3.30 (t, *J* = 6.7 Hz, 2H), 2.26 (s, 3H), 2.05 (s, 3H), 2.04-1.98 (m, 2H), 1.33 (s, 3H), 1.32 (s, 3H); ^13^C NMR (100 MHz, CDCl_3_) δ_C_ =182.4 (C), 174.4 (C), 169.6 (C), 160.3 (C), 146.8 (CH), 141.6 (C), 140.4 (C), 132.3 (CH), 126.9 (C), 124.9 (C), 120.2 (CH), 119.7 (C), 119.1 (C), 74.7 (CH), 37.3 (C), 29.0 (CH_2_), 24.9 (CH_2_), 24.4 (CH_3_), 22.0 (CH_3_), 20.1 (CH_3_), 7.78 (CH_3_); HR-ESI-MS (*m/z*): calcd. for C_21_H_20_O_5_Na [M+Na]^+^, 375.1203, found 375.1205.

**Procedure for the synthesis of 17**

To a solution of **15** (20.0 mg, 0.064 mmol) in DCM (2.0 mL) were added celite and PDC (48.0 mg, 0.13 mmol) succesively. The mixture was stirred at room temperature for 5 hours until no starting material was detected according to TLC. The mixture was filtered and the solvent removed in vacuum. The crude residue was subjected to flash column chromatography (petrol ether : ethyl acetate = 5 : 1) to give **17** (4.0 mg, 20%) as red solid.

***1,6,6-Trimethyl-6,7-dihydro-phenanthro[1,2-b]-furan-7,10,11 (6H)-trione (17)***: red solid; ^1^H NMR (400 MHz, CDCl_3_) δ_H_ = 7.67 (d, *J* = 8.2 Hz, 1H), 7.63 (d, *J* = 8.2 Hz, 1H), 7.27 (d, *J* = 1.0 Hz, 1H), 3.63 (t, *J* = 6.9 Hz, 2H), 2.66 (t, *J* = 7.0 Hz, 2H), 2.28 (d, *J* = 0.7 Hz, 3H), 1.46 (s, 6H); ^13^C NMR (100 MHz, CDCl_3_) δ_C_ = 212.7 (C), 183.8 (C), 175.6 (C), 160.9 (C), 148.1 (C), 142.4 (C), 141.7 (CH), 132.7 (CH), 128.1 (C), 126.5 (C), 121.5 (CH), 121.3 (CH), 120.4 (C), 48.2 (C), 36.3 (CH_2_), 27.1 (CH_3_), 26.2 (CH_2_), 8.8 (CH_3_) ; HR-ESI-MS (*m/z*): calcd. for C_19_H_16_O_4_Na [M+Na]^+^, 331.0941, found 331.0945.

**Procedure for the synthesis of 18a-18b**

To a solution of **13** (202 mg, 0.7 mmol) in THF/H_2_O (4.0 mL/1.0 mL) at 0 ^o^C were added HCOOH (81 μL, 2.1 mmol) and KMnO_4_ (321 mg, 2.1 mmol). The mixture was allowed to warm to the room temperature and stirred for 30 min. Then aqueous Na_2_S_2_O_3_ solution (10%) was added to quench the reaction. The resulting mixture was extracted with EtOAc (20 mL*3). The combined organic layers were washed with saturated aqueous NaCl solution, dried over anhydrous Na_2_SO_4_ and concentrated in vacuum. The crude residue was subjected to flash column chromatography to give **18a** (74.0 mg, 34%), **18b** (92.0 mg, 36%).

***1,6,6-Trimethyl-6,7,8,9-tetrahydro-8,9-dihydroxy-phenanthro [1,2-b]furan-10,11-dione* *(18a)*:** The spectral data are identical to those of a previous report. red solid; ^1^H-NMR (400 MHz, CDCl_3_) δ_H_ = 7.70 (s, 2H), 7.26 (s, 1H), 4.99 (s, 1H), 4.46 (d, *J* = 4.1 Hz, 1H), 3.91 (d, *J* = 3.0 Hz, 1H), 2.26 (s, 3H), 2.09 (t, *J* = 12 Hz, 1H), 1.76 (dd, *J* = 12, 4 Hz, 1H), 1.39 (s, 3H), 1.32 (s, 3H); ^13^C-NMR (100 MHz, CDCl_3_) δ_C_ = 185.4 (C), 175.4 (C), 160.9 (C), 148.9 (CH), 141.9 (C), 141.6 (C), 134.7 (CH), 128.7 (C), 128.1 (C), 122.8 (CH), 121.3 (C), 120.2 (C), 66.1 (CH), 66.0 (CH), 40.0 (CH_2_), 36.7 (C), 31.9 (CH_3_), 31.7 (CH_3_), 8.6 (CH_3_); HR-EI-MS (*m/z*): calcd. for C_19_H_18_O_5_ [M]^+^, 326.1154, found 326.1147

***1,6,6-Trimethyl-7,8-dihydro-8-hydroxy-phenanthro[1,2-b] furan-9,10,11(6H)-trione* *(18b)*:** The spectral data are identical to those of a previous report. yellow solid; ^1^H NMR (400 MHz, CDCl_3_) δ_H_ = 7.78 (d, *J* = 8.3 Hz, 1H), 7.60 (d, *J* = 8.3 Hz, 1H), 7.28 (d, *J* = 1.0 Hz, 1H), 4.96-4.88 (m, 1H), 3.68 (d, *J* = 2.7 Hz, 1H), 2.42 (dd, *J* = 13.6, 6.8 Hz, 1H), 2.27 (d, *J* = 0.7 Hz, 3H), 1.94 (dd, *J* = 13.5, 10.7 Hz, 1H), 1.47 (s, 3H), 1.37 (s, 3H); ^13^C NMR (100 MHz, CDCl_3_) δ_C_ = 200.6 (C), 183.3 (C), 177.4 (C), 159.5 (C), 153.7 (C), 142.0 (CH), 135.2 (C), 131.8 (CH), 131.0 (C), 128.7 (C), 125.5 (CH), 121.5 (C), 121.1 (C), 71.1 (CH), 47.6 (CH_2_), 35.5 (C), 30.8 (CH_3_), 30.7 (CH_3_), 8.7 (CH_3_).

**Procedure for the synthesis of 19**

To a solution of **18a** (53.0 mg, 0.16 mmol) in DCM (3.0 mL) at 0 ^o^C under argon were added BF_3_·Et_2_O (30 μL, 0.24 mmol) and *^n^*Bu_3_SnH (129 μL, 0.48 mmol) succesively. The mixture was stirred at room temperature for 3 hours until no starting material was detected according to TLC. The mixture was quenched with saturated aqueous NaHCO_3_ solution and was extrated with DCM (10 mL*3). The combined organic layers were washed with saturated NaCl solution, dried over anhydrous Na_2_SO_4_, and concentrated in vacuum. The crude residue was subjected to flash column chromatography (petrol ether : ethyl acetate : DCM = 2 : 1 : 1) to give **19** (44 mg, 87%) as the red solid.

***1,6,6-Trimethyl-6,7,8,9-tetrahydro-8-hydroxy-phenanthro[1,2-b] furan-7,10,11 (6H)-trione (19)*:** red solid; ^1^H NMR (400 MHz, CDCl_3_) δ_H_ = 7.63 (d, *J* = 8.2 Hz, 1H), 7.59 (d, *J* = 8.2 Hz, 1H), 7.24 (s, 1H), 4.15-4.05 (m, 1H), 3.81-3.69 (m, 1H), 2.98 (dd, *J* = 18.4, 9.7 Hz, 1H), 2.27 (s, 3H), 1.97 (dd, *J* = 10.0, 2.5 Hz, 1H), 1.74-1.66 (m, 1H), 1.39 (s, 3H), 1.33 (s, 3H); ^13^C NMR (100 MHz, CDCl_3_) δ_C_ = 183.5 (C), 175.5 (C), 161.3 (C), 148.7 (C), 141.4 (CH), 141.0 (C), 133.1 (CH), 128.0 (C), 126.5 (C), 121.2 (C), 120.8 (CH), 120.1 (C), 64.8 (CH), 46.1 (CH_2_), 39.2 (C), 36.3 (CH_2_), 32.5 (CH_3_), 32.3 (CH_3_), 8.8 (CH_3_) ; HR-ESI-MS (*m/z*): calcd. for C_19_H_18_O_4_Na [M+Na]^+^, 333.1097, found 333.1099.

**Procedure for the synthesis of 20**

To a solution of oxalyl choloride (12.0 μL, 0.14 mmol) in DCM (1.0 mL) at -78 ^o^C under argon was added a solution of DMSO (16 μL, 0.23 mmol) in DCM (1.0 mL). The mixture was stirred at -78 ^o^C for 10 min. Then a solution of **18b** (15.0 mg, 0.046 mmol) in DCM (2.0 mL) was added to the mixture. The resulting mixture was stirred at -78 ^o^C for 40 min. Then TEA (64 μL, 0.46 mmol) was added and stirred for another 30 min. Upon the completion of the reaction, the mixture was quenched with ice water and was extrated with DCM (15 mL*3). The combined organic layers were washed with saturated aqueous NaCl solution, dried over anhydrous Na_2_SO_4_, and concentrated in vacuum. The crude residue was subjected to flash column chromatography (petrol ether : ethyl acetate : Dicholoromethane = 5 : 1 : 1) to give **20** (12.0 mg, 82%) as a red solid.

***1,6,6-Trimethyl-8-hydroxy-phenanthro[1,2-b]furan-9,10,11(6H) -trione (20)*:** yellow solid; ^1^H NMR (400 MHz, CDCl_3_) δ_H_ = 7.86 (d, *J* = 8.2 Hz, 1H), 7.78 (d, *J* = 8.3 Hz, 1H), 7.28 (s, 1H), 6.31 (s, 1H), 6.13 (s, 1H), 2.27 (s, 3H), 1.53 (s, 6H); ^13^C NMR (100 MHz, CDCl_3_) δ_C_ = 186.5 (C), 179.7 (C), 178.8 (C), 159.4 (CH), 153.7 (C), 145.4 (C), 142.1 (CH), 133.6 (C), 131.9 (CH), 131.4 (C), 129.2 (C), 125.2 (CH), 125.1 (CH), 121.3 (C), 121.0 (C), 37.8 (C), 30.3 (CH_3_), 8.7 (CH_3_); HR-ESI-MS (*m/z*): calcd. for C_19_H_14_O_5_Na [M+Na]^+^, 345.0733, found 345.0738.

**Procedure for the synthesis of 21**

To a solution of cryptotanshinone (**2**, 29.6 mg, 0.1 mmol) in Ac_2_O (5.0 mL) were added Pt_2_O (5.0 mg) and pyridine (0.2 mL). The atomosphere was replaced with H_2_. The resulting mixture was stirred under H_2_ (balloon pressure) at room temperature for 4 hours until no starting material was detected according to TLC. The insoluable was filtered with celite. The filtrate was concentrated in vacuum. The crude residue was subjected to flash column chromatography to give **21** (31.0 mg, 81%) as a colorless film.

***(1R)-1,6,6-trimethyl-1,2,6,7,8,9-hexahydro-10,11-diacetoxy- phenanthro[1,2-b]furan (21)****:* The spectral data are identical to those of a previous report . Colorless film; ^1^H NMR (400 MHz, CDCl_3_) δ_H_ = 7.74 (d, *J* = 8 Hz, 1H), 7.42 (d, *J* = 8 Hz, 1H), 4.86 (t, *J* = 8 Hz, 1H), 4.30 (dd, *J* = 8 Hz, 8 Hz, 1H ), 2.37 (s, 3H), 2.34 (s, 3H), 1.80 (m, 2H), 1.66 (m, 2H), 1.32 (s, 6H); ^13^C NMR (100 MHz, CDCl_3_) δ_C_ = 169.4 (C), 167.9 (C), 154.6 (C), 144.8 (C), 137.8 (C), 132.1 (C), 130.5 (C), 127.0 (C), 125.3 (CH), 119.6 (CH), 118.0 (C), 79.3 (CH_2_), 38.2 (CH), 36.9 (CH_2_), 34.7 (C), 31.7 (CH_3_), 31.6 (CH_3_), 29.5 (CH_2_), 21.0 (CH_3_), 20.4 (CH_3_), 20.0 (CH_2_), 18.8 (CH_3_); HR-EI-MS (*m/z*): calcd. for C_23_H_26_O_5_ [M]^+^, 382.1780, found 382.1788.

**Procedure for the synthesis of 22**

To a solution of cryptotanshinone (**2**, 148 mg, 0.5 mmol) in THF (7.5 mL) was added aqueous NaOH solution (2.5 mL, 2.0 M). The resulting mixture was stirred at room temperature for 6 hours until starting material was fully converted by TLC. Then water was added to dilute the mixture. The aqueous phase was extracted with EtOAc for three times. The combined organic layers were washed with water and saturated aqueous NaCl solution, dried over anhydrous Na_2_SO_4_ and concentrated in vacuum. The crude residue was subjected to flash column chromatography to give **22** (143 mg, 91%) as a light yellow foam.

***7,7-dimethyl-2-hydroxy-3-((S)-1-hydroxy-2-propyl)-7,8,9,10- tetrahydro-1,4-phenanthrenequinone ((+)-Neocryptotanshinone, 22)*:** The spectral data are identical to those of a previous report **.** light yellow foam; ^1^H NMR (500 MHz, CDCl_3_) δ_H_ = 7.98 (d, *J* = 10 Hz, 1H), 7.73 (d, *J* = 10 Hz, 1H), 3.93 (dd, *J* = 10 Hz, 10 Hz, 1H), 3.83 (dd, *J* = 10 Hz, 5 Hz, 1H), 3.44 (dd, *J* = 5 Hz, 5 Hz, 1H), 3.23 (t, *J* = 5 Hz, 2H), 1.81 (m, 2H), 1.66 (m, 2H), 1.30 (s, 6H), 1.26 (d, *J* = 10 Hz, 3H); ^13^C NMR (125 MHz, CDCl_3_) δ_C_ = 185.3 (C), 182.8 (C), 154.0 (C), 152.8 (C), 140.9 (C), 133.4 (CH_2_), 132.5 (C), 126.3 (C), 125.0 (CH), 122.8 (C), 65.4 (CH_2_), 37.6 (CH_2_), 34.7 (C), 32.9 (CH), 31.7 (CH_3_), 29.8 (CH_2_), 19.0 (CH_2_), 14.5 (CH_3_); HR-ESI-MS (*m/z*): calcd. for C_19_H_22_O_4_Na [M+Na]^+^, 337.1415, found 337.1424.

**Procedure for the synthesis of 24.**

**Step 1:** To a solution of **22** (31.0 mg, 0.1 mmol) in DMSO (1.0 mL) was added IBX in two portions (28.0 mg, 0.1 mmol, 30 mins/portion). The mixture was stirred at room temperature for 3 hours until starting material was fully converted by TLC. The mixture was diluted with water and was extracted with EtOAc for three times. The combined organic layers were washed with water and saturated aqueous NaCl solution, dried over anhydrous Na_2_SO_4_ and concentrated in vacuum. The crude residue was subjected to flash column chromatography to give the aldehyde **23** (20.0 mg, 65%) as a red solid.

**Step 2**: To a solution of **23** (16.0 mg, 0.05 mmol) in AcOH (0.5 mL) was added ammonium acetate (38.5 mg, 0.5 mmol) in one portion. The mixture was heated to 60 ^o^C for 1 hour until no starting material was detected according to TLC. The reaction was quenched with water and was extracted with EtOAc for three times. The combined organic layers were washed with water and saturated aqueous NaCl solution, dried over anhydrous Na_2_SO_4_ and concentrated in vacuum. The crude residue was subjected to flash column chromatography to give **24** (11.1 mg, 76%) as a yellow solid.

***6,7,8,9-tetrahydro-1,6,6-trimethyl-3H-naphth[1,2-g]indole- 10,11-dione* *(24)***: yellow solid (11.1 mg from 0.05 mmol starting material, 76%); ^1^H NMR (400 MHz, CDCl_3_) δ_H_ = 9.39 (s, 1H), 8.09 (d, *J* = 8 Hz, 1H), 7.70 (d, *J* = 8 Hz, 1H), 6.82 (s, 1H), 3.37 (dd, *J* = 8 Hz, 4 Hz, 2H), 2.42 (s, 3H), 1.82 (m, 2H), 1.62 (s, 2H), 1.34 (s, 6H); ^13^C NMR (100 MHz, CDCl_3_) δ_C_ = 181.8 (C), 178.8 (C), 152.8 (C), 151.2 (C), 140.7 (C), 134.5 (C), 134.3 (C), 131.9 (CH), 130.0 (C), 125.0 (CH), 123.4 (CH), 121.7 (C), 37.8 (CH_2_), 34.8 (C), 31.9 (CH_3_), 30.0 (CH_2_), 19.4 (CH_2_), 11.0 (CH_3_); HR-EI-MS (*m/z*): calcd. for C_19_H_19_NO_2_ [M]^+^, 293.1416, found 293.1413.

**Procedure for the synthesis of 25**

To a solution of cryptotanshinone (**2**, 29.6 mg, 0.1 mmol) in THF (1.5 mL) was added NH_3_-H_2_O (33%, 1.0 mL). The resulting mixture was stirred at room temperature for 6 hours until starting material was fully converted by TLC. Then water was added to dilute the mixture. The aqueous phase was extracted with EtOAc for three times. The combined organic layers were washed with water and saturated aqueous NaCl solution, dried over anhydrous Na_2_SO_4_ and concentrated in vacuum. The crude residue was subjected to flash column chromatography to give **25** (15.6 mg, 50%) as a red solid.

***7,7-dimethyl-2-amine-3-((S)-1-hydroxy-2-propyl)-7,8,9,10- tetrahydro-1,2-phenanthrenequinone* *(25)*:** The spectral data are identical to those of a previous report. red solid; ^1^H NMR (400 MHz, CD_3_OD) δ_H_ = 7.72 (d, *J* =8 Hz, 1H), 7.69 (d, *J* =8 Hz, 1H), 3.87 (dd, *J* = 4 Hz, 8 Hz, 1H), 3.74 (dd, *J* = 4 Hz, 8 Hz, 1H), 3.27 (s, 2H), 3.14 (t, *J* = 4 Hz, 1H), 3.07 (dd, *J* = 4 Hz, 8 Hz, 2H), 1.71 (d, *J* = 4 Hz, 2H), 1.61 (t, *J* = 8 Hz, 2H), 1.26 (s, 6H), 1.21 (d, *J* = 4 Hz, 3H); ^13^C NMR (100 MHz, CD_3_OD) δ_C_ = 187.0 (C), 177.2 (C), 159.8 (C), 152.1 (C), 142.6 (C), 133.7 (CH), 131.9 (C), 129.9 (C), 122.8 (CH), 114.2 (C), 66.2 (CH_2_), 39.0 (CH_2_), 35.5 (C), 34.2 (CH), 32.1 (CH_3_), 31.1 (CH_2_), 20.3 (CH_2_), 14.5 (CH_3_); HR-EI-MS (*m/z*): calcd. for C_19_H_23_NO_3_ [M]^+^, 313.1673, found 313.1678.

**Procedure for the synthesis of 26**

To a solution of tanshinone IIA (**1**, 29.4 mg, 0.1 mmol) in EtOH (1.5 mL) were added 4-hydroxybenzylaldehyde (30.0 mg, 0.2 mmol) and ammonium acetate (15.0 mg, 0.2 mmol) successively. The resulting mixture was heated to reflux for 4 hours until starting material was fully converted according to TLC. Then water was added upon cooling to the room temperature. The aqueous phase was extracted with EtOAc for three times. The combined organic layers were washed with water and saturated aqueous NaCl solution, dried over anhydrous Na_2_SO_4_ and concentrated in vacuum. The crude residue was subjected to flash column chromatography to give **26** (18.0 mg, 48%) as a yellow solid.

***1,6,6-Trimethyl-11-(4-hydroxyl)-4,8,9,10-tetrahydro-6H-furo [2’,3’ :1,2]-phenanthro[3,4-d]imidazole (26)***: yellow solid; ^1^H NMR (500 MHz, CDCl_3_) δ_H_ = 8.62 (d, *J* = 8 Hz, 2H), 8.52 (d, *J* = 8 Hz, 2H), 7.80 (s, 1H), 7.73 (d, *J* = 8 Hz, 1H), 7.31 (d, *J* = 4 Hz, 1H), 3.04 (s, 2H), 2.67 (s, 3H), 2.02 (s, 2H), 1.76 (s, 2H), 1.40 (s, 6H); ^13^C NMR (100 MHz, CDCl_3_) δ_C_ = 160.1(C), 143.1 (C), 141.3 (CH), 135.9 (CH), 129.5 (CH), 124.3 (CH), 123.9 (CH), 118.9 (C), 118.3 (C), 116.5 (CH), 39.2 (CH_2_), 34.9 (C), 32.3 (CH_3_), 31.7 (CH_2_), 20.5 (CH_2_), 10.2 (CH_3_); HR-EI-MS (*m/z*): calcd. for C_26_H_24_N_2_O_2_ [M]^+^, 396.1838, found 396.1829.

**Typical procedure for the synthesis of 27a-27e (examplified by the synthesis of 31a)**

Step 1: To a solution of tanshinone IIA (**1**, 29.4 mg, 0.1 mmol) in DCM (1.0 mL) at -40 ^o^C were added NIS (33.6 mg, 0.15 mmol) and TFA (11.4 mg, 0.1 mmol). The mixture was stirred at room temperature for 3 hours until starting material was fully converted by TLC. The mixture was quenched with saturated aqueous Na_2_SO_3_ solution and was extracted with EtOAc for three times. The combined organic layers were washed with water and saturated aqueous NaCl solution, dried over anhydrous Na_2_SO_4_ and concentrated in vacuum. The crude residue was subjected to flash column chromatography to give tanshinone-bromide (33.2 mg, 79%) as a deep red solid.

Step 2. To a solution of tanshinone-bromide (21.0 mg, 0.05 mmol) in DMF/H_2_O (1.0 mL/0.2 mL) under argon were added PhB(OH)_2_ (12.1 mg, 0.10 mmol), K_2_CO_3_ (13.8 mg, 0.1 mmol) and Pd(PPh_3_)_4_ (5.0 mg, 0.005 mmol) successively. The resulting mixture was heated to 80 ^o^C and was stirred at this temperature for 3 hours until no starting material was dectected according to TLC. The mixture was cooled to the room temperature and was quenched with saturated aqueous NH_4_Cl solution. The aqueous phase was extracted with EtOAc for three times. The combined organic layers were washed with water and saturated aqueous NaCl solution, dried over anhydrous Na_2_SO_4_ and concentrated in vacuum. The crude residue was subjected to flash column chromatography to give **27a** (18.1 mg, 98%) as a deep red solid.

***6,7,8,9-Tetrahydro-1,6,6-trimethyl-2-phenyl-[3,2-c]furophenanthrene-10,11-dione (27a)***, deep red solid, (18.1 mg was obtained from 0.05 mmol starting material, 98%); ^1^H NMR (400 MHz, CDCl_3_) δ_H_ = 7.63 (d, *J* = 8 Hz, 2H), 7.57 (s, 2H), 7.43, 7.41 (t, *J* = 8 Hz, 2H), 7.31 (t, *J* = 8 Hz, 1H), 3.12 (t, *J* = 8 Hz, 2H), 2.45 (s, 3H), 1.73 (m, 2H), 1.60 (m, 2H), 1.25 (s, 6H); ^13^C NMR (100 MHz, CDCl_3_) δ_C_ = 183.5 (C), 175.9 (C), 159.9 (C), 151.2 (C), 150.1 (C), 144.5 (C), 133.4 (CH), 129.8 (C), 128.7 (CH), 128.2 (CH), 127.2 (C), 125.9 (CH), 120.2 (CH), 116.5 (C), 37.7 (CH_2_), 34.6 (C), 31.8 (CH_3_), 29.8 (C), 29.6 (CH_2_), 19.0 (CH_2_), 10.1 (CH_3_); HR-EI-MS (*m/z*): calcd. for C_25_H_22_O_3_ [M]^+^, 370.1569, found 370.1579.

***6,7,8,9-Tetrahydro-1,6,6-trimethyl-2-(4-fluorophenyl)-[3,2-c]furophenanthrene-10,11-dione (27b)***: deep red solid (16.3 mg from 0.05 mmol starting material, 84%); ^1^H NMR (500 MHz, CDCl_3_) δ_H_ = 7.67 (m, 2H), 7.64 (m, 2H), 7.18 (t, *J* = 10 Hz, 2H), 3.19 (t, *J* = 5 Hz, 2H), 2.50 (s, 3H), 1.79 (m, 2H), 1.66 (m, 2H), 1.32 (s, 6H); ^13^C NMR (125 MHz, CDCl_3_) δ_C_ = 183.5 (C), 175.9 (C), 163.4 (C), 159.9 (C), 150.4 (C), 150.1 (C), 144.6 (C), 133.4 (CH), 127.9 (CH), 127.2(C), 126.5 (C), 126.1 (C), 121.5 (C), 120.2 (CH), 116.2 (C), 116.0 (C), 115.8 (C), 37.8 (CH_2_), 34.7 (C), 31.8 (CH_3_), 29.8 (CH_2_), 19.1 (CH_2_), 10.0 (CH_3_); HR-EI-MS (*m/z*): calcd. for C_25_H_21_O_3_F [M]^+^, 388.1475, found 388.1481.

***6,7,8,9-Tetrahydro-1,6,6-trimethyl-2-(4-pyridinyl)-[3,2-c] furophenanthrene-10,11-dione (27c)***: deep red solid (8.7 mg from 0.05 mmol starting material, 47%); ^1^H NMR (400 MHz, CDCl_3_) δ_H_ = 8.75 (d, *J* = 4 Hz, 1H), 7.83 (d, *J* = 4 Hz, 1H), 7.73 (s, 1H), 7.67 (m, 2H), 7.55 (t, *J* = 8 Hz, 1H), 7.46 (dt, *J* = 8 Hz, 4 Hz, 2H), 3.21 (t, *J* = 8 Hz, 2H), 2.69 (s, 3H), 1.82 (m, 2H), 1.68 (m, 2H), 1.34 (s, 6H); ^13^C NMR (100 MHz, CDCl_3_) δ_C_ = 182.5 (C), 175.5 (C), 161.8 (C), 151.9 (C), 145.9 (CH), 145.2 (C), 133.7 (CH), 131.9 (CH), 128.5 (CH), 120.8 (CH), 119.8 (CH), 37.6 (CH_2_), 34.8 (C), 31.7 (CH_3_), 29.9 (CH_2_), 18.9 (CH_2_), 10.7 (CH_3_)；HR-EI-MS (*m/z*): calcd. for C_24_H_21_NO_3_ [M]^+^, 371.1521, found 371.1530.

***6,7,8,9-Tetrahydro-1,6,6-trimethyl-2-(3-thienyl)-[3,2-c] furophenanthrene-10,11-dione (27d)***: yellow solid (12.4 mg from 0.05 mmol starting material, 66%); ^1^H NMR (500 MHz, CDCl_3_) δ_H_ = 7.62 (dd, *J* = 20 Hz, 15 Hz, 2H), 7.57 (m, 1H), 7.46 (m, 2H), 3.18 (t, *J* = 5 Hz, 2H), 2.48 (s, 3H), 1.80 (m, 2H), 1.67 (m, 2H), 1.32 (s, 6H); ^13^C NMR (125 MHz, CDCl_3_) δ_C_ = 183.5 (C), 175.9 (C), 159.5 (C), 150.0 (C), 148.6 (C), 144.5 (C), 133.4 (CH), 130.8 (C), 127.3 (C), 126. 5(CH), 125.2 (CH), 121.6 (CH), 121.3 (C), 120.2 (CH), 115.4 (C), 37.8 (CH_2_), 34.6 (C), 31.8 (CH_3_), 29.8 (CH_2_), 19.1 (CH_2_), 9.7 (CH_3_); HR-EI-MS (*m/z*): calcd. for C_23_H_20_O_3_S [M]^+^, 376.1133, found 376.1121.

***6,7,8,9-Tetrahydro-1,6,6-trimethyl-2-(4-quinolinyl)-[3,2-c] furophenanthrene-10,11-dione (27e)***: red solid (20.4 mg from 0.05 mmol starting material, 97%); ^1^H NMR (400 MHz, CDCl_3_) δ_H_ = 9.34 (s, 1H), 8.65 (s, 1H), 8.10 (d, *J* = 8 Hz, 1H), 8.00 (d, *J* = 8 Hz, 1H), 7.79 (t, *J* = 8 Hz, 1H), 7.71 (t, *J* = 8Hz, 1H), 7.64 (dd, *J* = 16 Hz, 8 Hz, 2H), 3.23 (t, *J* = 4 Hz, 2H), 2.36 (s, 3H), 1.82 (m, 2H), 1.69 (m, 2H), 1.32 (s, 6H), 1.25 (s, 3H); ^13^C NMR (100 MHz, CDCl_3_) δ_C_ = 183.3 (C), 175.7 (C), 161.3 (C), 153.8 (CH), 150.5 (C), 148.7 (C), 144.7 (C), 144.3 (CH), 134.0 (C), 133.5 (CH), 131.3 (CH), 128.4 (C), 128.2 (CH), 127.7 (C), 127.1 (C), 126.6 (C), 124.5 (CH), 121.0 (C), 120.3 (CH), 119.9 (C), 37.7 (CH_2_), 34.6 (C), 31.8 (CH_3_), 29.9 (CH_2_), 29.6 (CH_2_), 19.0 (CH_2_), 9.9 (CH_3_); HR-EI-MS (*m/z*): calcd. for C_28_H_23_NO_3_ [M]^+^, 421.1678, found 421.1682.

**Procedure for the synthesis of 28a, 28b**

To a solution of tanshinone IIA (**1**, 29.4 mg, 0.1 mmol) in 1,4-dioxane (0.5 mL) were added aqueous HCHO solution (38%, 0.5 mL) and a drop of con. HCl successively. The resulting mixture was heated to reflux and was stirred at this temperature for 2 hours until starting material was fully converted by TLC. The mixture was cooled to the room temperature and was quenched with saturated aqueous NaHCO_3_ solution. The aqueous phase was extracted with EtOAc for three times. The combined organic layers were washed with water and saturated aqueous NaCl solution, dried over anhydrous Na_2_SO_4_ and concentrated in vacuum. The crude residue was subjected to flash column chromatography to give **28a** (8.0 mg, 23%) and **28b** (12.0 mg, 37%) as a red solid.

***6,7,8,9-Tetrahydro-2-[(hydroxymethoxy)-methyl]-1,6,6- trimethyl-[3,2-c]furophenanthrene-10,11-dione* *(28a)***: red solid; ^1^H NMR (400 MHz, CDCl_3_) δ_H_ = 7.56 (d, *J* = 8 Hz, 1H), 7.42 (d, *J* = 8 Hz, 1H), 4.62 (s, 2H), 3.89 (t, *J* = 4 Hz, 2H), 3.09 (dd, *J* = 8 Hz, 4 Hz, 2H), 2.95 (dd, *J* = 8 Hz, 4 Hz, 2H), 1.78 (m, 2H), 1.65 (m, 2H), 1.31 (s, 6H); ^13^C NMR (100 MHz, CDCl_3_) δ_C_ = 182.7 (C), 175.5 (C), 160.9 (C), 154.9 (C), 150.3 (C), 144.7 (C), 133.5 (CH), 126.9 (C), 126.1 (C), 120.2 (CH), 119.6 (C), 119.5 (C), 60.8 (CH_2_), 54.8 (CH_2_), 37.7 (CH_2_), 34.6 (C), 31.7 (CH_3_), 29.8 (CH_2_), 26.2 (CH_2_), 18.9 (CH_2_); ESI-MS(*m/z*): 377 [M+Na]**^+^**; HR-EI-MS (*m/z*): calcd. for C_21_H_22_O_5_ [M]^+^, 354.1467, found 354.1481.

***6,7,8,9-Tetrahydro-2-hydroxymethyl-1,6,6-trimethyl-[3,2-c] furophenanthrene-10,11-dione* *(28b)***: red solid;^1^H NMR (400 MHz, CDCl_3_) δ_H_ = 7.60 (d, *J* = 8 Hz, 1H), 7.52 (d, *J* = 8 Hz, 1H), 4.66 (s, 2H), 3.16 dd, *J* = 8 Hz, 4 Hz, 2H), 2.27 (s, 3H), 1.79 (m, 2H), 1.65 (m, 2H), 1.30 (s, 6H); ^13^C NMR (100 MHz, CDCl_3_) δ_C_ = 183.3 (C), 175.5 (C), 151.8 (C), 150.2 (C), 144.5 (C), 133.4 (CH), 127.0 (C), 126.4 (C), 120.3 (CH), 118.4 (C), 58.4 (CH_2_), 54.7 (CH_2_), 37.7 (CH_2_), 34.6 (C), 31.7 (CH_3_), 29.8 (CH_2_), 19.0 (CH_2_), 8.7 (CH_3_); HR-EI-MS (*m/z*): calcd. for C_20_H_20_O_4_ [M]^+^, 324.1362, found 324.1355.

**Typical procedure for the synthesis of 29a-29d (examplified by the synthesis of 33a)**

To a solution of (HCHO)n (30.0 mg, 1.0 mmol) in HOAc (0.5 mL) was added NHMe_2_-HCl (81.0 mg, 1.0 mmol) in one portion. The mixture was heated to reflux for 0.5 hours. Then tanshinone IIA (**1**, 29.4 mg, 0.1 mmol) was added. The resulting mixture was heated to reflux and was stirred at this temperature for another 2 hours until starting material was fully converted by TLC. The mixture was cooled to the room temperature and was quenched with saturated aqueous NaHCO_3_ solution. The aqueous phase was extracted with EtOAc (20 mL*3). The combined organic layers were washed with saturated aqueous NaCl solution, dried over anhydrous Na_2_SO_4_ and concentrated in vacuum. The crude residue was subjected to flash column chromatography to give **29a** (33.0 mg, 94%) as a red solid.

***6,7,8,9-Tetrahydro-2-[(dimethylamino)-methyl]-1,6,6- trimethyl-[3,2-c]furophenanthrene-10,11-dione* *(29a)***: red solid (33.0 mg from 0.1 mmol starting material, 94%); ^1^H NMR (400 MHz, CDCl_3_) δ_H_ = 7.61 (s, 2H), 3.51 (s, 2H), 3.18 (dd, *J* = 8 Hz, 4 Hz, 1H), 2.33 (s, 6H), 2.26 (s, 3H), 1.78-1.81 (m, 2H), 1.66-1.67 (m, 2H), 1.31 (s, 6H). ^13^C NMR (100 MHz, CDCl_3_) δ_C_ = 183.6 (C), 175.8 (C), 160.7 (C), 150.4 (C), 150.0 (C), 144.3 (C), 133.3 (CH), 127.2 (C), 126.4 (C), 120.5 (CH), 118.8 (C), 53.0 (CH_2_), 44.9 (CH_3_), 37.8 (CH_2_), 34.6 (C), 31.7 (CH_3_), 29.8 (CH_2_), 19.0 (CH_2_), 8.89 (CH_3_); HR-EI-MS (*m/z*): calcd. for C_22_H_25_NO_3_ [M]^+^, 351.1834, found 351.1835.

***6,7,8,9-Tetrahydro-2-(1-piperidinylmethyl)-1,6,6-trimethyl- [3,2-c]furophenanthrene-10,11-dione* *(29b)***: red solid (26.1 mg from 0.1 mmol starting material, 67%); ^1^H NMR (400 MHz, CDCl_3_) δ_H_ = 7.98 (s, 1H), 7.58 (s, 1H), 3.55 (s, 2H), 3.46 (dd, *J* = 8 Hz, 4 Hz, 2H), 3.30 (dd, *J* = 8 Hz, 4 Hz, 2H), 3.14 (t, *J* = 8 Hz, 2H), 2.46 (s, 3H), 2.22 (s, 2H), 1.76 (d, *J* = 8 Hz, 2H), 1.51-1.61 (m, 12H), 1.40 (s, 2H), 1.28 (s, 6H); ^13^C NMR (101 MHz, CDCl_3_) δ_C_ = 183.6 (C), 175.7 (C), 160.7 (C), 149.9 (C), 144.3 (C), 133.3 (CH), 127.3 (C), 126.3 (C), 120.5 (CH), 119.0 (C), 53.9 (CH_2_), 52.6 (CH_2_), 46.7 (CH_2_), 40.5 (CH_2_), 37.7 (CH_2_), 34.5 (C), 31.7 (CH_3_), 29.8 (CH_2_), 26.5 (CH_2_), 25.6 (CH_2_), 25.0 (CH_2_), 24.6 (CH_2_), 23.8 (CH_2_), 19.0 (CH_2_), 9.0 (CH_3_); HR-EI-MS (*m/z*): calcd. for C_25_H_29_NO_3_ [M]^+^, 391.2147, found 391.2140.

***6,7,8,9-Tetrahydro-2-(4-morpholinylmethyl)-1,6,6-trimethyl- [3,2-c]furophenanthrene-10,11-dione (29c)****:* red solid (28.3 mg from 0.1 mmol starting material, 72%); ^1^H NMR (400 MHz, CDCl_3_) δ_H_ = 7.60 (d, *J* = 8 Hz, 1H), 7.56 (d, *J* = 8 Hz, 1H), 3.71-3.72 (m, 4H), 3.56 (s, 2H), 3.14 (dd, *J* = 8 Hz, 4 Hz, 2H), 2.52 (s, 4H), 2.23 (s, 3H), 1.75-1.78 (m, 2H), 1.61-1.64 (m, 2H), 1.28 (s, 6H); ^13^C NMR (100 MHz, CDCl_3_) δ_C_ = 183.4 (C), 175.6 (C), 160.7 (C), 150.0 (C), 149.3 (C), 144.3 (C), 133.3 (CH), 127.1 (C), 126.3 (C), 120.4 (CH), 120.2 (C), 119.3 (C), 66.6 (CH_2_), 53.1 (CH_2_), 52.4 (CH_2_), 37.7 (CH_2_), 34.5 (C), 31.7 (CH_3_), 29.8 (CH_2_), 19.0 (CH_2_), 8.9 (CH_3_); HR-EI-MS (*m/z*): calcd. for C_24_H_27_NO_4_ [M]^+^, 393.1940, found 393.1935.

***6,7,8,9-Tetrahydro-2-[(4-methyl-1-piperidinyl) methyl]-1,6,6-trimethyl-[3,2-c]furophenanthrene-10,11-dione* *(29d)***: red solid (31.6 mg from 0.1 mmol starting material, 78%); ^1^H NMR (400 MHz, CDCl_3_) δ_H_ = 7.59 (d, *J* = 8 Hz, 1H), 7.55 (d, *J* = 8 Hz, 1H), 3.57 (s, 2H), 3.13 (dd, *J* = 8 Hz, 4 Hz, 2H), 2.68 (s, 8H), 2.42 (s, 3H), 2.21 (s, 3H), 1.76 (d, *J* = 4 Hz, 2H), 1.63 (d, *J* = 4 Hz, 2H), 1.27 (s, 6H); ^13^C NMR (100 MHz, CDCl_3_) δ_C_ = 183.9 (C), 176.0 (C), 161.1 (C), 150.5 (C), 149.9 (C), 144.8 (C), 133.7 (CH), 127.6 (C), 126.8 (C), 120.8 (CH), 120.6 (C), 119.7 (C), 54.7 (CH_2_), 52.2 (CH_2_), 51.9 (CH_2_), 45.4 (CH_3_), 38.2 (CH_2_), 35.0 (C), 32.2 (CH_3_), 30.2 (CH_2_), 29.6 (CH_2_), 19.4 (CH_2_), 9.3 (CH_3_); HR-EI-MS (*m/z*): calcd. for C_25_H_30_N_2_O_3_ [M]^+^, 406.2256, found 406.2257.

**Typical procedure for the synthesis of 30 and 34 (examplified by the synthesis of 30)**

To a solution of tanshinone IIA (**1**, 147.0 mg, 0.5 mmol) in AcOH (1.0 mL) was added SeO_2_ (55.0 mg, 0.5 mmol) in one portion. The mixture was heated to 130 ^o^C and was stirred at this temperature for 15 min until no starting material was detected according to TLC. The mixture was cooled to the room temperature and was quenched with saturated aqueous NaHCO_3_ solution. The aqueous phase was extracted with EtOAc (25 mL*3). The combined organic layers were washed with water and saturated aqueous NaCl solution, dried over anhydrous Na_2_SO_4_ and concentrated in vacuum. The crude residue was subjected to flash column chromatography to give **30** (127.1 mg, 82%) as a red solid.

***6,7,8,9-Tetrahydro-1-hydroxymethyl-6,6-dimethyl-[3,2-c] furophenanthrene-10,11-dione* *(30)***: The spectral data are identical to those of previous report. red solid; ^1^H NMR (400 MHz, CDCl_3_) δ_H_ = 7.66 (d, *J* = 8 Hz, 1H), 7.58 (d, *J* = 8 Hz, 1H), 7.39 (s, 1H), 4.66 (s, 2H), 3.18 (t, *J* = 4 Hz, 2H) , 1.79 (m, 2H), 1.65 (m, 2H), 1.60 (m, 2H), 1.31 (s, 6H); ^13^C NMR (100 MHz, CDCl_3_) δ_C_ = 151.1 (C), 145.1 (C), 140.4 (CH), 133.7 (CH), 125.7 (C), 120.6 (CH), 55.6 (CH_2_), 37.6 (CH_2_), 34.7 (C), 31.7 (CH_3_), 29.9 (CH_2_), 29.9 (CH_2_), 19.0 (CH_2_); HR-EI-MS (*m/z*): calcd. for C_19_H_18_O_4_ [M]^+^, 310.1205, found 310.1202.

***1-hydroxymethyl-6,6-dimethyl-[3,2-c]furophenanthrene- 7,10,11(6H)-trione* *(34)*:** (10.3 mg from 0.1 mmol compound **13**, 31% yield); ^1^H NMR (400 MHz, CDCl_3_) δ_H_ = 8.99 (d, *J* = 10.5 Hz, 1H), 7.83 (d, *J* = 8.2 Hz, 1H), 7.75 (d, *J* = 8.2 Hz, 1H), 7.47 (s, 1H), 6.44 (d, *J* = 10.5 Hz, 1H), 4.71 (d, *J* = 6.6 Hz, 2H), 3.38 (t, *J* = 7.0 Hz, 1H), 1.56 (s, 6H) ; ^13^C NMR (100 MHz, CDCl_3_) δ_C_ = 201.66, 183.30, 175.46, 161.89, 151.56, 141.38, 138.85, 132.75, 132.49, 128.96, 128.00, 126.12, 125.12, 123.85, 120.05, 55.17, 48.04, 27.54 ; HR-ESI-MS (*m/z*): calcd. for C_19_H_14_O_5_Na [M+Na]^+^, 345.0733, found 345.0744.

**Procedure for the synthesis of 31a-31b.**

**Step 1:** To a solution of **30** (15.5 mg, 0.05 mmol) and CBr_4_ (19.8 mg, 0.6 mmol) in DCM (0.7 mL) at 0^o^C under argon was added a solution of PPh_3_ (26.2 mg, 0.1 mmol) in DCM (0.3 mL) dropwise. The mixture was warmed to the room temperature and stirred for 0.5 hour until no starting material was detected according to TLC. The solvent was removed in vacuum. The crude residue was directly subjected to flash column chromatography to give the bromide (17.0 mg, 91%) as a red solid.

**Step 2:** To a solution of the bromide (7.5 mg, 0.02 mmol) in THF (0.8 mL) at 0 ^o^C under argon were added Et_3_N (12.0 mg, 0.12 mmol) and aniline (9.5 mg, 0.11 mmol) successively. The mixture was stirred at room temperature for 1 hour until no starting material was detected according to TLC. The solvent was removed in vacuum. The crude residue was directly subjected to flash column chromatography to give **31a** (6.5 mg, 87%) as a red solid.

***6,7,8,9-Tetrahydro-1-[(phenylamino)methyl]-6,6-dimethyl- [3,2-c]furophenanthrene-10,11-dione* *(31a)***: red solid (9.3 mg from 0.03 mmol starting material, 81%); ^1^H NMR (400 MHz, CDCl_3_) δ_H_ = 7.63 (d, *J* = 8 Hz, 1H), 7.53 (d, *J* = 8 Hz, 1H), 7.40 (s, 1H), 7.16 (m, 2H), 6.70 (t, *J* = 8 Hz, 1H), 6.66 (d, *J* = 8 Hz, 2H), 4.39 (s, 2H), 3.18 (t, *J* = 4 Hz, 2H), 1.79 (m, 2H), 1.65 (m, 2H), 1.30 (s, 6H), 1.25 (s, 2H); ^13^C NMR (100 MHz, CDCl_3_) δ_C_ = 182.9 (C), 175.4 (C), 150.6 (C), 147.2 (C), 144.8 (C), 141.8 (CH), 133.5 (CH), 129.1 (CH), 127.0 (C), 123.8 (C), 120.4 (CH), 117.9 (CH), 115.0 (C), 113.6 (CH), 37.7 (CH_2_), 37.7 (CH_2_), 34.6 (C), 31.7 (CH_3_), 29.9 (CH_2_), 29.6 (CH_2_), 19.0 (CH_2_); HR-EI-MS (*m/z*): calcd. for C_25_H_23_NO_3_ [M]^+^, 385.1678, found 385.1678.

**31b**: 92% yield, red solid (6.5 mg from 0.02 mmol starting material, 92%); ^1^H NMR (400 MHz, MeOD) δ_H_ = 7.66 (d, *J* = 8 Hz, 1H), 7.59 (d, *J* = 8 Hz, 1H), 3.83 (d, *J* = 20 Hz, 4H), 3.78 (s, 2H), 3.18 (dd, *J* = 8 Hz, 4 Hz, 2H), 2.69 (d, *J* = 12 Hz, 4H), 1.78-1.81 (m, 2H), 1.67 (d, *J* = 4 Hz, 4H), 1.31 (s, 6H); ^13^C NMR (100 MHz, MeOD) δ_C_ = 183.0 (C), 175.4 (C), 162.1 (C), 153.1 (C), 150.6 (C), 144.7 (C), 133.5 (CH), 127.0 (C), 126.3 (C), 120.4 (CH), 66.2 (CH_2_), 52.8 (CH_2_), 37.7 (CH_2_), 34.6 (C), 31.7 (CH_3_), 29.8 (CH_2_), 29.2 (CH_2_), 19.0 (CH_2_); HR-EI-MS (*m/z*): calcd. for C_23_H_25_NO_4_ [M]^+^, 379.1784, found 379.1781.

**Typical procedure for the synthesis of 32 and 33 (examplified by the synthesis of 32):**

To a solution of **13** (14.6 mg, 0.05 mmol) in DCM (1.0 mL) at -40 ^o^C were added NBS (13.4 mg, 0.15 mmol) and TFA (5.7 mg, 0.1 mmol). The mixture was stirred at room temperature for 3 hours until starting material was fully converted by TLC. The mixture was quenched with saturated aqueous Na_2_SO_3_ solution and was extracted with EtOAc for three times. The combined organic layers were washed with water and saturated aqueous NaCl solution, dried over anhydrous Na_2_SO_4_ and concentrated in vacuum. The crude residue was subjected to flash column chromatography to give **33** (14.7 mg, 74%) as a deep red solid.

***6,7,8,9-Tetrahydro-6,6-trimethyl-1-hydroxymethyl-2-chloro- [3,2-c]furophenanthrene-10,11-dione* *(32)***: red solid (16.7 mg from 0.05 mmol starting material, 97%); ^1^H NMR (400 MHz, CDCl_3_) δ_H_ = 7.65 (d, *J* = 8 Hz, 1H), 7.54 (d, *J* = 8 Hz, 1H), 4.60 (s, 2H), 3.16 (t, *J* = 8 Hz, 2H), 1.76-1.80 (m, 2H), 1.65 (dd, *J* = 8 Hz, 4 Hz, 2H), 1.30 (s, 6H); ^13^C NMR (100 MHz, CDCl_3_) δ_C_ = 181.7 (C), 175.1 (C), 161.2 (C), 151.6 (C), 145.4 (C), 136.6 (C), 133.7 (CH), 125.9 (C), 120.5 (CH), 119.8 (C), 54.7 (CH_2_), 37.6 (CH_2_), 34.7 (C), 31.7 (CH_3_), 30.0 (CH_2_), 18.9 (CH_2_); HR-EI-MS (*m/z*): calcd. for C_19_H_17_ClO_4_ [M]^+^, 344.0815, found 344.0818.

***6,7-Dihydro-1,6,6-trimethyl-2-bromo-[3,2-c]furophenanthrene- 10,11-dione (33)***: red solid (13.7. mg from 0.05 mmol starting material, 74%); ^1^H NMR (400 MHz, CDCl_3_) δ_H_ = 7.81 (d, *J* = 10.2 Hz, 1H), 7.51 (d, *J* = 8.0 Hz, 1H), 7.46 (d, *J* = 7.9 Hz, 1H), 6.39-6.21 (m, 1H), 2.27 (dd, *J* = 4.4, 1.4 Hz, 2H), 2.19 (s, 3H), 1.28 (s, 6H); ^13^C NMR (100 MHz, CDCl_3_) δ_C_ = 183.1, 174.5, 161.5, 148.9, 137.6, 134.5, 130.4, 126.3, 124.8, 124.3, 122.6, 121.5, 120.7, 120.4, 37.7, 34.2, 28.3, 9.4; HR-ESI-MS (*m/z*): calcd. for C_19_H_15_BrO_3_Na [M+Na]^+^, 393.0099, found 393.0099.

**Procedure for the synthesis of 35**

To a solution of **16** (166.0 mg, 0.47 mmol) in 1,4-dioxane (4.0 mL) was added SeO_2_ (130.0 mg, 1.2 mmol). The mixture was heated to 100 ^o^C and was stirred at this temperature overnight before another portion of SeO_2_ (130.0 mg, 1.2 mmol) was added to the mixture. The mixture was cooled to the room temperature and the solvent was removed in vacuum. Water was added to dilute the mixture. The aqueous phase was extracted with EtOAc (25 mL*3). The combined organic layers were washed with water and saturated aqueous NaCl solution, dried over anhydrous Na_2_SO_4_ and concentrated in vacuum. The crude residue was subjected to flash column chromatography to give **35** (88.0 mg, 51%) as a red solid.

***6,7,8,9-Tetrahydro-1-hydroxymethyl-7-acetoxy-6,6-dimethyl- [3,2-c]furophenanthrene-10,11-dione* *(35)***: red solid; ^1^H NMR (400 MHz, CDCl_3_) δ_H_ = 7.66 (d, *J* = 8.2 Hz, 1H), 7.63 (d, *J* = 8.2 Hz, 1H), 7.41 (s, 1H), 5.04-4.95 (m, 1H), 4.67 (s, 2H), 3.31 (t, *J* = 6.6 Hz, 2H), 2.08-2.01 (m, 5H, overlap), 1.34 (s, 3H), 1.33 (s, 3H); ^13^C NMR (100 MHz, CDCl_3_) δ_H_ = 182.6, 175.6, 170.7, 162.8, 148.8, 143.2, 140.8, 133.6, 127.3, 125.9, 125.9, 121.1, 119.6, 75.6, 55.2, 38.5, 30.1, 25.9, 25.5, 22.9, 21.1.


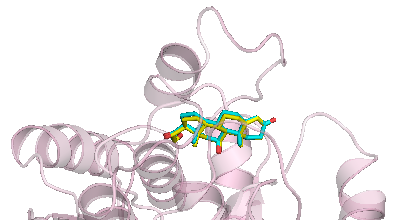

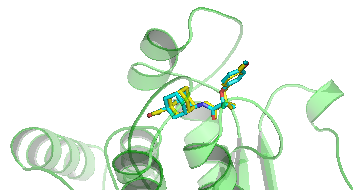


**1Y5R 2IRW**

RMSD=0.33 RMSD=0.65

**Figure S1.** Validation of the adopted docking procedure was conducted via re-docking of the co-crystallized ligands (corticosterone for 1Y5R and adamantane ether for 2IRW) and calculating RMSD between docked and re-docked ligands. The RMSD values between the top-scoring ligand orientation and the crystal ligand orientation 1Y5R and 2IRW were 0.33 and 0.65 Å, respectively, which were less than 2.0 Å.


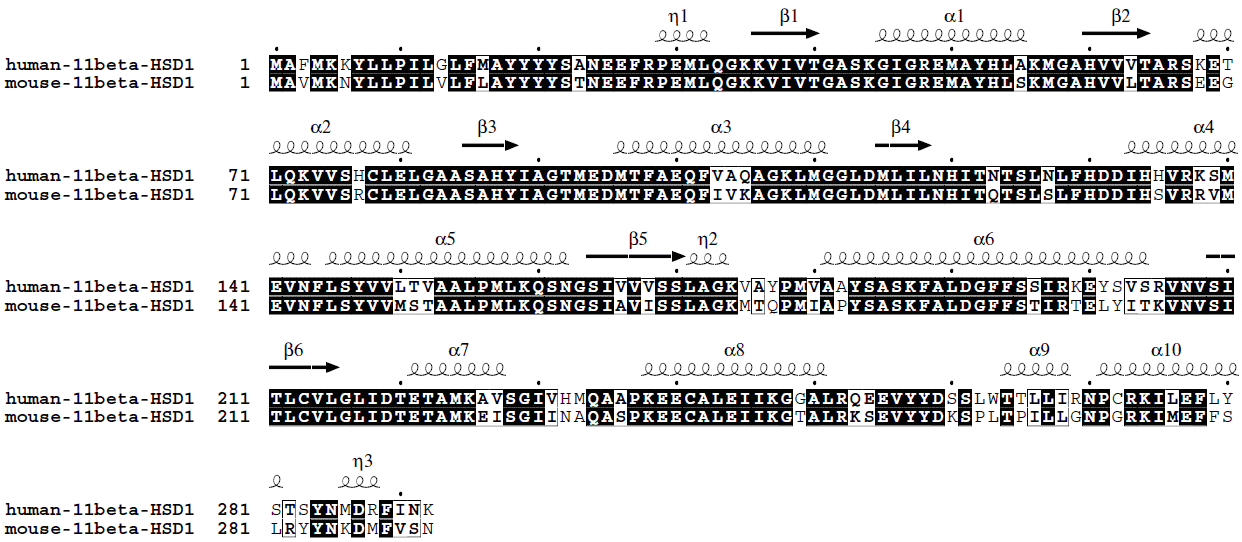


**Figure S2.** Sequence alignment of human 11*β*-HSD1 with mouse 11*β*-HSD1 obtained with the ClustalW program. The figure was prepared using the ESPript program (http://espript.ibcp.fr/ESPript/ESPript/). Secondary structures of 11*β*-HSD1: helices (shown as springs) and *β*-sheets (presented as arrows) are plotted on the top of the alignment. Amino acids conserved in both enzymes are colored white and shown in black boxes, while similar amino acid (similarity defined by default parameters in ESPript) are colored black and shown in white boxes.


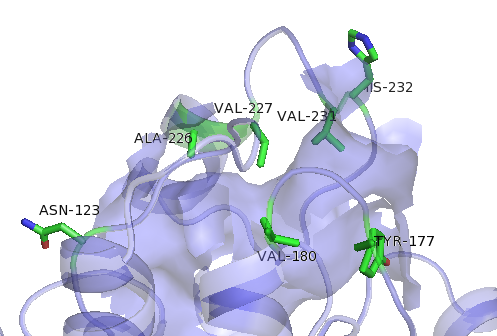


**Human 11*β*-HSD1 (PDB code: 2IRW)**


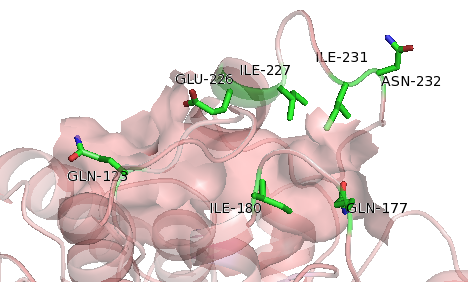


**Mouse 11*β*-HSD1. (PDB code: 1Y5R)**

**Figure S3.** Critical different residues of the binding pocket are labeled in the 3D interaction views for human and mouse 11*β*-HSD1. The residues are shown as sticks (receptor carbon in green).

**Figure S4. The ^1^H NMR spectra copy of compound 9 in CDCl_3_ (400 MHz)**


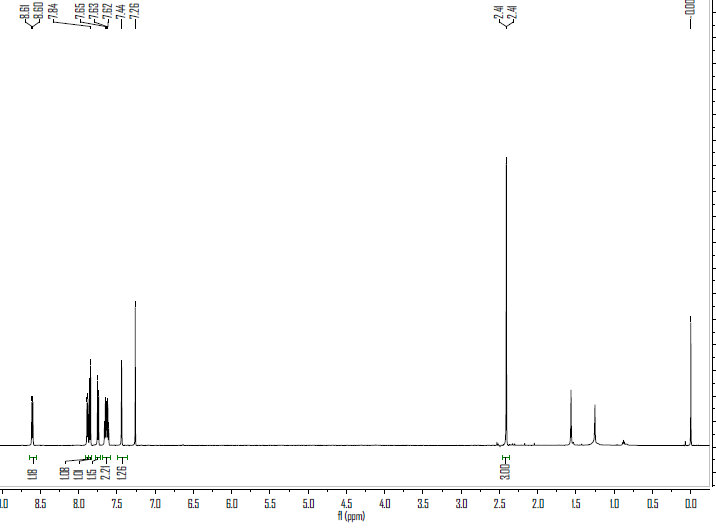


**Figure S5. The ^13^C NMR spectra copy of compound 9 in CDCl_3_ (101 MHz)**


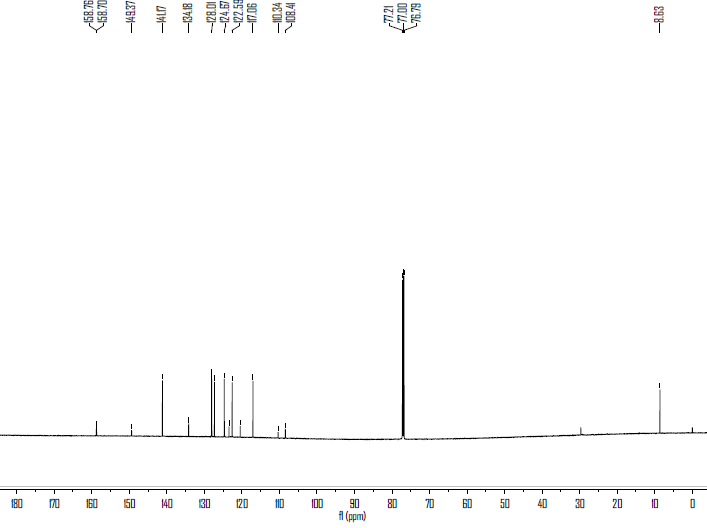


**Figure S6. The ^1^H NMR spectra copy of compound 10 in CDCl_3_ (400 MHz)**


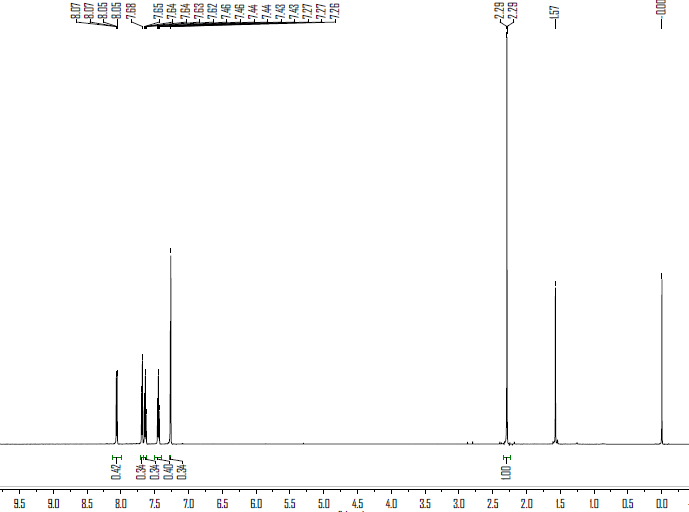


**Figure S7. The ^13^C NMR spectra copy of compound 10 in CDCl_3_ (101 MHz)**


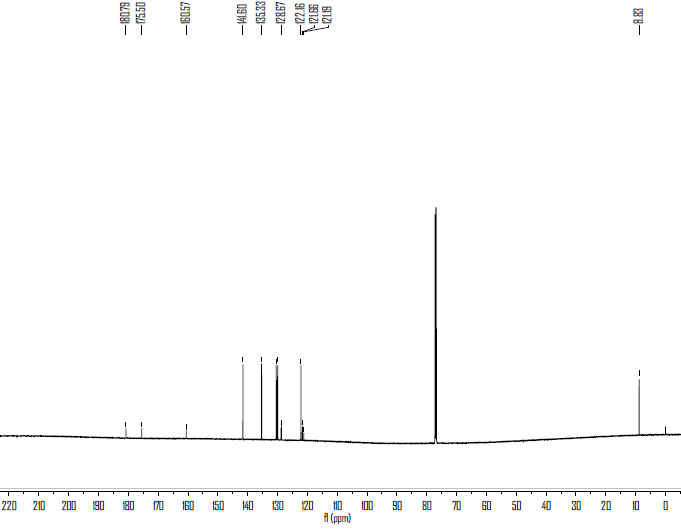


**Figure S8. The ^1^H NMR spectra copy of compound 11 in *d_6_*-DMSO (400 MHz)**


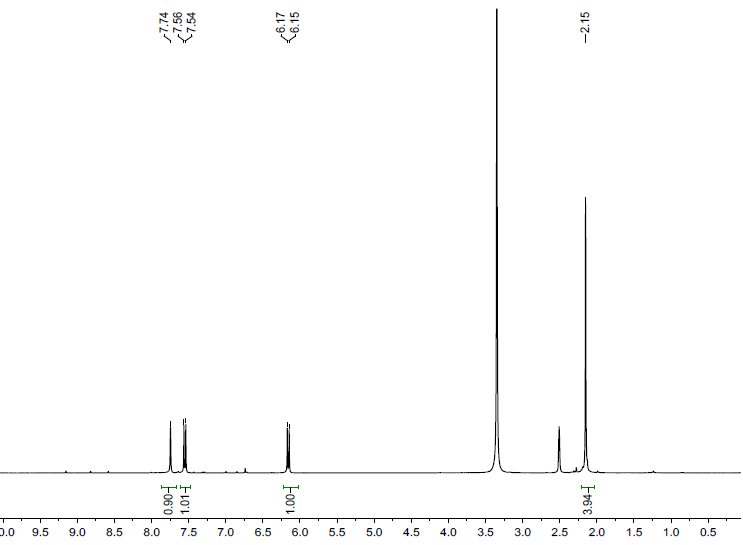


**Figure S9. The ^13^C NMR spectra copy of compound 11 in *d_6_*-DMSO (101 MHz)**


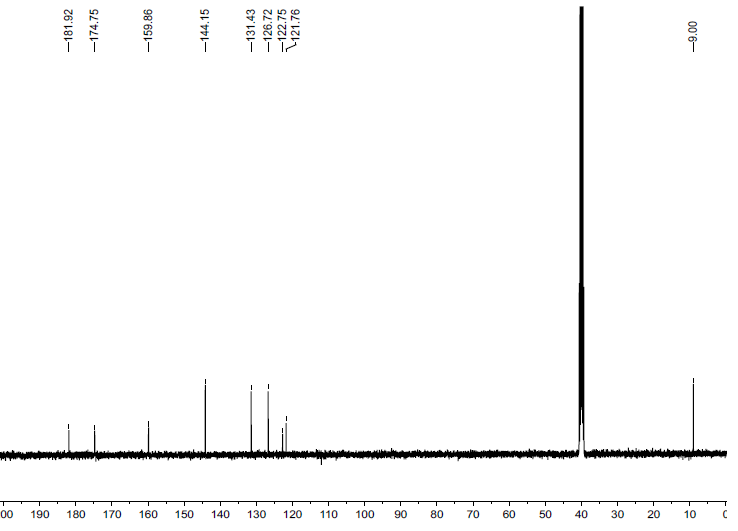


**Figure S10. The ^1^H NMR spectra copy of compound 12 in CDCl_3_ (400 MHz)**


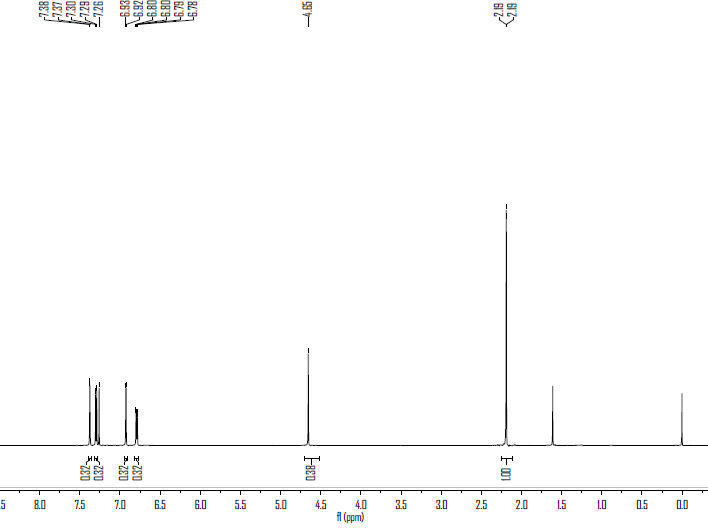


**Figure S11. The ^13^C NMR spectra copy of compound 12 in CDCl_3_ (101 MHz)**


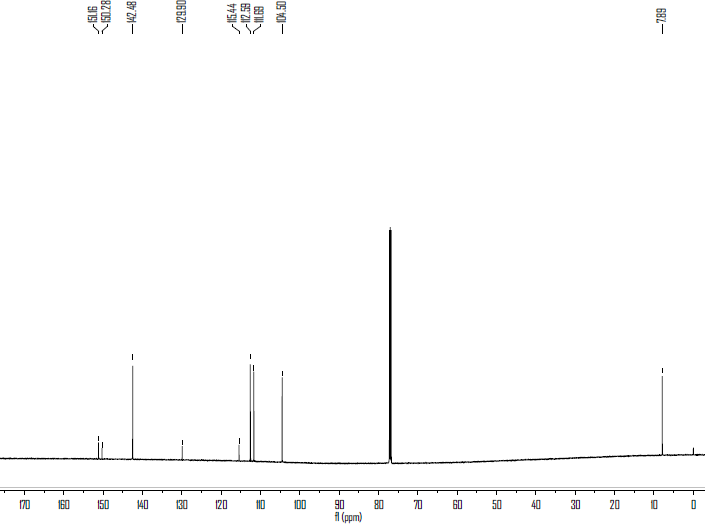


**Figure S12. The ^1^H NMR spectra copy of compound 13 in CDCl_3_ (400 MHz)**


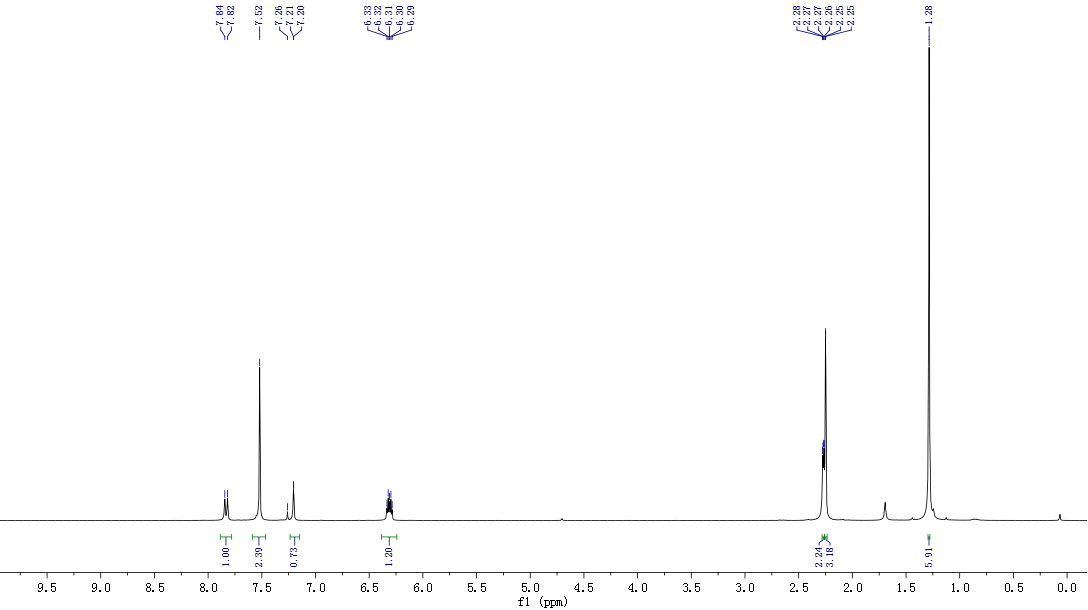


**Figure S13. The ^13^C NMR spectra copy of compound 13 in CDCl_3_ (101 MHz)**

**Figure S14. The ^1^H NMR spectra copy of compound 14 in CDCl_3_ (400 MHz)**


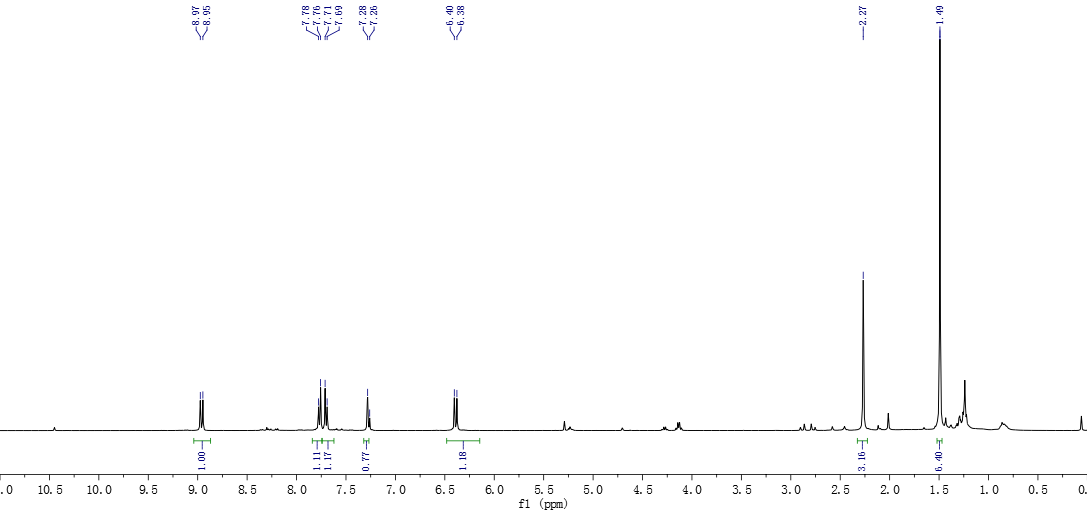


**Figure S15. The ^13^C NMR spectra copy of compound 14 in CDCl_3_ (101 MHz)**


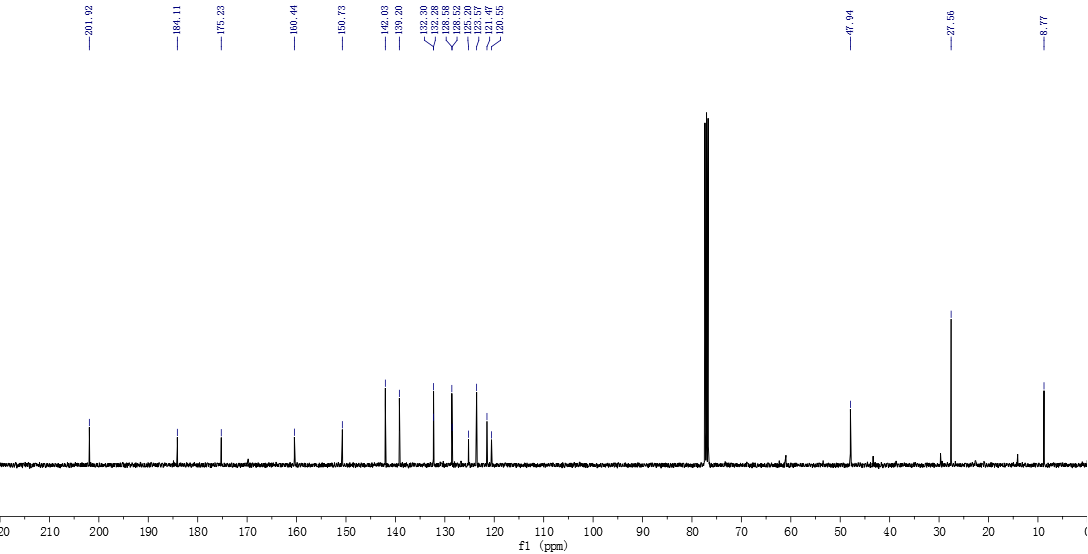


**Figure S16. The ^13^C NMR spectra copy of compound 15 in CDCl_3_ (101 MHz)**


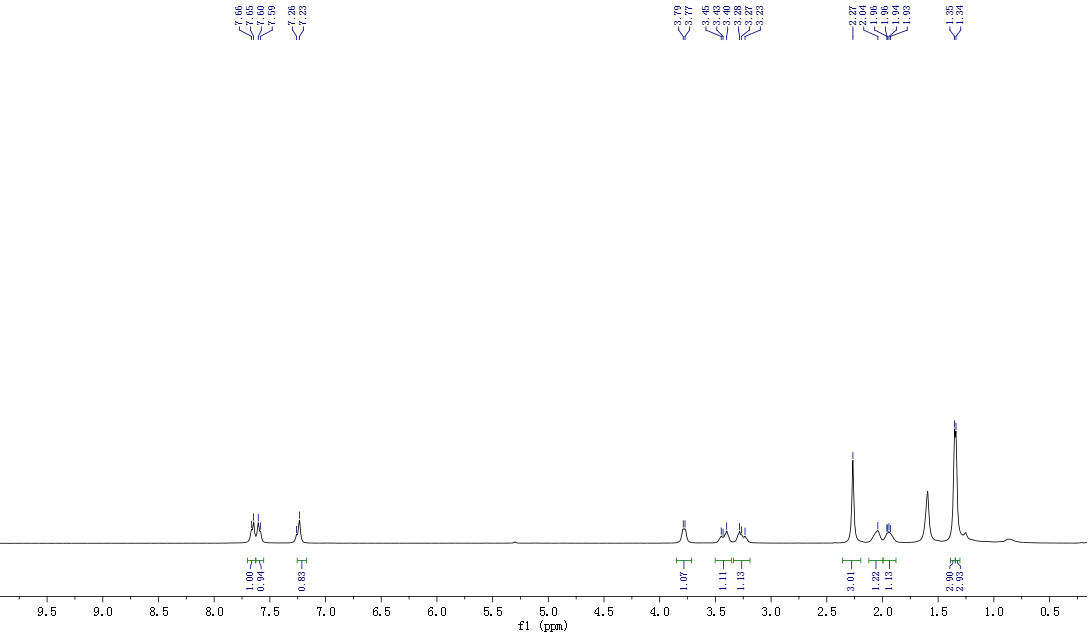


**Figure S17. The ^13^C NMR spectra copy of compound 15 in CDCl_3_ (101 MHz)**


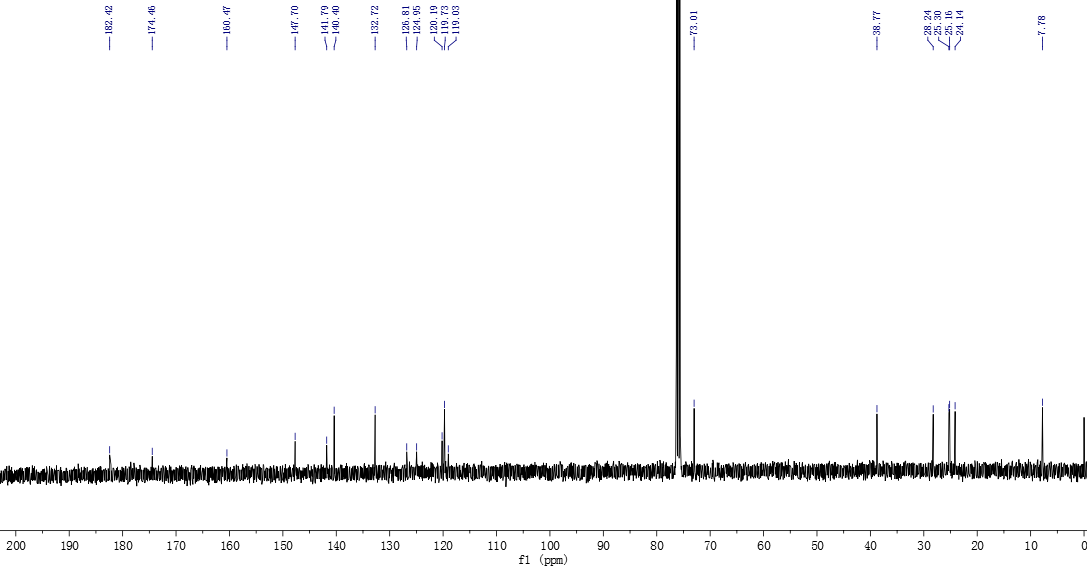


**Figure S18. The ^1^H NMR spectra copy of compound 16 in CDCl_3_ (400 MHz)**


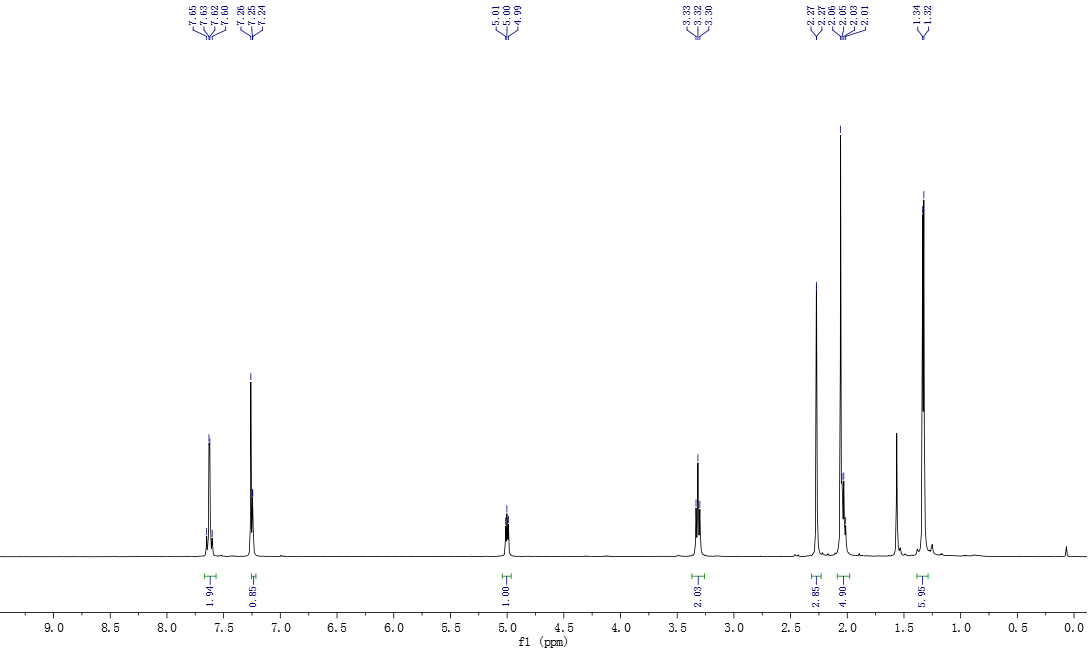


**Figure S19. The ^13^C NMR spectra copy of compound 16 in CDCl_3_ (101 MHz)**


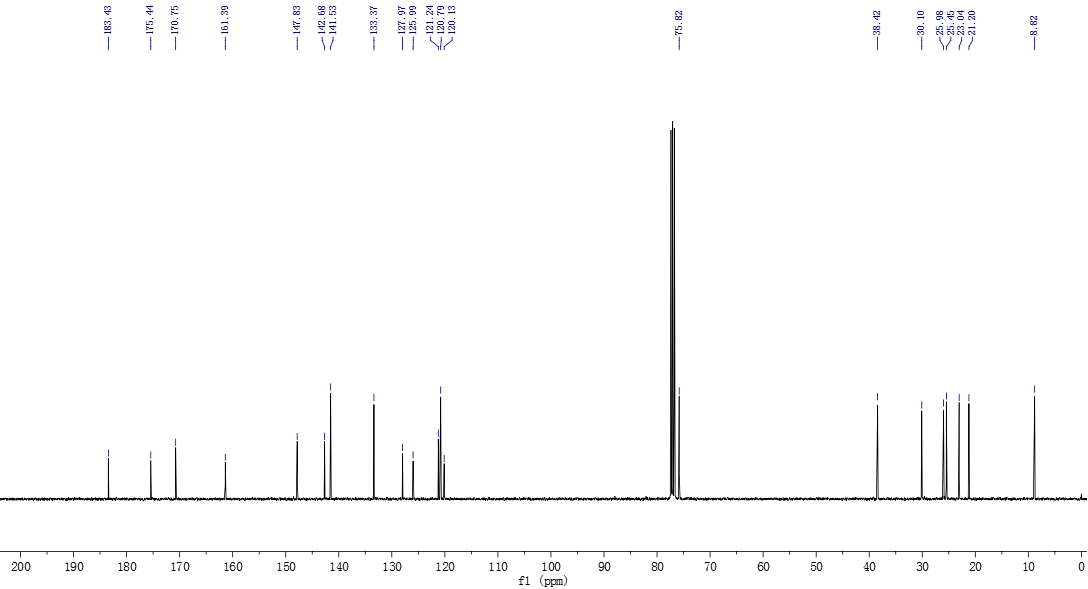


**Figure S20. The ^1^H NMR spectra copy of compound 17 in CDCl_3_ (400 MHz)**


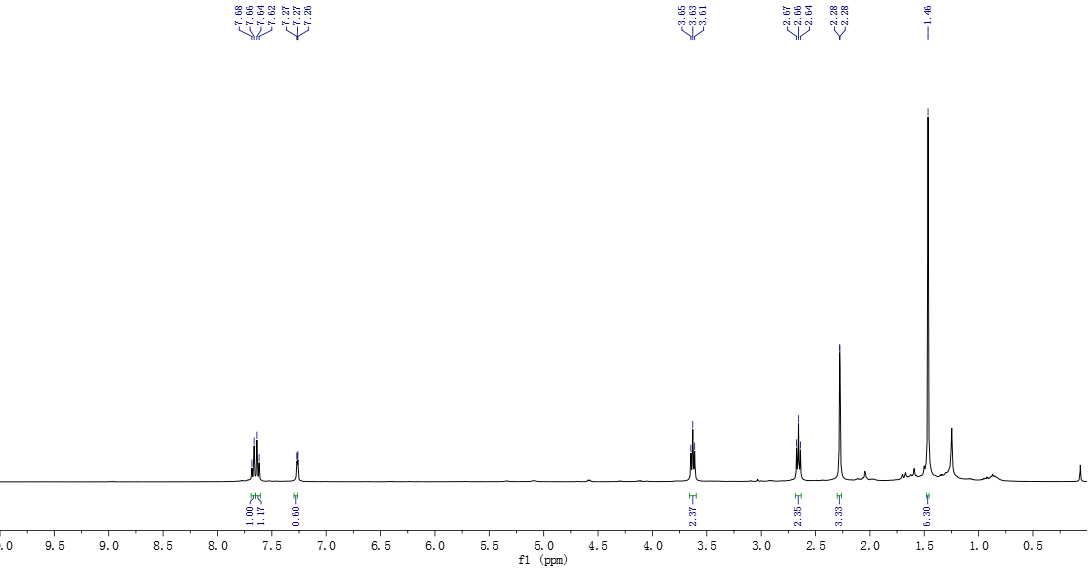


**Figure S21. The ^13^C NMR spectra copy of compound 17 in CDCl_3_ (101 MHz)**


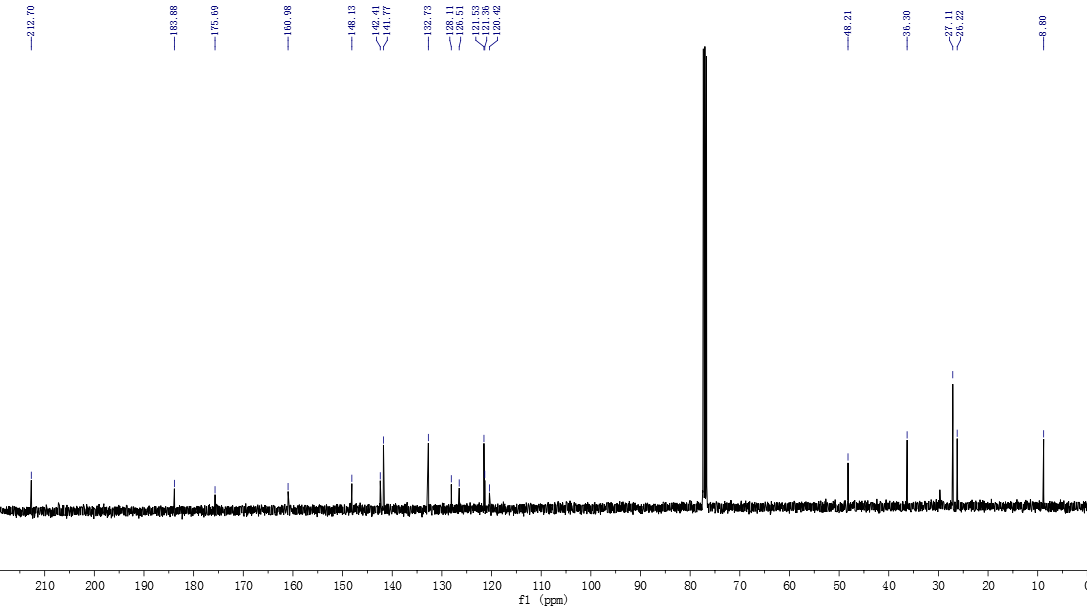


**Figure S22. The ^1^H NMR spectra copy of compound 18a in CDCl_3_ (400 MHz)**


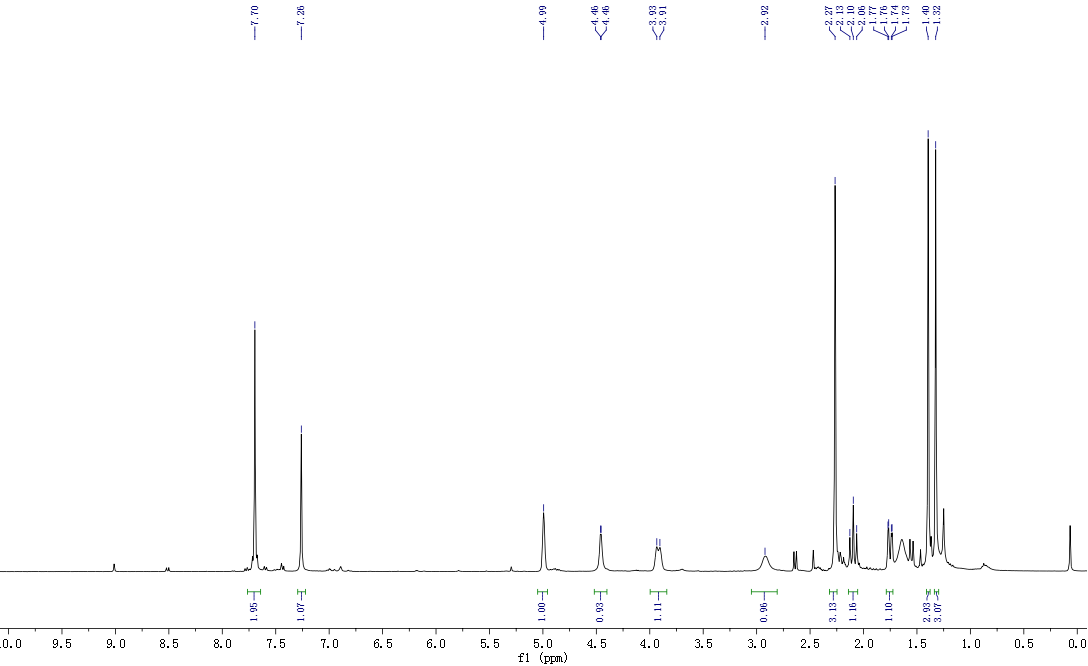


**Figure S23. The ^13^C NMR spectra copy of compound 18a in CDCl_3_ (101 MHz)**


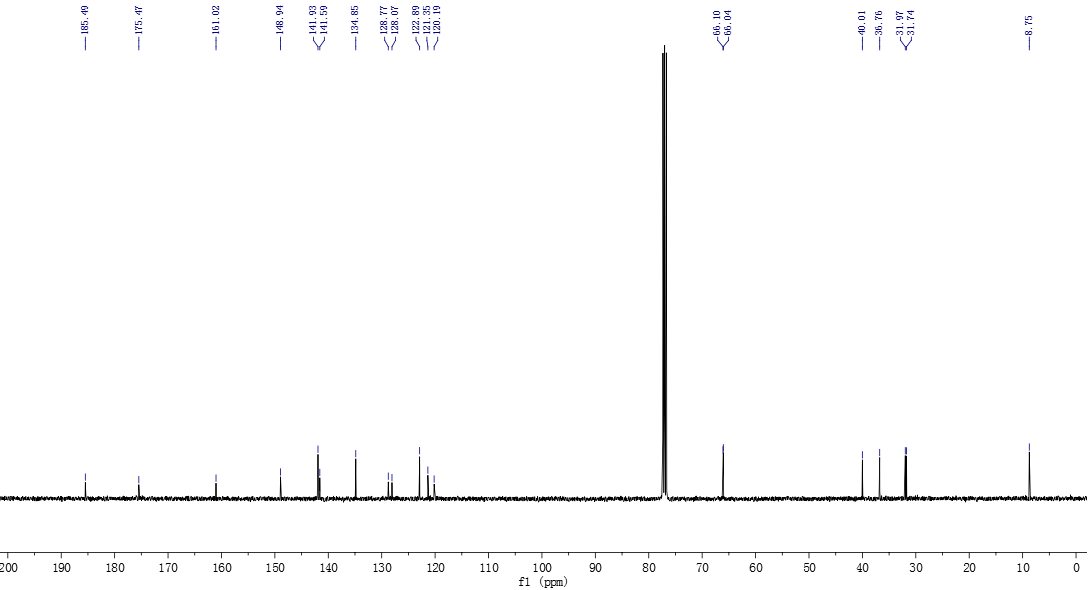


**Figure S24. The ^1^H NMR spectra copy of compound 18b in CDCl_3_ (400 MHz)**


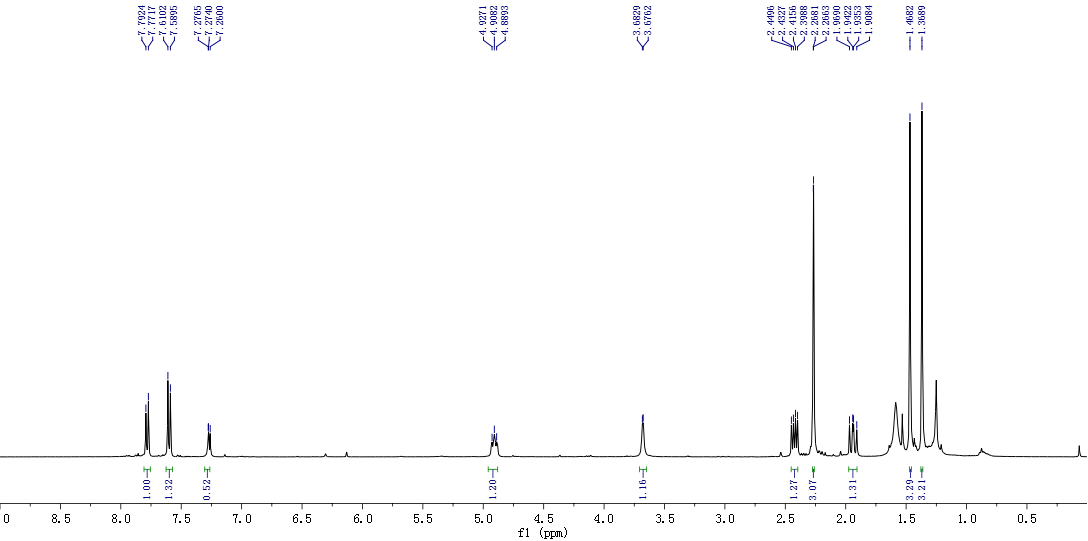


**Figure S25. The ^13^C NMR spectra copy of compound 18b in CDCl_3_ (101 MHz)**


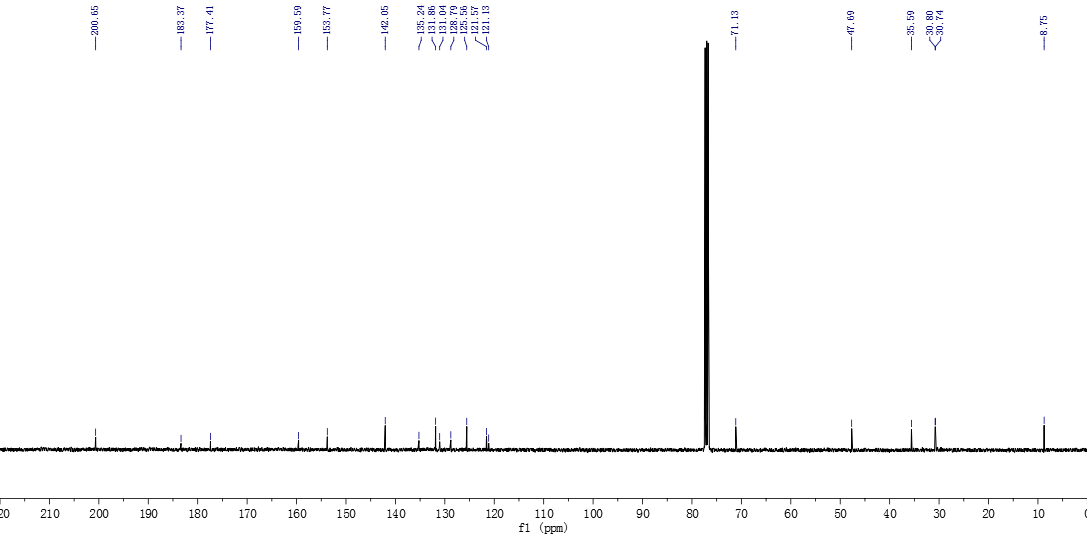


**Figure S26. The ^1^H NMR spectra copy of compound 19 in CDCl_3_ (400 MHz)**


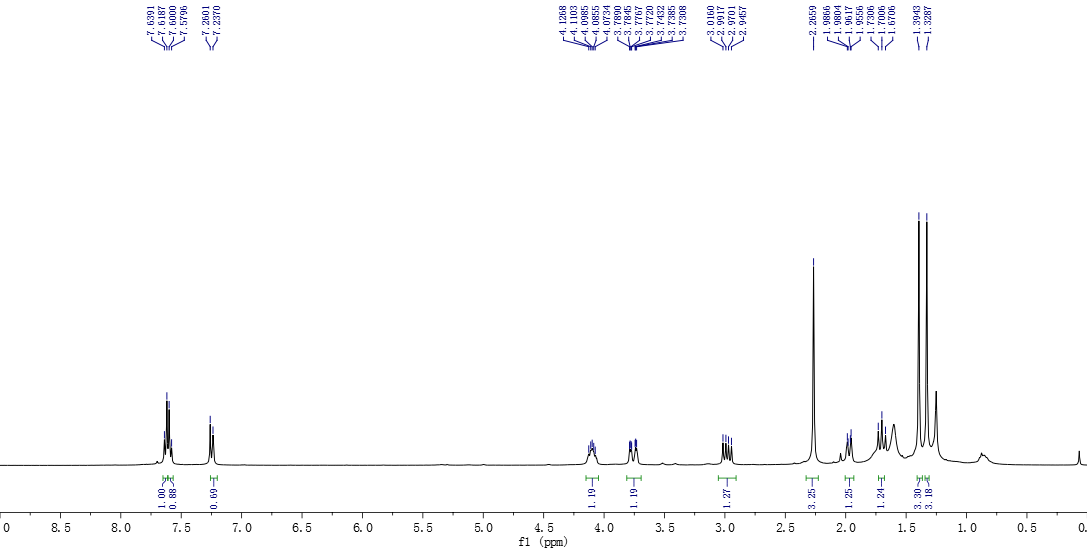


**Figure S27. The ^13^C NMR spectra copy of compound 19 in CDCl_3_ (101 MHz)**


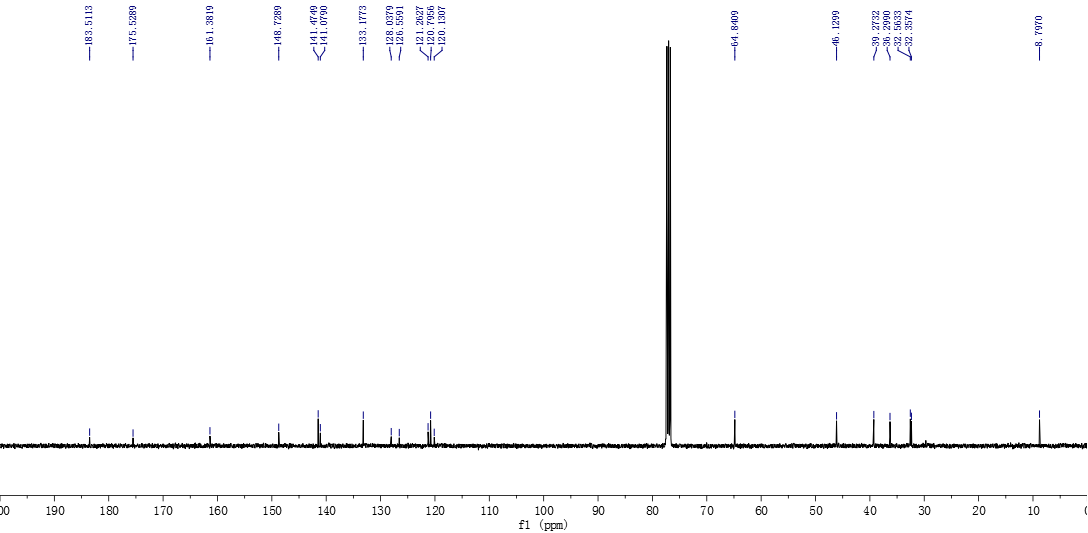


**Figure S28. The ^1^H NMR spectra copy of compound 20 in CDCl_3_ (400 MHz)**


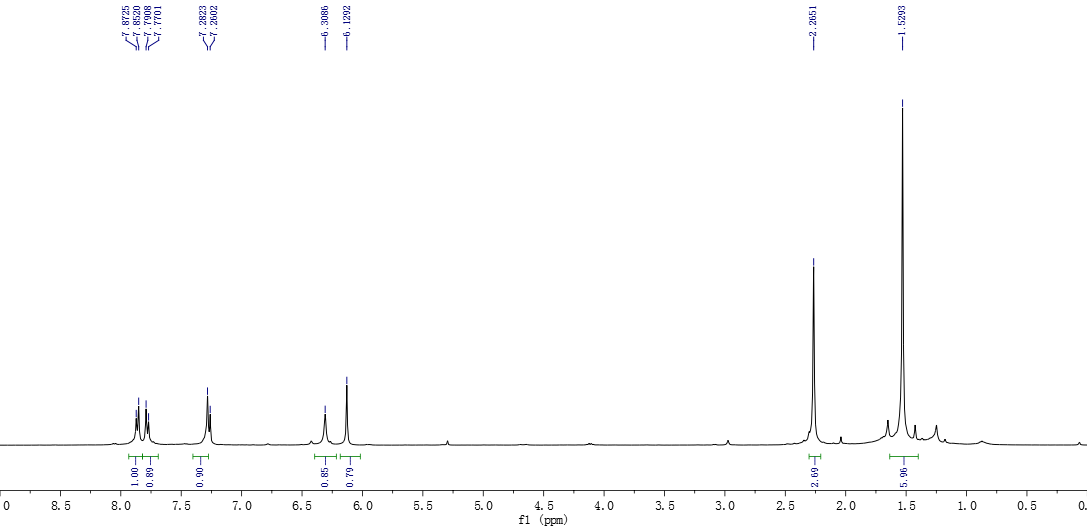


**Figure S29. The ^13^C NMR spectra copy of compound 20 in CDCl_3_ (101 MHz)**


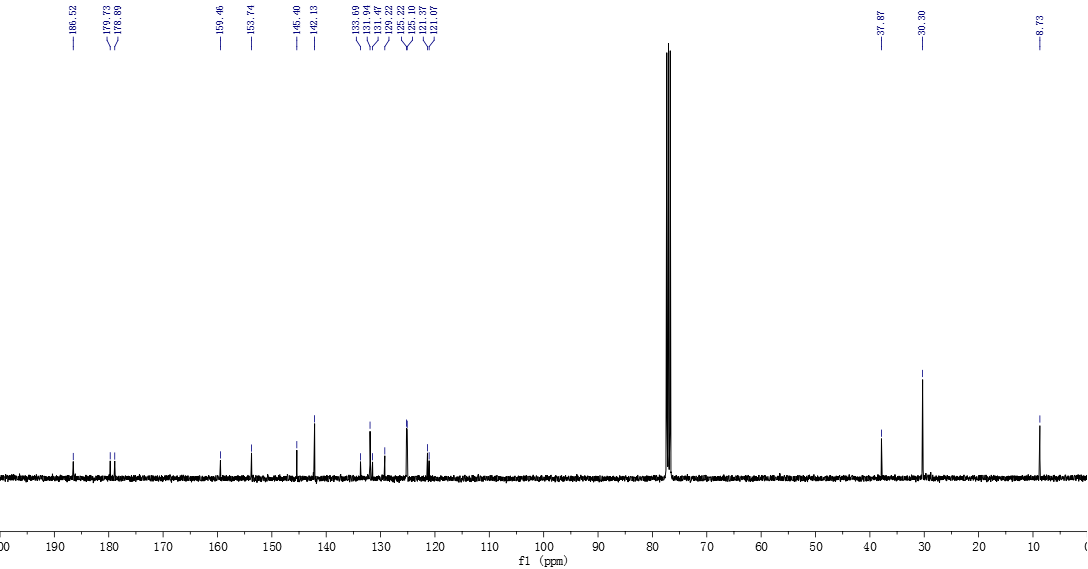


**Figure S30 The ^1^H NMR spectra copy of compound 21 in CDCl_3_ (400 MHz)**


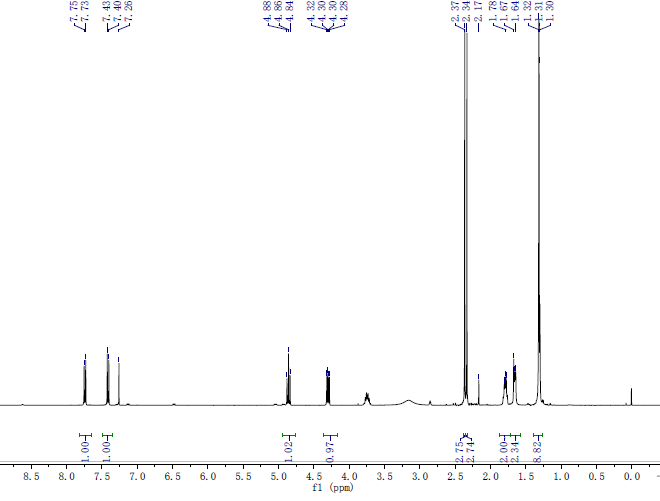


**Figure S31. The ^13^C NMR spectra copy of compound 21 in CDCl_3_ (101 MHz)**


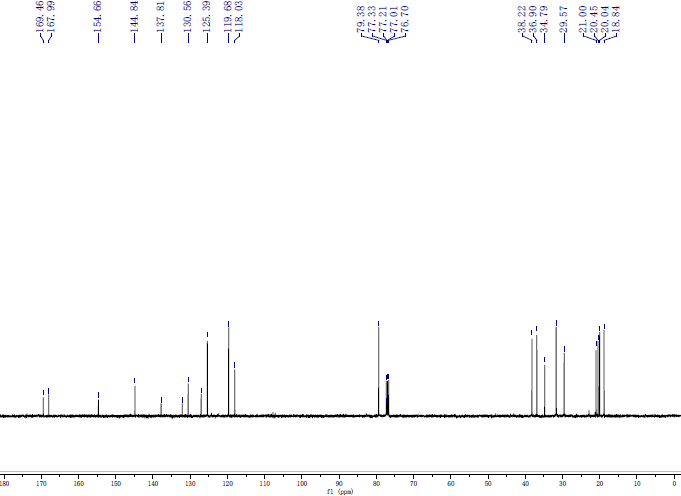


**Figure S32. The ^1^H NMR spectra copy of compound 22 in CDCl_3_ (400 MHz)**


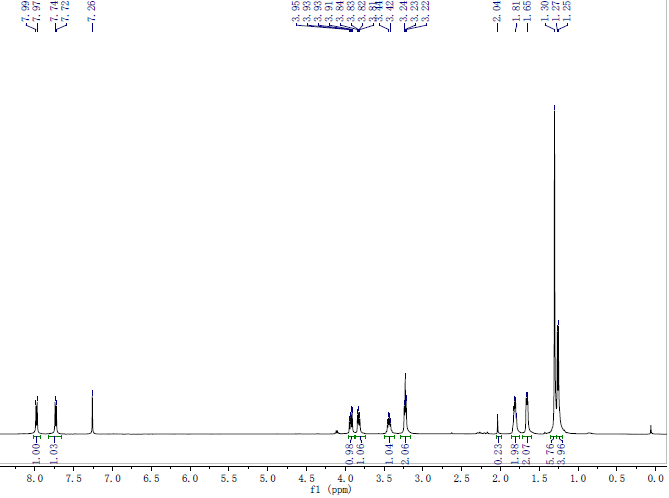


**Figure S33. The ^13^C NMR spectra copy of compound 22 in CDCl_3_ (101 MHz)**


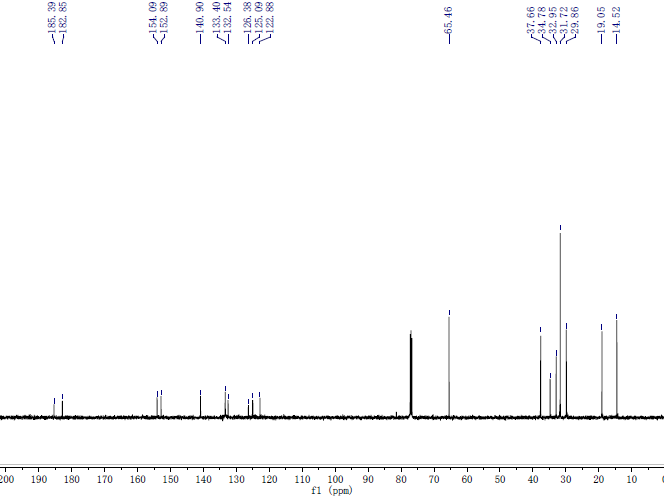


**Figure S34. The ^1^H NMR spectra copy of compound 24 in CDCl_3_ (400 MHz)**


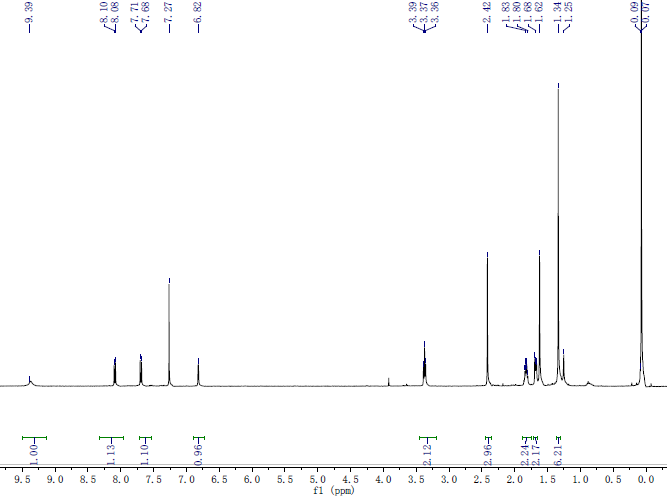


**Figure S35. The ^13^C NMR spectra copy of compound 24 in CDCl_3_ (101 MHz)**


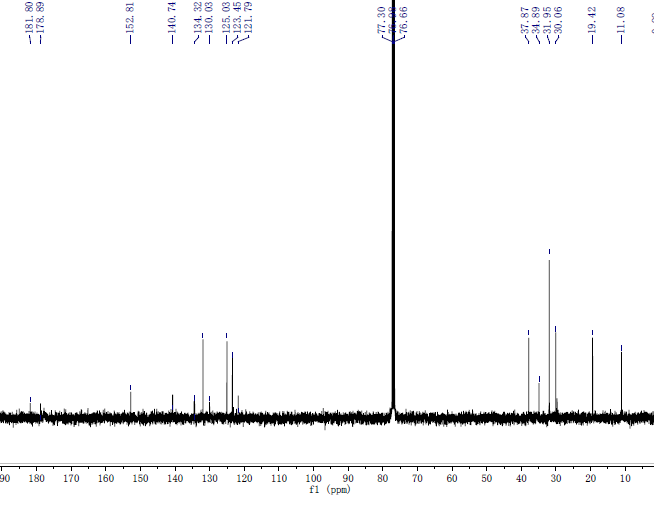


**Figure S36. The ^1^H NMR spectra copy of compound 25 in CD_3_OD (400 MHz)**


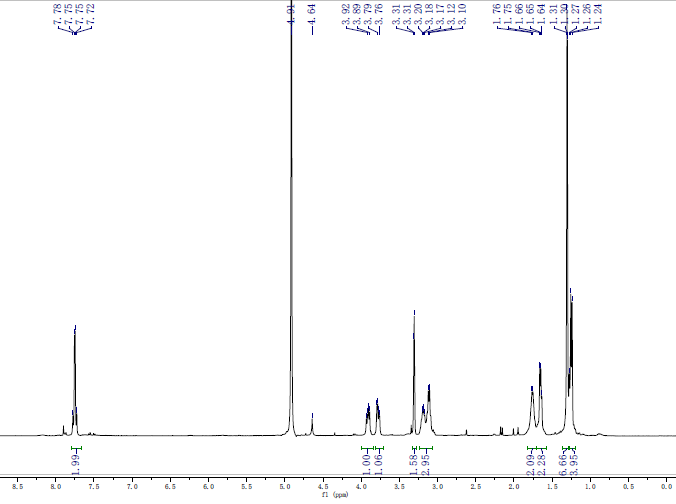


**Figure S37. The ^13^C NMR spectra copy of compound 25 in CD_3_OD (101 MHz)**


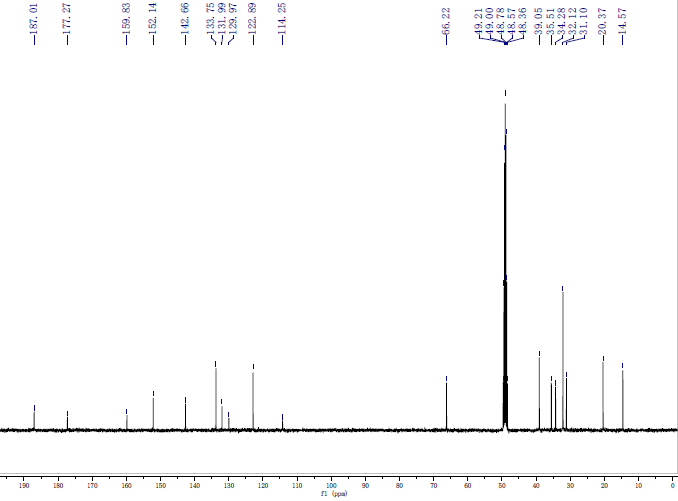


**Figure S38. The ^1^H NMR spectra copy of compound 26 in *d*6-DMSO (500 MHz)**


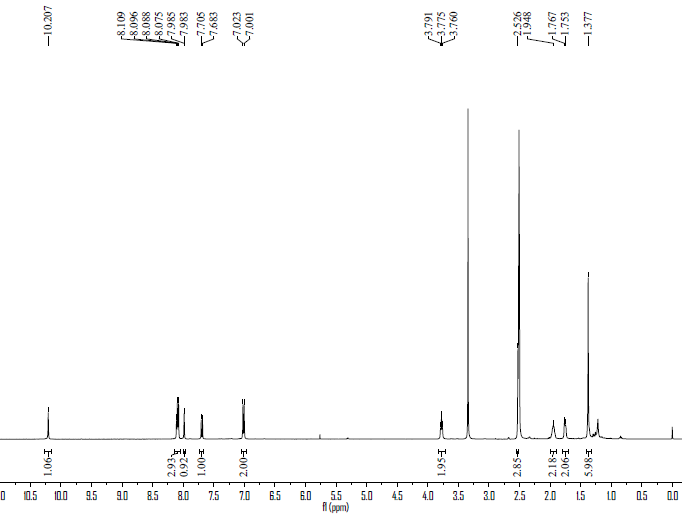


**Figure S39. The ^13^C NMR spectra copy of compound 26 in *d*6-DMSO (125 MHz)**


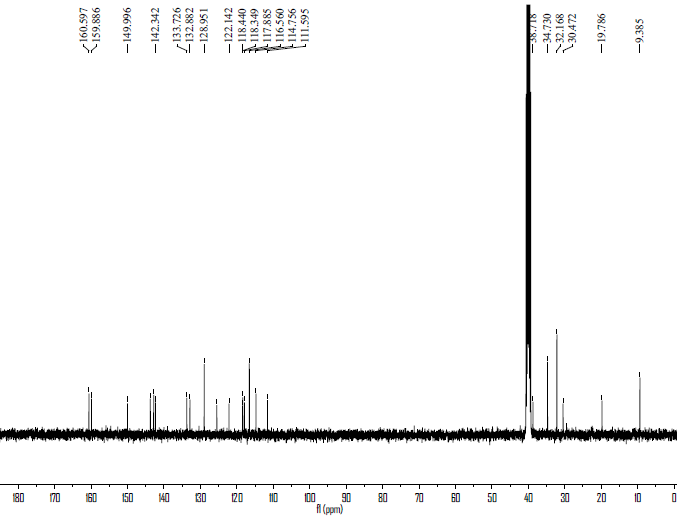


**Figure S40. The ^1^H NMR spectra copy of compound 27a in CDCl_3_ (400 MHz)**


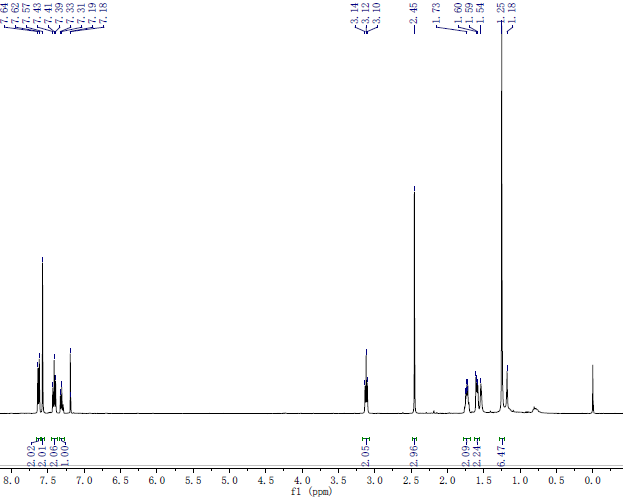


**Figure S41. The ^13^C NMR spectra copy of compound 27a in CDCl_3_ (101 MHz)**


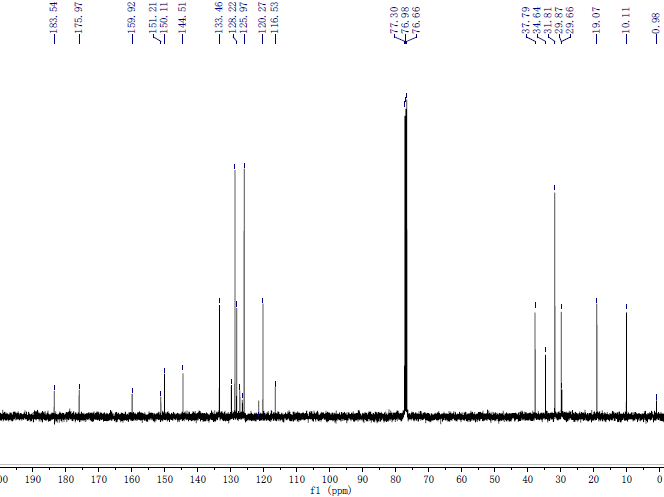


**Figure S42. The ^1^H NMR spectra copy of compound 27b in CDCl_3_ (400 MHz)**


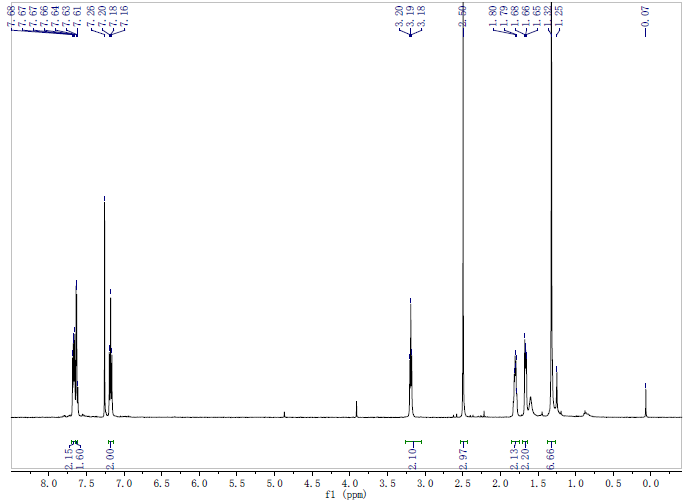


**Figure S43. The ^13^C NMR spectra copy of compound 27b in CDCl_3_ (101 MHz)**


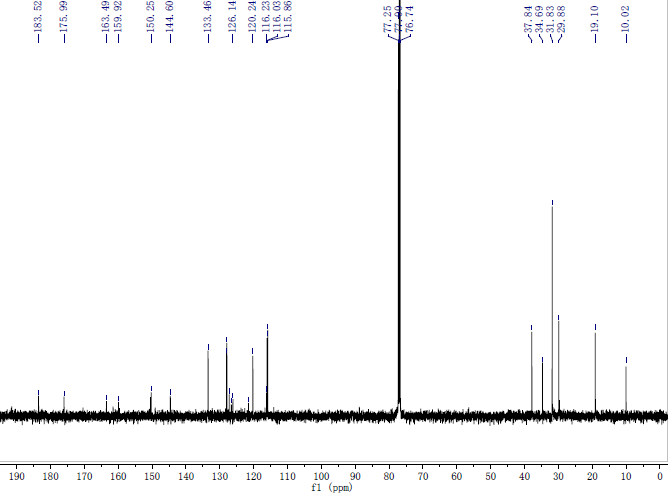


**Figure S44. The ^1^H NMR spectra copy of compound 27c in CDCl_3_ (400 MHz)**


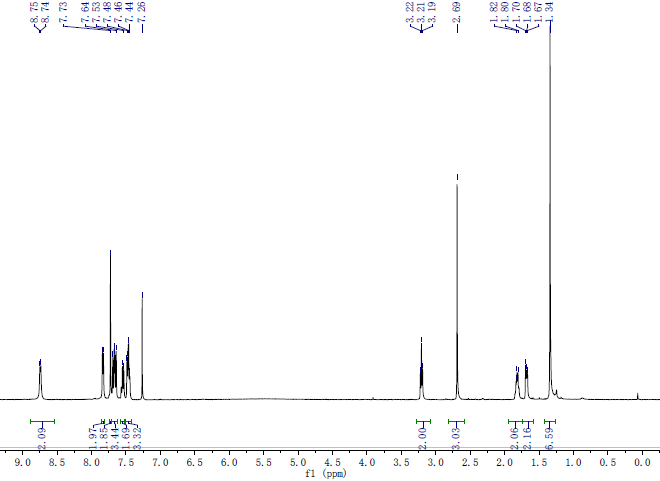


**Figure S45. The ^13^C NMR spectra copy of compound 27c in CDCl_3_ (101 MHz)**


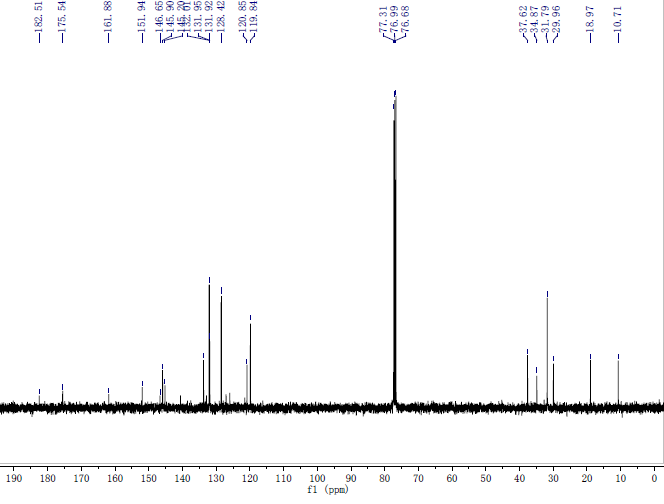


**Figure S46. The ^1^H NMR spectra copy of compound 27d in CDCl_3_ (400 MHz)**


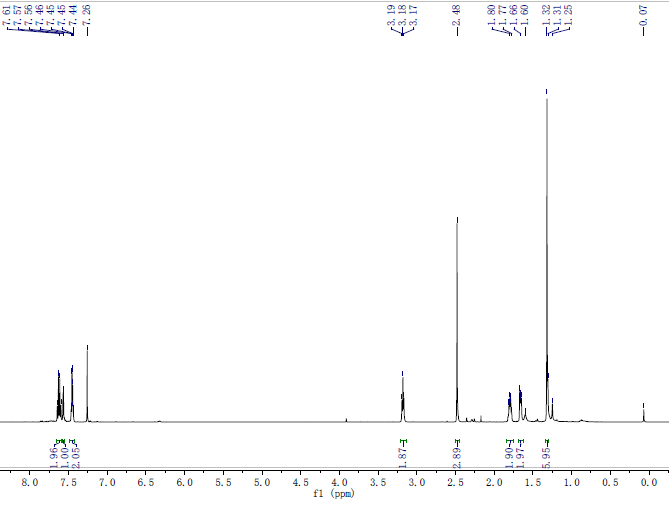


**Figure S47. The ^13^C NMR spectra copy of compound 27d in CDCl_3_ (101 MHz)**


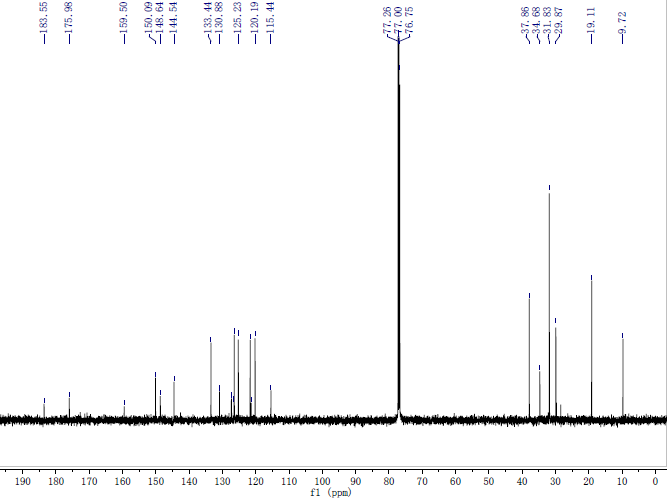


**Figure S48. The ^1^H NMR spectra copy of compound 27e in CDCl_3_ (400 MHz)**


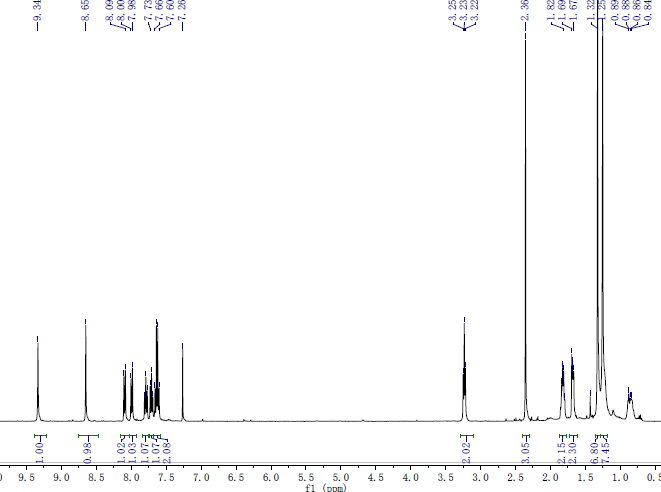


**Figure S49. The ^13^C NMR spectra copy of compound 27e in CDCl_3_ (101 MHz)**


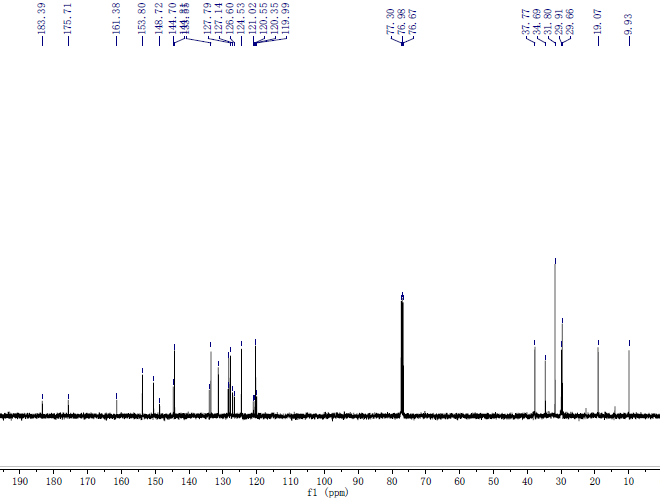


**Figure S50. The ^1^H NMR spectra copy of compound 28a in CDCl_3_ (400 MHz)**


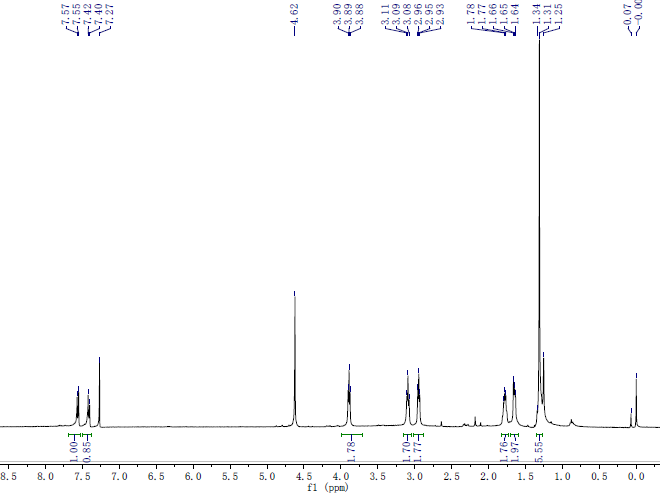


**Figure S51. The ^13^C NMR spectra copy of compound 28a in CDCl_3_ (101 MHz)**


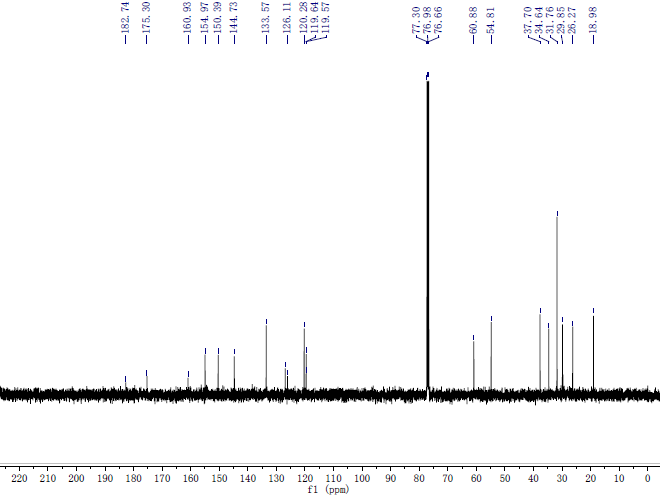


**Figure S52. The ^1^H NMR spectra copy of compound 28b in CDCl_3_ (400 MHz)**


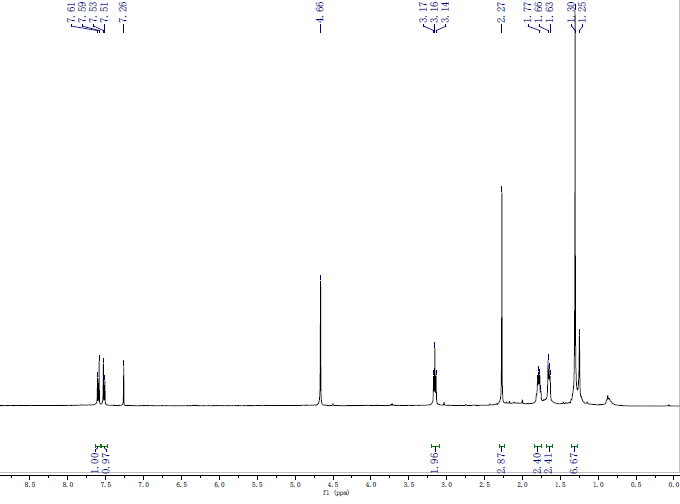


**Figure S53. The ^13^C NMR spectra copy of compound 28b in CDCl_3_ (101 MHz)**


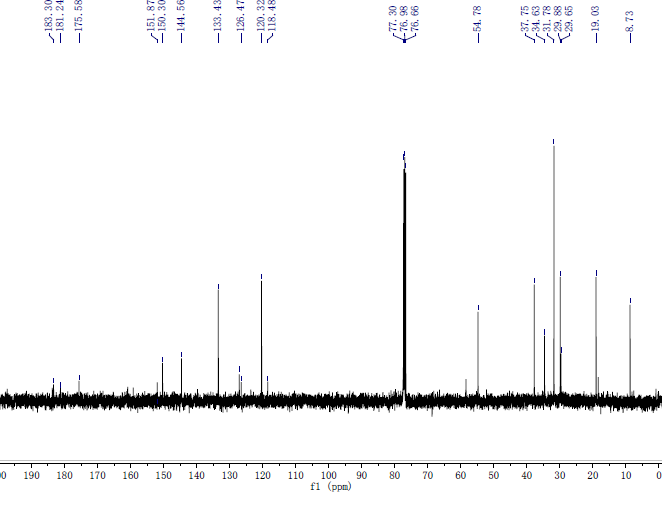


**Figure S54. The ^1^H NMR spectra copy of compound 29a in CDCl_3_ (400 MHz)**


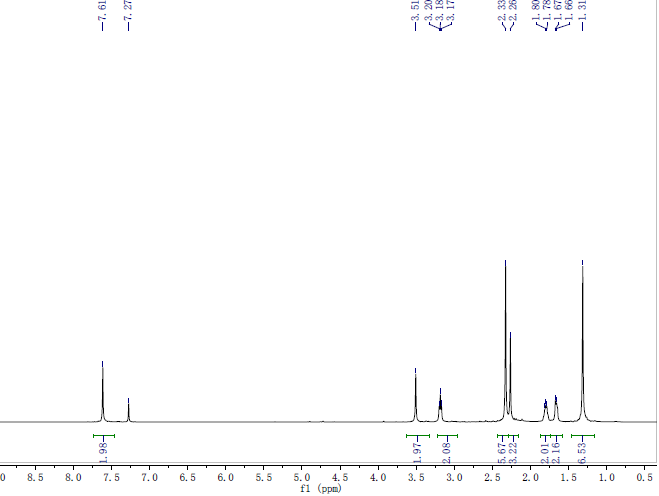


**Figure S55. The ^13^C NMR spectra copy of compound 29a in CDCl_3_ (101 MHz)**


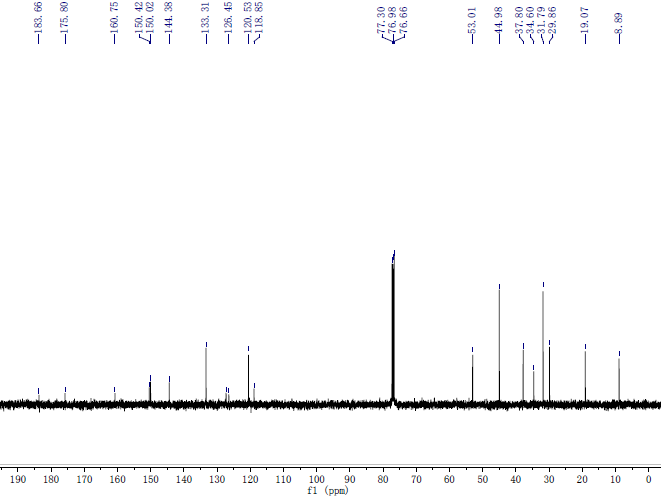


**Figure S56. The ^1^H NMR spectra copy of compound 29b in CDCl_3_ (400 MHz)**


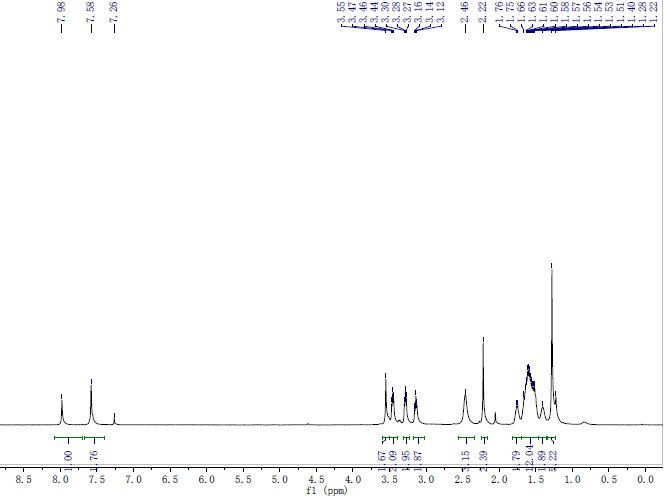


**Figure S57. The ^13^C NMR spectra copy of compound 29b in CDCl_3_ (101 MHz)**


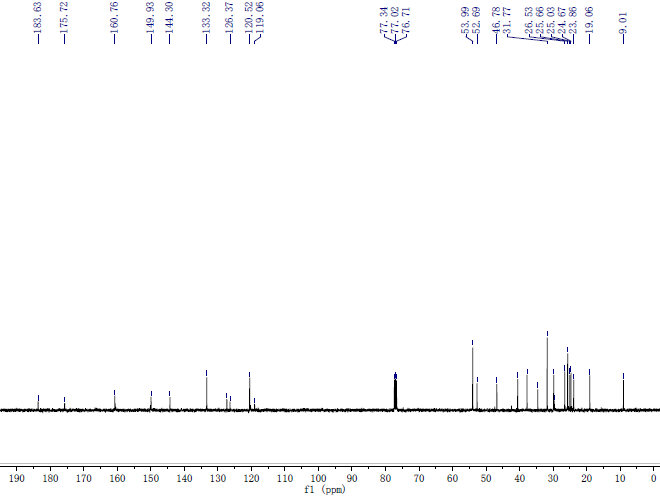


**Figure S58. The ^1^H NMR spectra copy of compound 29c in CDCl_3_ (400 MHz)**


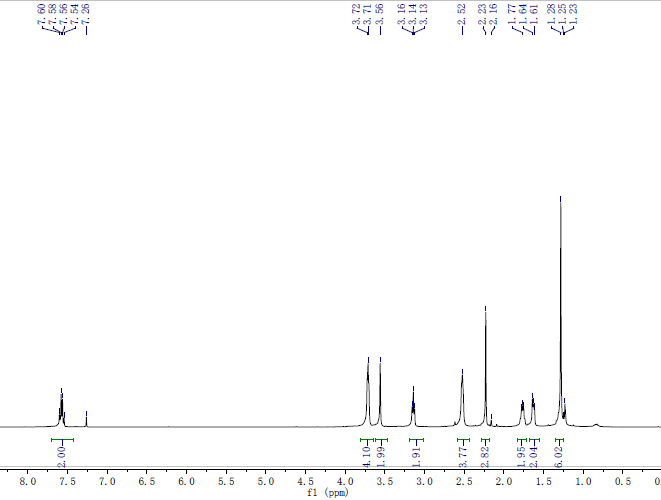


**Figure S59. The ^13^C NMR spectra copy of compound 29c in CDCl_3_ (101 MHz)**


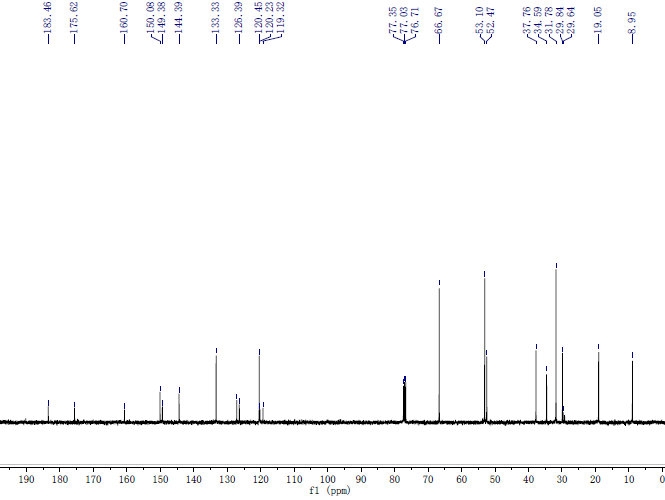


**Figure S60. The ^1^H NMR spectra copy of compound 29d in CDCl_3_ (400 MHz)**


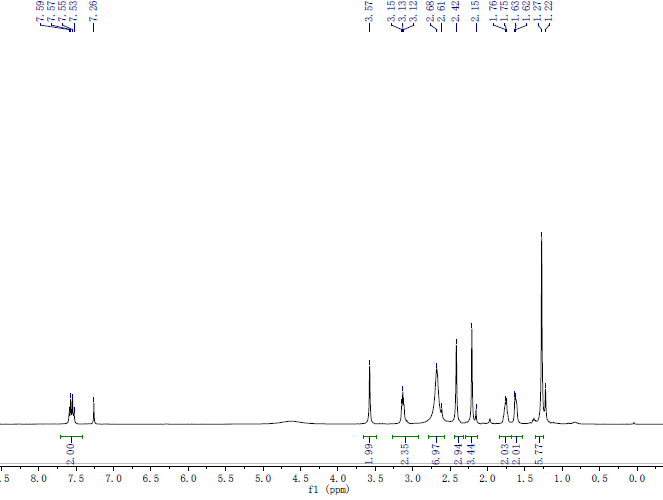


**Figure S61. The ^13^C NMR spectra copy of compound 29d in CDCl_3_ (101 MHz)**


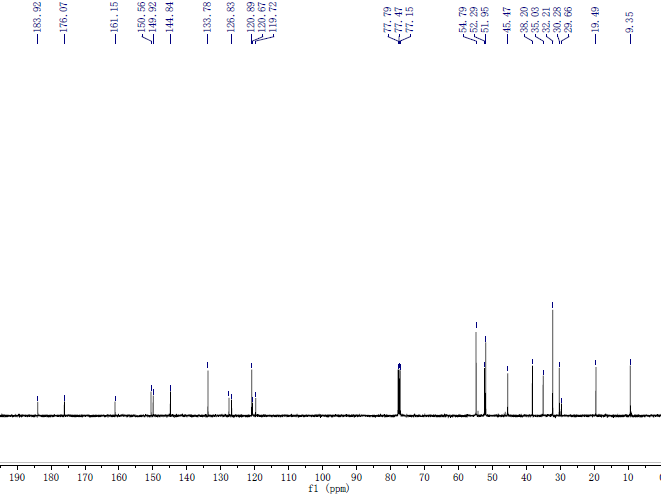


**Figure S62. The ^1^H NMR spectra copy of compound 30 in CDCl_3_ (400 MHz)**


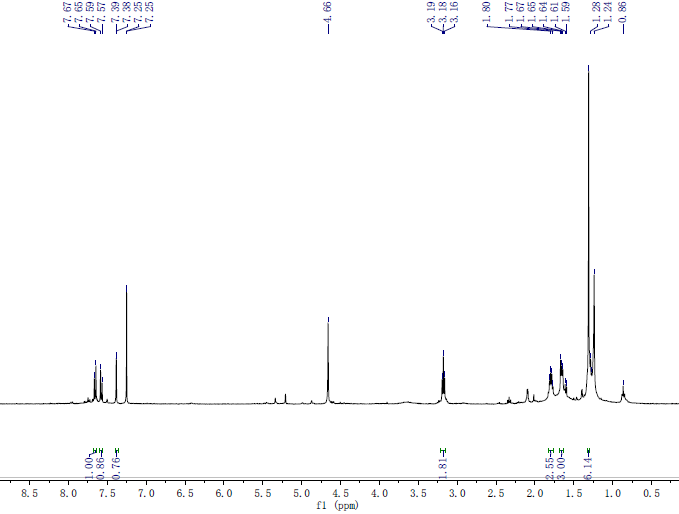


**Figure S63. The ^13^C NMR spectra copy of compound 30 in CDCl_3_ (101 MHz)**


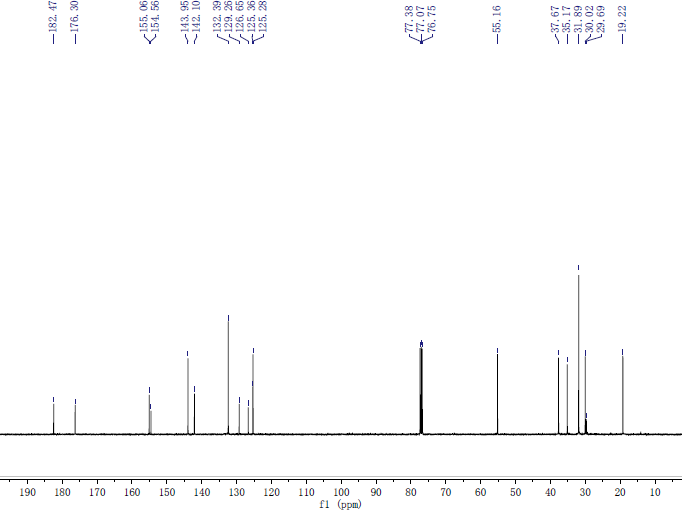


**Figure S64. The ^1^H NMR spectra copy of compound 31a in CDCl_3_ (400 MHz)**


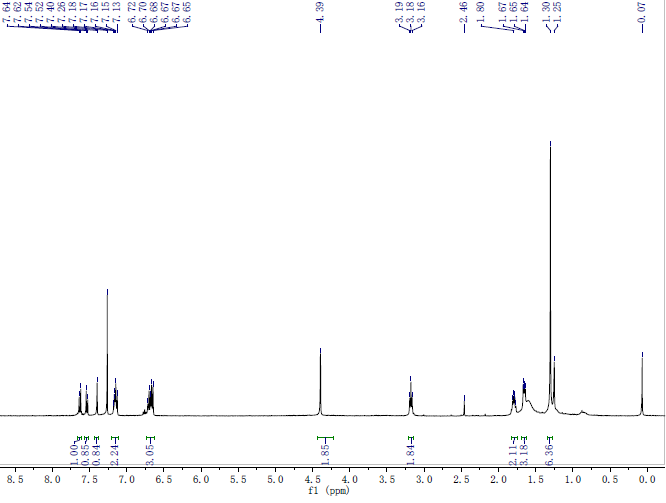


**Figure S65. The ^13^C NMR spectra copy of compound 31a in CDCl_3_ (101 MHz)**


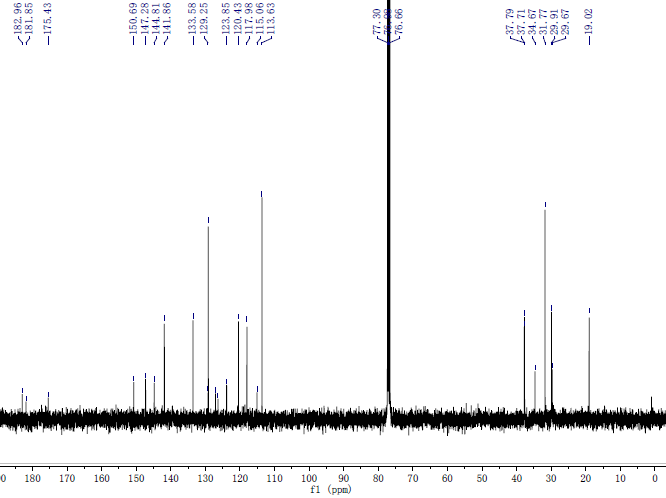


**Figure S66. The ^1^H NMR spectra copy of compound 31b in CDCl_3_ (400 MHz)**


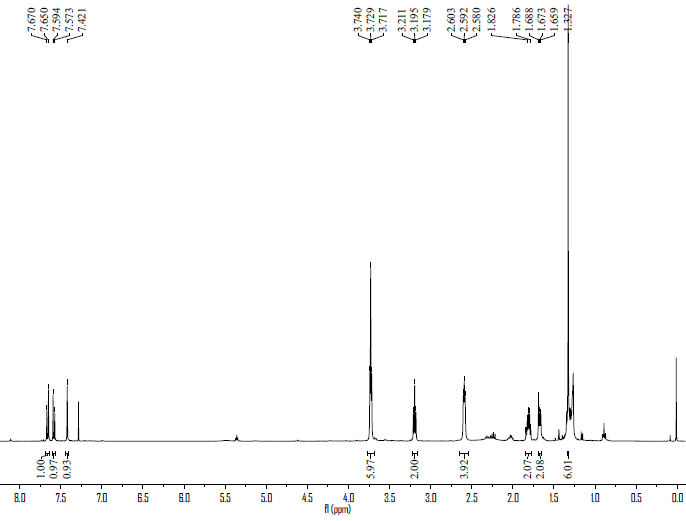


**Figure S67. The ^13^C NMR spectra copy of compound 31b in CDCl_3_ (101 MHz)**


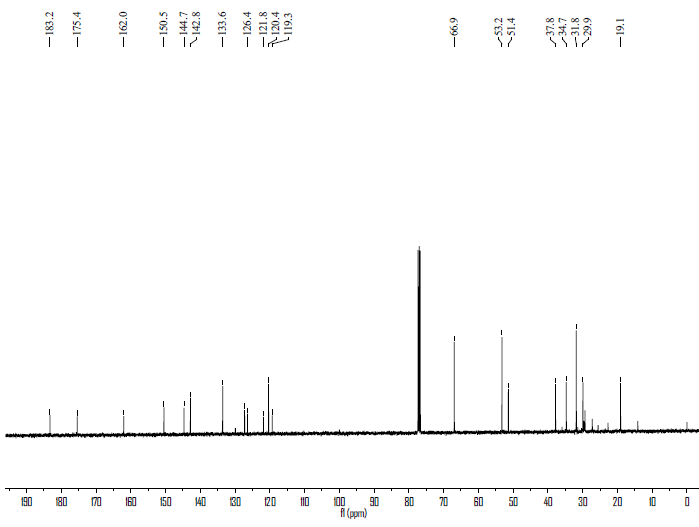


**Figure S68. The ^1^H NMR spectra copy of compound 32 in CDCl_3_ (400 MHz)**


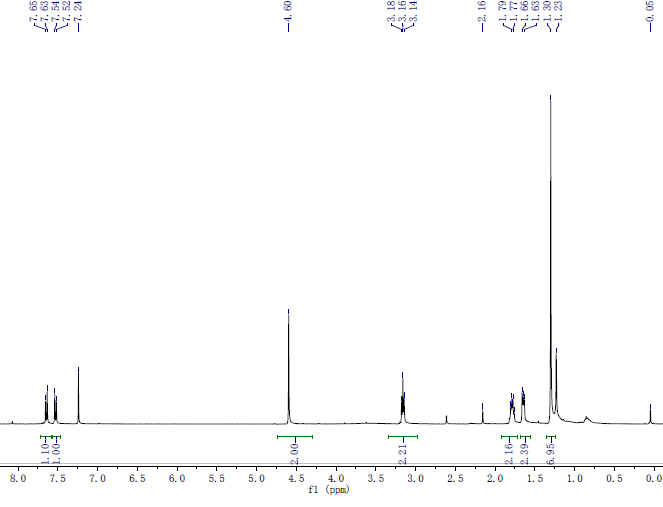


**Figure S69. The ^13^C NMR spectra copy of compound 32 in CDCl_3_ (101 MHz)**

**Figure S70. The ^1^H NMR spectra copy of compound 33 in CDCl_3_ (400 MHz)**


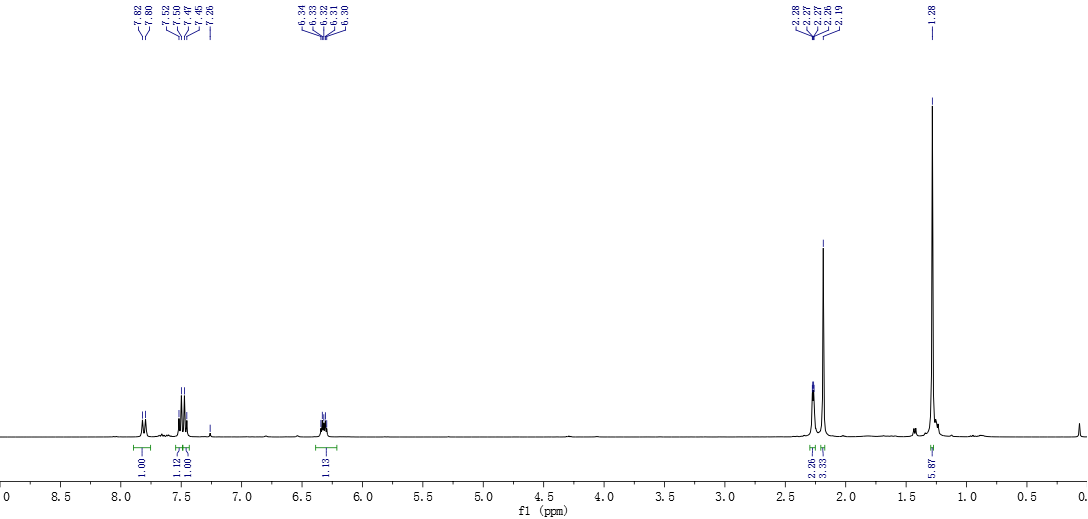


**Figure S71. The ^13^C NMR spectra copy of compound 33 in CDCl_3_ (101 MHz)**


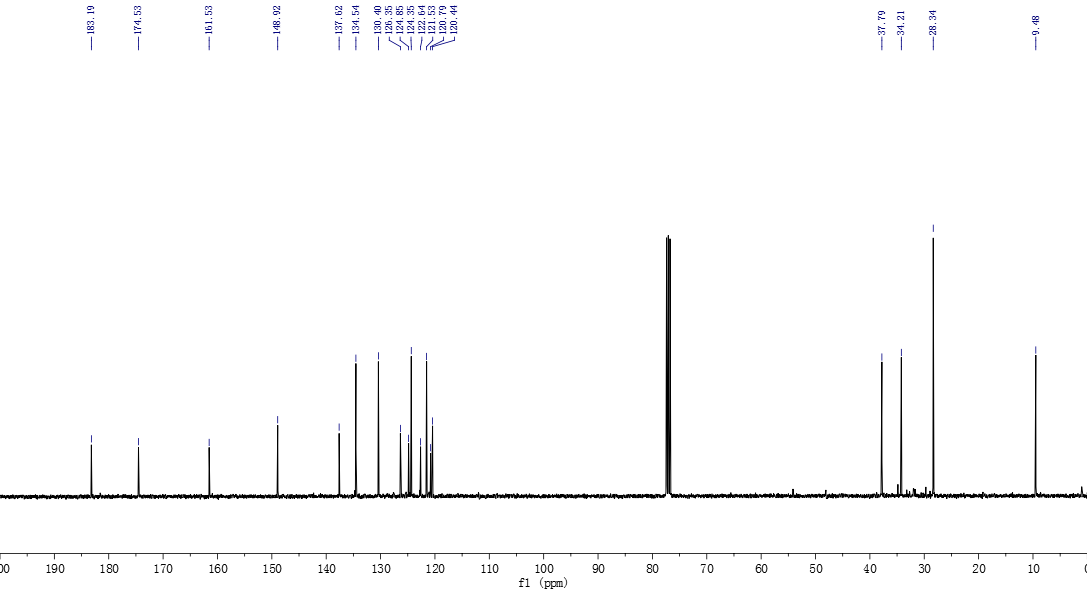


**Figure S72. The ^1^H NMR spectra copy of compound 34 in CDCl_3_ (400 MHz)**


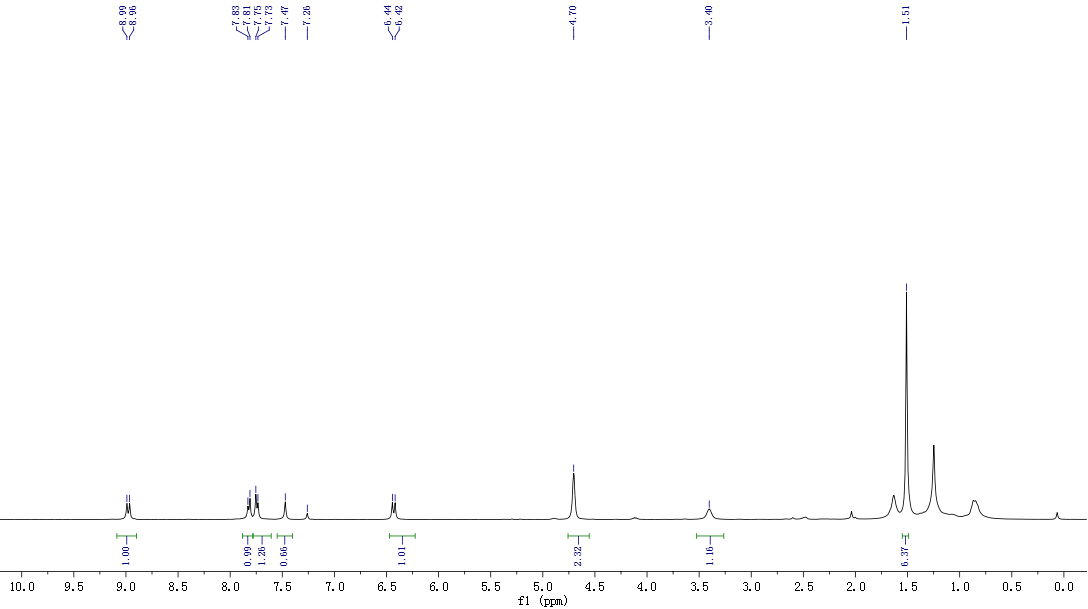


**Figure S73. The ^13^C NMR spectra copy of compound 34 in CDCl_3_ (101 MHz)**


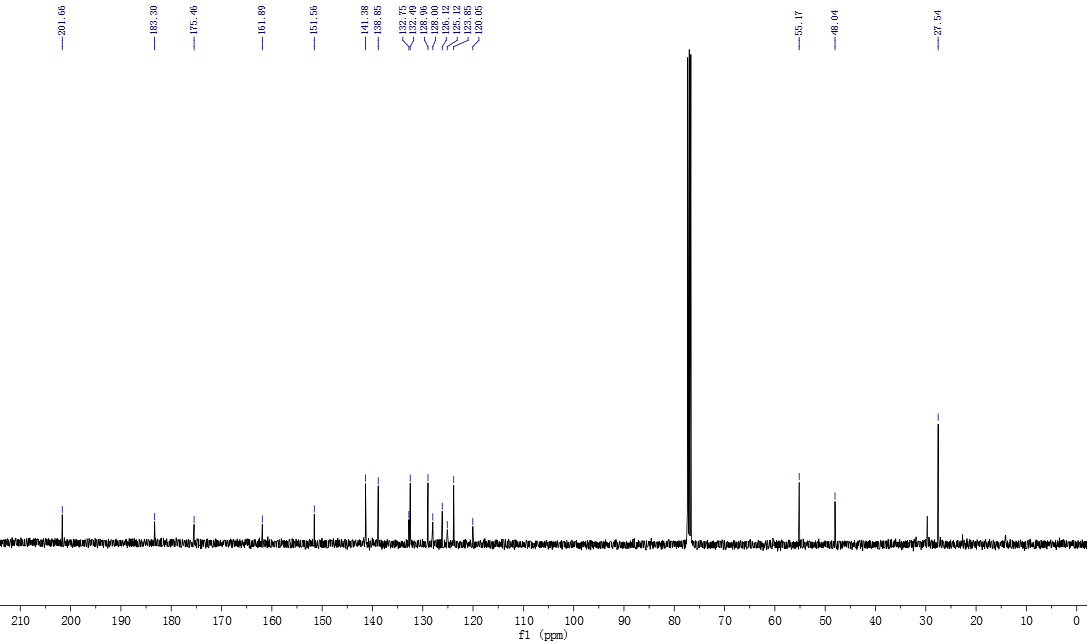


**Figure S74. The ^1^H NMR spectra copy of compound 35 in CDCl_3_ (400 MHz)**


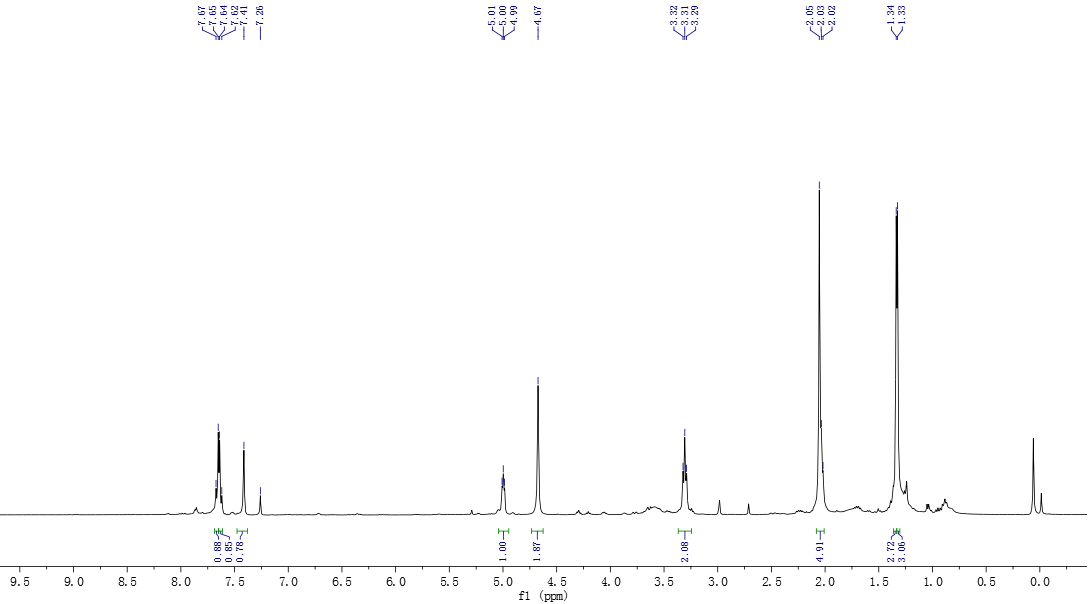


**Figure S75. The ^13^C NMR spectra copy of compound 35 in CDCl_3_ (101 MHz)**


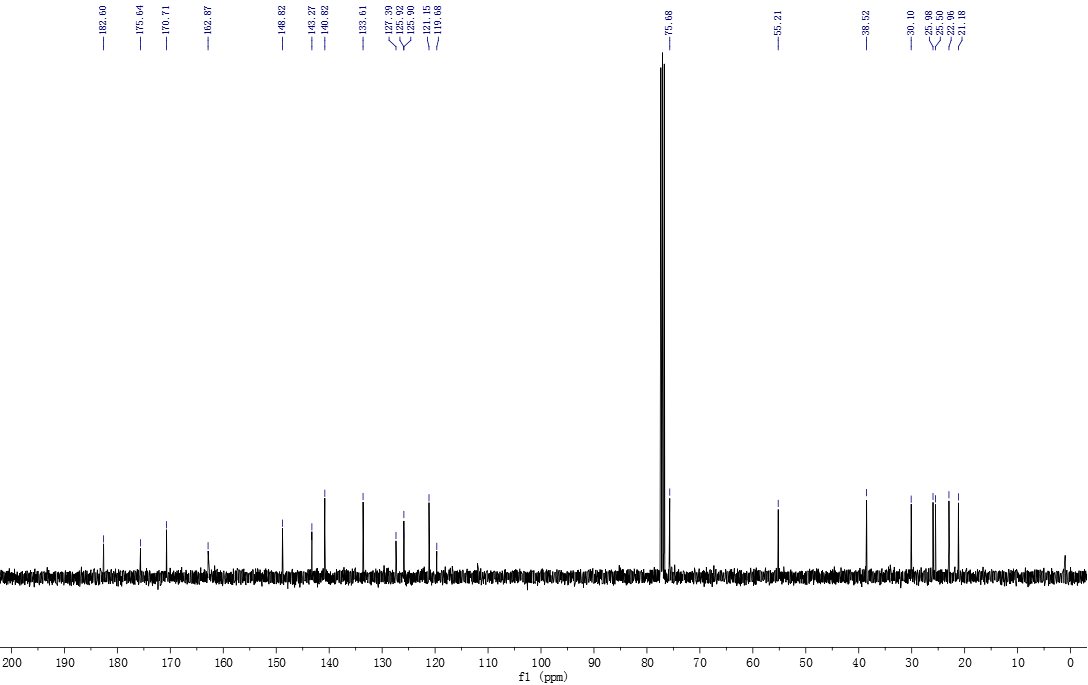


**References**

[1] S.Y. Ryu, Z. No, S.H. Kim, J.W. Ahn, Two Novel Abietane Diterpenes from Salvia miltiorrhiza, Planta Med., 63 (1997) 44-46.

[2] K. Takiura, K. Koizumi, Components of the Chinese drug tanshin. IV. The structure of tanshinone-IIA, Chem. Pharm. Bull., 10 (1962) 112-116.

[3] J.-Y. Park, J.H. Kim, Y.M. Kim, H.J. Jeong, D.W. Kim, K.H. Park, H.-J. Kwon, S.-J. Park, W.S. Lee, Y.B. Ryu, Tanshinones as selective and slow-binding inhibitors for SARS-CoV cysteine proteases, Bioorg. Med. Chem., 20 (2012) 5928-5935.

[4] G. Honda, Y. Koezuka, M. Tabata, Isolation of an antidermatophytic substance from the root of Salvia miltiorrhiza, Chem. Pharm. Bull., 36 (1988) 408-411.

[5] G. Xu, L.-Y. Peng, L. Lu, Z.-Y. Weng, Y. Zhao, X.-L. Li, Q.-S. Zhao, H.-D. Sun, Two new abietane diterpenoids from Salvia yunnanensis, Planta Med., 72 (2006) 84-86.

[6] Z.-H. Pan, Y.-Y. Wang, M.-M. Li, G. Xu, L.-Y. Peng, J. He, Y. Zhao, Y. Li, Q.-S. Zhao, Terpenoids from Salvia trijuga, J. Nat. Prod., 73 (2010) 1146-1150.

[7] M. Vergnes, A. Boehrer, S. Reibel, S. Simler, C. Marescaux, Selective susceptibility to inhibitors of GABA synthesis and antagonists of GABAA receptor in rats with genetic absence epilepsy, Exp. Neurol., 161 (2000) 714-723.

[8] J. Bian, B. Deng, L. Xu, X. Xu, N. Wang, T. Hu, Z. Yao, J. Du, L. Yang, Y. Lei, X. Li, H. Sun, X. Zhang, Q. You, 2-Substituted 3-methylnaphtho[1,2-b]furan-4,5-diones as novel L-shaped ortho-quinone substrates for NAD(P)H:quinone oxidoreductase (NQO1), Eur. J. Med. Chem., 82 (2014) 56-67.

[9] J. Bian, X. Li, N. Wang, X. Wu, Q. You, X. Zhang, Discovery of quinone-directed antitumor agents selectively bioactivated by NQO1 over CPR with improved safety profile, Eur. J. Med. Chem., 129 (2017) 27-40.

[10] F.S. Senol, S. Slusarczyk, A. Matkowski, A. Perez-Garrido, F. Giron-Rodriguez, J.P. Ceron-Carrasco, H. den-Haan, J. Pena-Garcia, H. Perez-Sanchez, K. Domaradzki, I.E. Orhan, Selective in vitro and in silico butyrylcholinesterase inhibitory activity of diterpenes and rosmarinic acid isolated from Perovskia atriplicifolia Benth. and Salvia glutinosa L, Phytochemistry, 133 (2017) 33-44.

[11] S.E. Kurhade, A.I. Sanchawala, V. Ravikumar, D. Bhuniya, D.S. Reddy, Total Synthesis of Isofregenedadiol, Org. Lett., 13 (2011) 3690-3693.

[12] J. Lu, H.-P. Song, P. Li, P. Zhou, X. Dong, J. Chen, Screening of direct thrombin inhibitors from Radix Salviae Miltiorrhizae by a peak fractionation approach, J. Pharm. Biomed. Anal., 109 (2015) 85-90.

[13] D.-L. Zhang, L.-Y. Zhou, J.-M. Quan, W. Zhang, L.-Q. Gu, Z.-S. Huang, L.-K. An, Oxygen Insertion of o-Quinone under Catalytic Hydrogenation Conditions, Org. Lett., 15 (2013) 1162-1165.

[14] L.-W. Zeng, C.-X. Zhou, J.-D. Liu, C.-H. Liu, J.-X. Mo, A.F. Hou, W. Yao, Z.-Z. Wang, L.-S. Gan, Design, synthesis, and antimicrobial activities of new tanshinone IIA esters, Nat. Prod. Res., 30 (2016) 2662-2668.

[15] J. Marrero, Iacute, G. n, Aacute, Lez, Andr, Eacute, L. S, iacute, S. a, J.G. Luis, eacute, rrez, Quinone Derivatives by Chemical Transformations of 16-Hydroxycarnosol from Salvia Species, Chem. Pharm. Bull., 53 (2005) 1524-1529.

[16] L.-K. An, X.-Z. Bu, H.-Q. Wu, X.-D. Guo, L. Ma, L.-Q. Gu, Reaction of tanshinones with biogenic amine metabolites in vitro, Tetrahedron, 58 (2002) 10315-10321.

[17] Y. Xue, Y. Wu, H. Zhu, X.-N. Li, J.-F. Qian, Y. Lai, C. Chen, G. Yao, Z. Luo, Y. Li, Y. Zhang, Salviprzols A and B, C21- and C22-terpenoids from the roots of Salvia przewalskii Maxim, Fitoterapia, 99 (2014) 204-210.

1. ^*^ Corresponding authors. QSZ: Tel. +86-871-65223058; Fax +86 871 65215783; E-mail: qinshizhao@mail.kib.ac.cn; YL: Fax +86-21-50806059; E-mail: yleng@simm.ac.cn; ZZL: Email:zuozhili@mail.kib.ac.cn [↑](#footnote-ref-2)
2. ^*^ [↑](#footnote-ref-3)
3. ^*^ [↑](#footnote-ref-4)
